# Effect of Minocycline on Depressive Symptoms in Patients With Treatment-Resistant Depression: A Randomized Clinical Trial

**DOI:** 10.1001/jamanetworkopen.2022.30367

**Published:** 2022-09-14

**Authors:** Julian Hellmann-Regen, Vera Clemens, Michael Grözinger, Johannes Kornhuber, Andreas Reif, David Prvulovic, Roberto Goya-Maldonado, Jens Wiltfang, Oliver Gruber, Cornelius Schüle, Frank Padberg, Marcus Ising, Manfred Uhr, Tim Friede, Cynthia Huber, André Manook, Thomas C. Baghai, Rainer Rupprecht, Isabella Heuser

**Affiliations:** 1 Department of Psychiatry and Neurosciences, Charité - Universitätsmedizin Berlin, Campus Benjamin Franklin, Germany; 2 Department of Psychiatry, Psychotherapy and Psychosomatics, University Hospital RWTH Aachen, Germany; 3 Department of Psychiatry and Psychotherapy, University Hospital Erlangen, Friedrich-Alexander Universität Erlangen, Erlangen, Germany; 4 Department of Psychiatry, Psychosomatic Medicine and Psychotherapy, University Hospital Frankfurt, Germany; 5 Department of Psychiatry, University Medical Center Göttingen, Germany; 6 Department of Psychiatry, Heidelberg University Hospital, Germany; 7 Department of Psychiatry and Psychotherapy, University Hospital, LMU Munich, Munich, Germany; 8 Max Planck Institute of Psychiatry, Munich, Germany; 9 Department of Medical Statistics, Universitätsmedizin Göttingen, Göttingen, Germany; 10 Department of Psychiatry, University Hospital, Regensburg, Germany

## Abstract

**Importance:**

Insufficient treatment response and resulting chronicity constitute a major problem in depressive disorders. Remission rates range as low as 15% to 40% and treatment-resistant depression (TRD) is associated with low-grade inflammation, suggesting anti-inflammatory interventions as a rational treatment strategy. Minocycline, which inhibits microglial activation, represents a promising repurposing candidate in the treatment of TRD.

**Objective:**

To determine whether 6 weeks of minocycline as add-on to antidepressant treatment as usual can significantly reduce depressive symptoms in patients with TRD.

**Design, Setting, and Participants:**

The study was conducted in Germany and designed as a multicenter double-blind randomized clinical trial (RCT) of 200 mg/d minocycline treatment over a course of 6 weeks with a 6-month follow-up. Participants were recruited from January 2016 to August 2020 at 9 university hospitals that served as study sites. Key inclusion criteria were a diagnosis of major depressive disorder (according to *Diagnostic and Statistical Manual of Mental Disorders* [Fifth Edition] criteria), severity of depressive symptoms on the Hamilton Depression Rating Scale (HAMD-17) greater than or equal to 16 points, aged 18 to 75 years, body mass index 18 to 40, Clinical Global Impression Scale (CGI-S) greater than or equal to 4, failure to adequately respond to an initial antidepressant standard medication as per Massachusetts General Hospital Antidepressant Treatment History Questionnaire, and stable medication for at least 2 weeks. A total of 258 patients were screened, of whom 173 were randomized and 168 were included into the intention-to-treat population. Statistical analysis was performed from April to November 2020.

**Interventions:**

Participants were randomized (1:1) to receive adjunct minocycline (200 mg/d) or placebo for 6 weeks.

**Main Outcomes and Measures:**

Primary outcome measure was the change in Montgomery-Åsberg Depression Rating Scale (MADRS) score from baseline to week 6 analyzed by intention-to-treat mixed model repeated measures. Secondary outcome measures were response, remission, and various other clinical rating scales.

**Results:**

Of 173 eligible and randomized participants (84 randomized to minocycline and 89 randomized to placebo), 168 formed the intention-to-treat sample (79 [47.0%] were women, 89 [53.0%] were men, 159 [94.6%] were White, 9 [6.4%] were of other race and ethnicity, including Asian and unknown ethnicity), with 81 in the minocycline group and 87 in the placebo group. The mean (SD) age was 46.1 (13.1) years, and the mean (SD) MADRS score at baseline was 26.5 (5.0). There was no difference in rates of completion between the minocycline (83.3% [70 of 81]) and the placebo group (83.1% [74 of 87]). Minocycline treatment did not alter the course of depression severity compared with placebo as assessed by a decrease in MADRS scores over 6 weeks of treatment (1.46 [−1.04 to 3.96], *P* = .25). Minocycline treatment also exhibited no statistically significant effect on secondary outcomes.

**Conclusions and Relevance:**

In this large randomized clinical trial with minocycline at a dose of 200 mg/d added to antidepressant treatment as usual for 6 weeks, minocycline was well tolerated but not superior to placebo in reducing depressive symptoms in patients with TRD. The results of this RCT emphasize the unmet need for therapeutic approaches and predictive biomarkers in TRD.

**Trial Registration:**

EU Clinical Trials Register Number: EudraCT 2015-001456-29

## Key Points

**Question:**

Does 6 weeks of minocycline treatment as add-on to standard antidepressant treatment reduce depressive symptoms in patients with treatment-resistant depression?

**Findings:**

In this randomized clinical trial of 168 patients with treatment-resistant depression, 6 weeks of minocycline treatment did not show a statistically significant advantage compared with placebo on the overall course of depressive symptoms.

**Meaning:**

The findings of this randomized clinical trial suggest the need for more effective therapeutic interventions and biomarkers in this heterogeneous clinical condition.

## Teaser

This randomized clinical trial investigates whether 6 weeks of minocycline as an add-on to antidepressant treatment as usual can significantly reduce depressive symptoms in patients with treatment-resistant depression.

## Introduction

Major depressive disorder (MDD) is an important cause of disability worldwide.^1^ Insufficient response rates, persistent residual symptoms, frequent relapse or recurrence of symptoms constitute unsatisfactory outcomes of conventional antidepressant treatments. Response rates for an initial treatment are estimated at roughly 50% with a substantial proportion lacking full remission even following various switching and augmentation strategies of the antidepressant regimen.^2^

Accumulating evidence from clinical and preclinical studies suggests involvement of the immune system and neurotrophic mechanisms as pivotal neurobiological underpinnings in the pathogenesis of MDD.^3,4,5^ Moreover, low-grade inflammatory processes potentially interfere with response to the usual antidepressants.^3,5,6,7^ Clinical evidence for efficacy of anti-inflammatory interventions in MDD is based on nonsteroidal anti-inflammatory drugs (NSAIDs), cytokine-inhibiting drugs and statins, with studies showing varying efficacy and large heterogeneity in trial designs.^8,9^ While some NSAIDs carry a risk for adverse events such as infections or hemorrhage in combination with serotonergic antidepressants, selective COX-2 inhibiting drugs appear to exhibit only transient efficacy, if at all.^10^ Targeting inflammatory processes in a more specific manner may provide a promising treatment rationale, particularly for treatment-resistant depression.^7,11^

Minocycline is a tetracycline antibiotic with pleiotropic antineuroinflammatory properties that readily crosses the blood-brain barrier, inhibits proinflammatory microglial activation, enhances neuroprotective retinoid signaling, and exerts overall neuroprotective effects.^12,13,14,15^ There is profound evidence for antidepressant effects of minocycline in animal models,^16^ most likely mediated through beneficial effects on microglial activation.^17,18,19,20^ Thus, minocycline treatment has been suggested as a novel antidepressant approach^21^ and indeed, a few small randomized clinical trials (RCTs) suggest antidepressant efficacy of minocycline in MDD patients.^22^ Emadi-Kouchak and colleagues showed that patients with HIV with mild-to-moderate depression who underwent a 6-week treatment with 100 mg minocycline exhibited larger improvement of depressive symptoms compared with the placebo group.^23^ In another trial of 71 patients with non-treatment-refractory MDD, 12 weeks of treatment with 200 mg minocycline resulted in significantly larger improvements in clinical global impression scores, quality of life- and functioning-related scores in the minocycline vs the placebo group, but remained without effect on the primary depression-related outcome parameter.^24^ With respect to treatment-resistant depression, Husain and colleagues^25^ treated 41 patients with TRD with up to 200 mg minocycline or placebo over a period of 12 weeks. The decrease of depressive symptoms in the minocycline group was significantly larger compared with the placebo group. Another small, recently published trial included 39 patients with TRD selected for at least marginally increased levels of serum C-reactive protein (CRP) greater than or equal to 0.1 mg/dL (to convert to milligrams per liter, multiply by 10).^26^ Exploratory analyses suggested a positive effect of minocycline treatment on depressive symptoms compared with placebo, yet only for a small group of patients with CRP levels of at least 0.3 mg/dL.

However, small sample sizes and heterogeneous populations of previous studies warrant larger, well-controlled trials to demonstrate a putative antidepressant effect of minocycline.^21^ Here, we investigated whether minocycline (200 mg/d) given for 6 weeks as add-on to a stable antidepressant medication reduces depressive symptoms in patients with a major depressive episode classified as treatment resistant.

## Methods

### Study Design

The Minocycline in Treatment-Resistant Depression (MinoTRD) trial was a double-blind, placebo-controlled and randomized multicenter clinical trial investigating whether 200 mg of minocycline hydrochloride per day administered orally for 6 weeks as an adjunct to antidepressant standard treatment reduces depressive symptoms in patients with MDD without, so far, adequate response to an initial antidepressant regimen.

Patients were recruited from inpatient and outpatient departments of 9 university hospitals in Germany that served as study sites. A list of sites and principal investigators is provided in Supplement 1. The first patient was recruited in January 2016, and the last visit was completed in August 2020. Key inclusion criteria were a current diagnosis of MDD according to *Diagnostic and Statistical Manual of Mental Disorders* (Fifth Edition) criteria, aged between 18 and 75 years, ability to give informed consent for participation, a score of at least 16 points on the 17-item Hamilton rating scale for depression (HAM-D 17), an antidepressant regimen for at least 6 weeks without dose changes within the last 2 weeks prior to inclusion and inadequate response to conventional antidepressant treatment according to the criteria of the Massachusetts General Hospital antidepressant treatment response questionnaire (MGH-ATRQ). Ethnicity was assessed by self-report. Race and ethnicity were assessed because they may affect pharmakokinetics. Key exclusion criteria were prevalence of neurodegenerative disorder, any severe, unstable general medical condition, including chronic inflammatory disease, concurrent anti-inflammatory medication, pregnant or nursing women and tetracycline allergy. To improve recruitment, criteria were slightly adapted. In addition to antidepressant monotherapy, therapy with more than 1 antidepressant was permitted. Details on inclusion and exclusion criteria are presented in Supplement 1. The MinoTRD trial was conducted in accordance with the International Conference on Harmonization Good Clinical Practice guidelines and the Declaration of Helsinki.^27^ The trial is registered with the European Union Clinical Trials Register (EudraCT 2015-001456-29) and ClinicalTrials.gov (identifier: NCT02456948). The study protocol, patient information sheets, and informed consent forms were approved by the ethics committee of the state of Berlin (Landesamt für Gesundheit und Soziales Berlin) and the Federal Institute for Drugs and Medical Devices (Bundesinstitut für Arzneimittel und Medizinprodukte, BfArM). This study followed the Consolidated Standards of Reporting Trials (CONSORT) reporting guideline. Participants were randomized to receive minocycline 200 mg/d or placebo. The randomization was carried out centrally using a computerized system based on a permuted block procedure with block stratification by study center.

### Trial Procedures

Study medication was provided as gelatin capsules containing 50 mg of minocycline hydrochloride or identically appearing placebo capsules (Mibe Pharmaceuticals) in blister packs. Medication was dispensed initially and after 3 weeks. Trial group dosing was as follows: minocycline, 200 mg (2 × 50 mg capsules in the morning and 2 × 50 mg in the evening) and placebo (2 placebo capsules in the morning and 2 capsules in the evening). Group assignment was masked to prescribing clinicians, participants, and all trial staff members except pharmacy staff and the trial statistician (TF).

Participants visited the clinic at baseline, and on a weekly basis during the 6 weeks of receiving study medication (treatment period). Prior to inclusion, participants had to be on an antidepressant regimen for at least 6 weeks without dose changes within the last 2 weeks prior to inclusion. Continuous pharmacotherapy with any antidepressant approved for the treatment of depression in a sufficient and stable dosage was mandatory. Concomitant psychotherapy was accepted and concurrent use of lorazepam (maximum: 4 mg/d) or Z-hypnotics (zolpidem [maximum 10 mg/d], zopiclone [maximum 7.5 mg/d]) was permitted as comedication only for treatment of acute agitation and/or anxiety or severe sleep disorders. Additionally, established augmentation strategies with either quetiapine or aripiprazole as well as additional antidepressants outside the indicated target range, for example for improving sleep, were allowed. Follow-up visits took place 6 weeks and 6 months (the latter by telephone) after the end of the treatment period. Routine laboratory safety analyses were performed on a weekly basis, additional biosamples (peripheral blood mononuclear cells, plasma, and serum) were collected. Minocycline serum level analyses were performed by liquid chromatography and mass spectrometry on samples from baseline, week 2, week 4, and week 6 at the end of the trial and after finalization of the database. Additional information on adherence was gathered at each assessment and through dispensing records. Outcome assessments and adverse events were recorded at each visit.

### Outcome Measures

Primary outcome was the change of depression severity from baseline to week 6, assessed by the Montgomery-Åsberg Depression Rating Scale (MADRS).^28^ Secondary outcomes were response (50% reduction in MADRS score) and remission (MADRS score <9) after 6 weeks of treatment. Change on HAMD-17,^29^ Beck Depression Inventory (BDI),^30^ Clinical Global Impressions Scale (CGI-S),^31^ Trail Making Tests (TMT) A and B and Symptom Checklist 90-R (SCL-90-R)^32^ were also assessed weekly and evaluated after 6 weeks of treatment. Exploratory outcomes included subscales and stratification for inflammation-associated parameters or gender as well as follow up measurements outside of the core treatment period of 6 weeks.

### Statistical Analysis

Based on a standardized mean difference (Cohen *d*) of 0.5 as suggested by a similar study,^33^ a sample size of 64 patients per group yields a power of 80% at a a 1-sided significance level of 2.5% with a 2-sample *t* test for the primary end point (ie, change in MADRS from baseline [week 0] to week 6). Adjusting for approximately 20% dropout results in a target group size of 80 participants per study group and a total case number of 160. As the mixed model for repeated measures (MMRM) approach is used for the analysis of the primary end point, which is more efficient than the 2-sample *t* test, the power was expected to be higher than 80% and the approach taken conservative. The sample size calculation was carried out using nQuery Advisor 7.0. The primary evaluation sample follows the intention-to-treat (ITT) principle and includes all patients that received the randomized medication at least once plus had at least 1 postbaseline MADRS score.

Continuous baseline characteristics are given as median (range) or mean (SD) as appropriate and categorical variables as frequencies. The primary end point was change in MADRS from baseline (week 0) to week 6. The primary end point was evaluated by a MMRM and reported by the difference of marginal means of the 2 treatment groups at week 6. The model includes the factors treatment group (placebo, minocycline), study center (center 1 to center 9), time point of measurement (week 1 to week 6) as fixed effects. Other covariates treated as fixed effects were the MADRS sum score at baseline, the time point by MADRS sum score at baseline interaction and the treatment by time point interaction. Patient-specific effects enter the model as a random effect with normal distribution and expected value 0. An unstructured covariance matrix for the residuals was used. The evaluation of the secondary outcomes such as CGI-S, BDI, HAMD-17, and SCL-90 as well as TMT A and B were performed analogously to the evaluation described above for MADRS. Similarly, laboratory parameters such as CRP and leukocyte values were transformed using the natural logarithm and evaluated analogously to the primary end point. The secondary end points of remission and response to treatment were evaluated using logistic regression, with dropouts defined as no remission and no response to treatment, respectively. In the logistic regression model, treatment was included as factor, and MADRS sum score at baseline was included as a covariate.

As exploratory end points HAMD-6 and subscales based on items of MADRS and HAMD measuring a composite for anhedonia, sickness behavior, and fear and/or suicidality^3,34^ were analyzed analogously to the evaluation described for the primary end point. In an exploratory subgroup analysis for severity of depressive symptoms at baseline measured by MADRS, we split the population into 2 subgroups based on the MADRS median value at baseline. We included the resulting variable as factor and its interaction with treatment into the MMRM model described for the primary analysis. The same approach was used for gender, childhood trauma as assessed by the childhood trauma questionnaire (CTQ), and subgroups defined by median-split for baseline CRP and body mass index (BMI) at baseline. Significance was indicated by 2-sided *P* < .05; no correction for multiple testing of exploratory outcomes was applied. All analyses were done in SAS version 9.4 (SAS Institute). For further details, see statistical analysis plan and trial protocol (Supplement 1).

## Results

**Figure 1.**
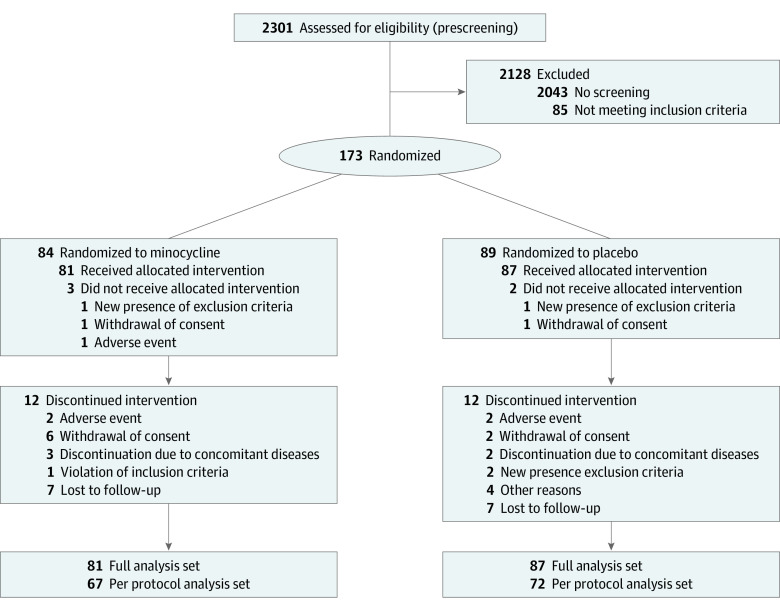
Trial Participant Flow Diagram

Between January 7, 2016, and August 7, 2020, a total of 253 patients were screened for eligibility from 9 different university hospitals in Germany, of whom 168 participants entered the trial (Figure 1). Eighty-nine patients were randomized to placebo, 84 received minocycline. A total of 5 patients did not start trial medication and were thus excluded from all analyses per protocol definition. The ITT sample consisted of 81 patients in the minocycline group and 87 patients in the placebo group. Of the 168 participants, 79 (47.0%) were women, 89 (53.0%) were men, 159 (94.6%) were White, and 9 (6.4%) were of other race and ethnicity, including Asian and unknown ethnicity. The mean (SD) age was 46.1 (13.1) years, and the mean (SD) MADRS score at baseline was 26.5 (5.0). Baseline characteristics of these 168 participants were similar between treatment groups (Table 1). However, there were more female participants in the placebo group (55.2% [n = 48]) compared with the minocycline group (38.3% [n = 31]). Median (range) CRP levels at baseline were 1.2 mg/dL (0.02-22.1) in the minocycline and 0.60 mg/dL (0.05-7.30) in the placebo group (to convert to milligrams per liter, multiply by 10). Participants had a median (range) duration of the present episode of 41 (6-952) weeks in the minocycline and 52 (4-624) weeks in the placebo group (Table 1).

**Table 1.** Baseline Characteristics of the Intention-to-Treat Population

Baseline characteristics	Patients, No. (%)	
	Minocycline (n = 81)	Placebo (n = 87)
Gender		
Female	31 (38.3)	48 (55.2)
Male	50 (61.7)	39 (44.8)
Age, mean (SD)	44.8 (13.6)	47.3 (12.5)
Ethnicity		
White	77 (95.0)	82 (94.3)
Other^a^	4 (4.9)	5 (5.7)
Current smoker	21 (25.9)	25 (28.7)
BMI, mean (SD)	27.4 (5.3)	26.5 (4.8)
Current medication		
SSRI	47 (58.0)	37 (42.5)
SNRI	30 (37.0)	40 (46.0)
MAO inhibitor	4 (4.9)	5 (5.7)
Tricyclic/tetracyclic AD	6 (7.4)	10 (11.5)
Other	29 (35.8)	40 (46.0)
No. of ADs		
1	59 (72.8)	60 (69.0)
2	16 (19.8)	17 (19.5)
>2	6 (7.4)	10 (11.5)
Months on current medication, median (range)	180.0 (19.3-585.0)	90.0 (9.7-540.0)
First depressive episode^b^	14 (18.0)	23 (26.7)
Duration of the current depressive episode, median (range), wk	41 (6-952)	52 (4-624)
No of previous episodes, median (range)	2.5 (0-30)	2 (1-25)
Current inpatient stay or dayclinic	13 (16.7)	13 (15.1)
Depression duration from onset, mean (SD), y	16.52 (12.69)	18.64 (12.02)
Baseline MADRS, mean (SD)	26.4 (4.8)	26.6 (5.1)
Baseline HAMD-17, mean (SD)	20.0 (3.5)	20.3 (3.5)
Baseline BDI II, mean (SD)	32.3 (9.0)	32.9 (10.4)
Baseline SCL-90-R, mean (SD)	68.7 (6.8)	66.9 (6.9)
Baseline CGI, mean (SD)	4.8 (0.6)	4.9 (0.7)
Baseline TMT-A, mean (SD)	34.7 (16.6)	35.1 (14.3)
Baseline TMT-B, mean (SD)	77.5 (32.7)	76.1 (32.3)
Baseline hsCRP, median (range), mg/dL	0.121 (0.002-2.210)	0.060 (0.005-0.730)

Abbreviations: AD, antidepressant; BDI II, Beck Depression Inventory; BMI, body-mass index; CGI, clinical global impression scale; HAMD-17, Hamilton Depression Rating Scale; hsCRP, high sensitivity C-reactive protein; MADRS, Montgomery–Åsberg Depression Rating Scale; SCL-90-R, Symptom Checklist-90-R.

SI conversion factor: To convert hsCRP to milligrams per liter, multiply by 10.

^a^ Other ethnicities included Asian and unknown ethnicity.

^b^ Data not available for all randomized patients.

**Figure 2.**
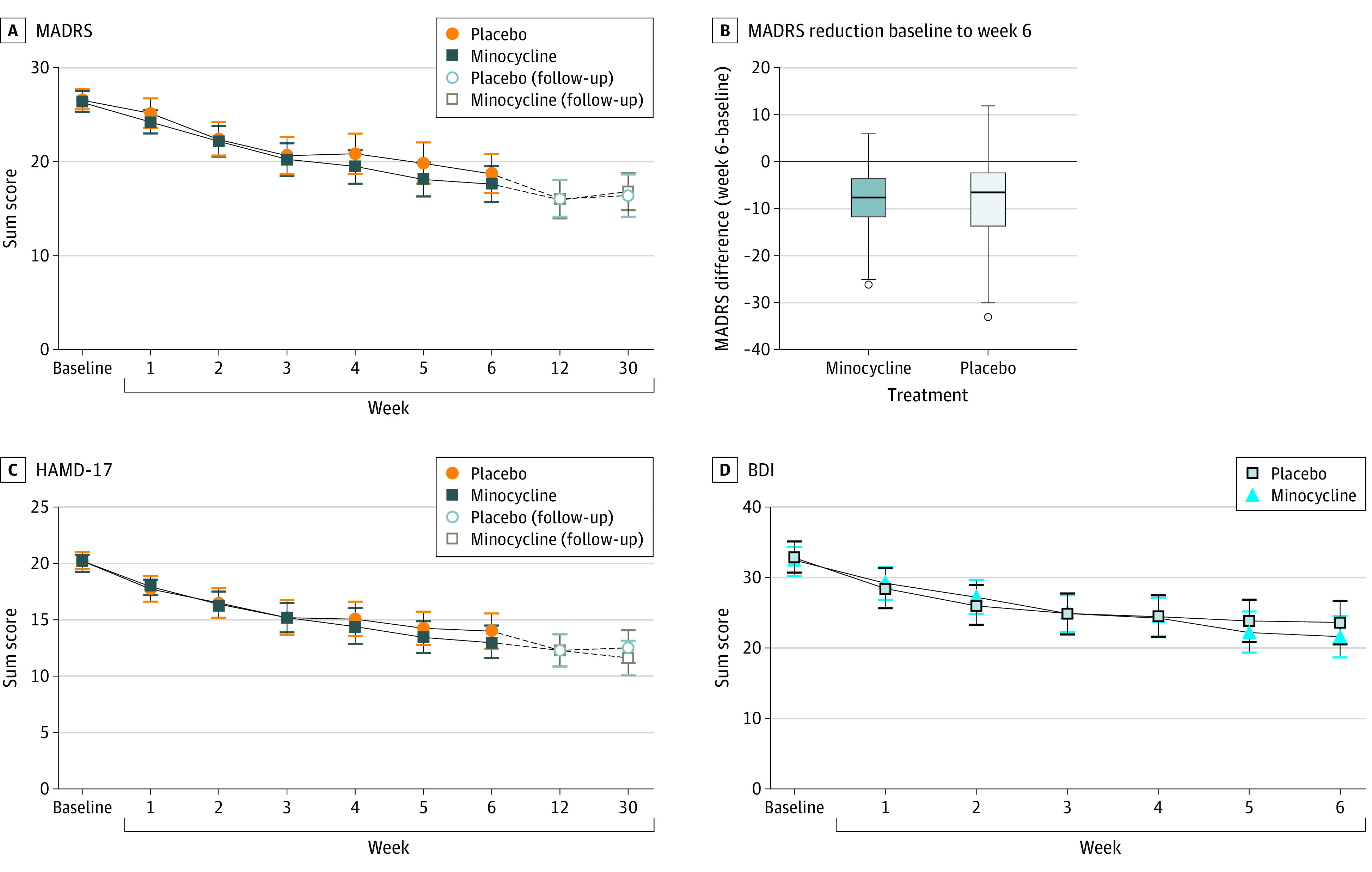
Time Course of Depression Severity BDI indicates Beck Depression Inventory; HAMD-17, Hamilton Depression Rating Scale; MADRS, Montgomery-Åsberg Depression Rating Scale. Error bars in panels A, C, and D indicate 95% CIs. Box plots in panel B were created by the Tukey method; horizontal line indicates median; upper and lower border of the box indicates 75th / 25th percentile; error bars in panel B indicate upper and lower values as calculated by the Tukey method.

Of the 168 participants from the ITT sample, 144 completed the study up to week 6 and 125 completed the 6-month follow-up period. The time course of depression severity (MADRS scores) during the 6-week-treatment period and changes from baseline of MADRS scores at the end of the 6-week-treatment period are shown in Figure 2. There was a mean (SD) reduction of 8.46 (7.08) points in the MADRS score in the minocycline group and of 8.01 (9.07) points in the placebo group after 6 weeks of treatment. The primary end point as assessed by the MMRM analysis, the difference of the least squares means at week 6 for the 2 treatment groups, was not statistically significant (1.46 [95% CI, −1.04 to 3.96]; *P* = .25). Adjustments of MMRM analysis for the variables gender, SSRI, and months of current medication, which showed differences between treatment groups at baseline, did not result in statistically significant differences between treatment groups for the primary end point. Response and remission, as defined by 50% reduction of initial depression severity or reaching a total MADRS score of less than 9 points after week-6 of minocycline treatment, were not statistically significant different between groups as assessed by the odds ratio estimated by the logistic regression model (Table 2). Secondary outcome measures included changes in BDI, CGI-S, HAMD-17, and SCL-90, as well as TMT A and B and CRP levels (Figures 2C and 2D, Table 3; eTable 3, eFigures 1-9 in Supplement 2). The difference between minocycline treatment and placebo of these secondary outcomes was not statistically significant, neither was the difference between baseline and week 6 for the 2 treatment groups as assessed by the mixed model. For exploratory analyses, leukocyte count showed a statistically significant difference between the treatment groups from baseline to week 6 (0.06 [95% CI, 0.00-0.12]; *P* = .047) as assessed by the difference of the least squares means between the treatment groups based on the linear mixed model. The leukocyte count in the minocycline group decreased compared with the count in the placebo group (Table 3). Adherence to trial medication was assessed by means of therapeutic drug monitoring during the trial. Presence of minocycline was observable in all patients randomized to the intervention group. Exploratory end points included measures for anhedonia, sickness behavior, and fear and/or suicidality, based on items of MADRS and HAMD (eTable 2). There were no significant differences between the treatment groups based on the least square differences for the change of these exploratory outcome measures from baseline to week 6 based on linear mixed effects models for repeated measures.

**Table 2.** Response and Remission Rates

Secondary end points	Patients, No. (%)		Odds ratio for placebo vs minocycline (95% CI)	*P* value
	Minocycline	Placebo		
Remission^a^	10 (12.4)	10 (11.5)	0.93 (0.36-2.38)	.88
Response^b^	14 (17.3)	21 (24.1)	1.53 (0.72-3.26)	.27

^a^ Remission was defined as a MARDS score of less than 9 points.

^b^ Response was defined as a 50% reduction of MADRS scores at week 6 to baseline.

**Table 3.** Primary, Secondary, and Exploratory End Points

Primary, secondary and exploratory end points	Mean (SD)								Difference of week 6 and baseline between Placebo and minocycline (95% CI)^a^	*P* value
	Pre-treatment				Post-treatment					
	Patients, No.	Minocycline, mean (SD)	Patients, No.	Placebo, mean (SD)	Patients, No.	Minocycline, mean (SD)	Patients, No.	Placebo, mean (SD)		
MADRS	81	26.4 (4.8)	87	26.6 (5.1)	69	17.6 (7.8)	75	18.7 (9.0)	1.46 (−1.04 to 3.96)	.25
BDI II	78	32.3 (9.0)	87	32.9 (10.4)	66	21.6 (12.0)	72	23.6 (13.2)	1.56 (−2.00to 5.11)	.39
CGI	81	4.8 (0.6)	87	4.9 (0.7)	69	3.9 (1.1)	75	4.0 (1.2)	0.05 (−0.29 to 0.40)	.77
HAMD-6	81	11.4 (2.2)	87	11.4 (2.1)	69	7.4 (3.5)	71	7.6 (6.7)	0.33 (−0.77 to 1.42)	.56
HAMD-17	77	20.0 (3.5)	81	20.3 (3.5)	68	13.1 (5.9)	71	14.2 (6.7)	1.16 (−0.62 to 2.93)	.20
SCL-GSI	81	68.7 (6.7)	87	66.9 (7.0)	66	62.3 (10.7)	72	62.0 (10.1)	1.24 (−1.76 to 4.25)	.42
SCL-PSDI	81	66.3 (4.9)	87	66.1 (6.0)	65	60.2 (8.3)	72	60.2 (8.9)	0.68 (−1.83 to 3.18)	.59
SCL-PST	81	64.4 (6.6)	87	62.2 (6.0)	66	60.5 (10.1)	72	59.5 (8.9)	0.85 (−3.53 to 5.24)	.39
TMT-A	80	34.7 (16.6)	87	35.1 (14.3)	69	29.9 (12.2)	74	30.4 (14.1)	0.56 (−3.81 to 4.93)	.80
TMT-B	79	77.5 (32.7)	85	76.1 (32.3)	69	68.1 (27.6)	72	68.8 (29.1)	1.67 (−7.73 to 11.11)	.73
Log CRP	81	−0.10 (1.6)	86	−0.49 (1.4)	50	0.37 (1.2)	57	0.03 (1.3)	0.24 (−0.04 to 0.53)	.09
Log Leukocytes	76	1.96 (0.31)	79	1.95 (1.85)	66	1.77 (0.29)	69	1.82 (0.28)	0.06 (0.00 to 0.12)	.05

Abbreviations: BDI II, Beck’s depression inventory Version II; CGI, clinical global impression scale; HAMD, Hamilton Depression Rating Scale; MADRS, Montgomery Asberg Depression Rating Scale; SCL-GSI, global severity index; SCL-PSDI, positive symptom distress; SCL-PST, positive symptom total; TMT, trail making test, version A or B.

^a^ The differences week 6 to baseline between the two treatment groups are based on the difference of least square means of linear mixed effects models for repeated measures.

Exploratory subgroup analyses of the primary end point (MADRS score) were conducted for gender and by median-split for baseline CRP levels, severity of depressive symptoms at baseline, body mass index (BMI), and childhood trauma as assessed by the childhood trauma questionnaire (CTQ; eFigures 10-14 in Supplement 2). Neither stratification for gender, nor for CRP levels at baseline or baseline severity of depressive symptoms, BMI nor stratification for type or intensity of childhood trauma had effects on separating minocycline from placebo (eFigures 10-14 in Supplement 2).

Minocycline was well tolerated; discontinuation rates and adverse events were similar in the intervention and placebo group. In total, 85.2% of participants (69 of 81) in the minocycline and 86.2% of participants (75 of 87) in the placebo group completed the 6-week trial. The reasons for discontinuation are summarized in Figure 1. There were a total of 6 severe adverse events within the minocycline group and 7 within the placebo group (eTable 1 in Supplement 2).

## Discussion

To our knowledge, MinoTRD is the largest randomized clinical trial of minocycline in TRD. Major findings included: (1) six weeks of minocycline as adjunct to standard antidepressant medication did not outperform placebo in patients with TRD; and: (2) 200 mg daily of the teracycline was well tolerated over a treatment period of 6 weeks in this sample. The failure of minocycline treatment to reduce depressive symptoms in a naturalistic sample of patients with TRD is a setback for anti-inflammatory treatment strategies in this clinical population, given the suggestive evidence for advantageous effects of minocycline from prior, albeit considerably smaller, trials.^23,24,25,26^

The data from our trial are partly in contrast to previous studies that suggested an antidepressant effect of minocycline.^23,24,25,26,35,36^ However, all RCTs of minocycline in depression were characterized by comparably small sample sizes and highly heterogenous populations of depressed patients.^23,24,25,26,37^ In TRD, Nettis and colleagues^26^ have recently published a study suggesting efficacy of minocycline only in participants with higher baseline levels of CRP, while no treatment effects on the primary outcome were identified. In their study, only patients with at least marginally elevated levels of CRP were included, and the positive findings were observed in a subgroup of only 6 participants with CRP levels at least 3 mg/L. Conversely, our trial was powered to detect clinical differences in patients with TRD, where elevated inflammation may be expected per se, without the need to stratify patients a priori based on absolute values of calibrated paraclinical parameters. Our study therefore included all patients fulfilling the clinical definition of treatment resistance. Inflammation status and numerous other paraclinical parameters were monitored as putative biomarkers throughout the treatment period.

Similar effects were demonstrated for a TNF-α antagonist being more efficient in patients with increased inflammatory markers.^11^ However, based on the hypothesis of chronic, subthreshold inflammation in TRD that may well be present at the level of microglial activation even without measurable traces in the periphery, also patients without significantly elevated peripheral inflammation may have exhibited a minocycline-targetable central inflammation and might thus have responded to minocycline treatment. In a small proof-of-concept trial assessing a potential antidepressant effect of 12 weeks of minocycline in non–treatment-resistant patients, positive changes in secondary outcomes were observed, while primary depression-related outcomes were not improved by adjunct minocycline to treatment as usual.^24^ In contrast, another small study focusing on TRD conducted by Husain and colleagues^25^ demonstrated quite strong effects of minocycline compared with placebo which became evident by and after 8 weeks of treatment. Thus, one explanation for the null result of the MinoTRD trial could be that treatment duration (ie, 6 weeks) was too short to reveal detectable differences. However, a closer look at the data from Husain et al^25^ suggests that their overall effect in the intervention group was due to an almost complete lack of improvement in the placebo group, which is very unusual. In our sample, for instance, the mean (SD) decrease in the MADRS scores was 8.01 (9.07) points for placebo and 8.46 (7.08) points for minocycline, which represents a magnitude of improvement for placebo that is well expected from similar trials in TRD.^33,38^

While prior research had suggested that anti-inflammatory approaches may be more efficient in patients with increased inflammatory markers,^11^ we purposely sought to recruit a naturalistic population of TRD, assuming elevated baseline inflammation as a potentially underlying cause in at least a subgroup of the recruited patients. Interestingly, post hoc stratification for baseline CRP levels did not yield any results supporting a hypothesis of minocycline treatment possibly being more effective in participants exhibiting higher-grade baseline inflammation (eFigures 8 and 12 in Supplement 2).

### Strengths and Limitations

The MinoTRD trial has strengths and limitations. We included inpatients and outpatients and recruited via a broad network of trial-experienced academic centers. The rather wide range of eligibility criteria and the approach to include patients regardless of a priori–determined inflammatory parameters allowed for a naturalistic sample of patients with TRD. From a methodological standpoint, the study was well powered and controlled, thus, adding reliable evidence to the discussion about possible advantages of anti-inflammatory strategies in patients with TRD.

A possible limitation of the MinoTRD trial could be that the chosen treatment duration of 6 weeks may be too short, and longer treatment periods would have been required to yield detectable differences. However, one of the goals of novel treatment strategies is a more rapid onset of action suggesting that a treatment period limited to 6 weeks should be considered advantageous compared with longer treatment regimens for any novel treatment strategy in TRD. Another limitation may be seen in the drug being an antibiotic, possibly limiting a broader use with respect to potential effects on the microbiome, particularly development of antibiotic resistance.

Interestingly, gender distribution was almost equally balanced for male and female participants and thus different from an epidemiologically expected 2:1 ratio for female-to-male genders. One possible explanation for this could be that certain contraceptive measures were required for women participating in the present trial.

Finally, we purposely did not preselect the study population based on increased peripheral inflammation markers, arguing that subthreshold inflammation might generally co-occur in TRD. This may be considered a limitation on the one hand, while including all patients regardless of their individual inflammatory status, on the other hand, may also be considered a strength of the design.

## Conclusions

This randomized clinical trial aimed to assess the antidepressant efficacy of add-on minocycline in a naturalistic clinical population with TRD given the great clinical relevance of this subgroup of patients with depression. Six weeks of 200 mg adjunct minocycline treatment was well tolerated yet failed to reduce depressive symptoms in patients with treatment-refractory MDD compared with placebo. Our results from this large RCT of a pleiotropic anti-inflammatory drug in this difficult-to-treat patient population are of great clinical importance, robustly demonstrating that minocycline add-on treatment does not outperform placebo, not even in those participants with elevated levels of CRP prior to treatment initiation.

A thorough assessment of biomarker profiles, including alterations of various cytokines and plasticity-modulating retinoids over the course of the treatment period promises further options for stratification of subgroups and identification of potential response-predicting biomarker profiles, including potential biomarkers from transcriptome-wide analyses that are presently ongoing.

## Notes

Supplement 1.
Trial Protocol

Supplement 2. **eTable 1.** Adverse Events **eTable 2.** Exploratory Endpoints **eTable 3.** Exploratory Subgroup Analysis **eFigure 1. **CGI **eFigure 2.** Boxplot HAMD-17 **eFigure 3.** BDI **eFigure 4.** SCL-90 **eFigure 5.** TMT-A/B **eFigure 6.** Distribution of Remission by Assignment **eFigure 7.** Distribution of Response by Assignment **eFigure 8.** CRP **eFigure 9.** Leukocytes **eFigure 10.** Gender **eFigure 11.** MADRS at Baseline **eFigure 12.** CRP **eFigure 13.** BMI **eFigure 14.** CTQ

Supplement 3.
Data Sharing Statement
