# Supplementary material for: Effect of Minocycline on Depressive Symptoms in Patients With Treatment-Resistant Depression: A Randomized Clinical Trial
Source: JAMA Netw Open. 2022 Sep 14;5(9):e2230367. doi: 10.1001/jamanetworkopen.2022.30367 (PMC9475381; doi:10.1001/jamanetworkopen.2022.30367)
Supplement: Supplement 1. — Trial Protocol [file jamanetwopen-e2230367-s001.pdf]

A double-blind, placebo-controlled, randomized, multicenter trial to evaluate the efficacy of minocycline augmentation in patients with refractory unipolar depressive disorder

Short name / Acronym: Mino-TRD (OptiMD)

EudraCT number: 2015-001456-29

Trial Protocol

Version 1.7 / Date: 18.05.2018

Sponsor of the clinical trial

Charité - University Medicine Berlin

Head of the clinical trial (LKP)

Prof. Dr. Dipl.-Psych. Isabella Heuser
Clinic for Psychiatry and Psychotherapy
Charité - Campus Benjamin Franklin
Hindenburgdamm 30
12203 Berlin

The following persons agree to the contents of this protocol by their signature and confirm that they are aware of the ICH-GCP Guidelines, the requirements of the AMG and the GCP Regulation and that the clinical trial will be conducted according to these regulations.

Sponsor/Representative

	Prof. Dr. Dipl.-Psych. Isabella Heuser	Berlin, date

Clinical Trial Director/Investigator

	Prof. Dr. Dipl.-Psych. Isabella Heuser	Berlin, date

Translated version of the German trial protocol

The information in this protocol is to be kept strictly confidential. It is intended only for the information of the investigator, his staff, the Ethics Committee, the higher federal authority, the CRO, the PPS and for patient education.

Version History

Clinical Trial Protocol	Protocol Version/ Date	Comment
Initial	1.0, 20-May-2015	Submitted to EC/ CA
Answer conditions EC	1.1, 14-Jul-2016	Answer Submitted to EC
Answer conditions CA/ Amendment #1 for EC	1.2, 02-Sep-2015	Answer Submitted to CA<br>Submitted Amendment #1 to EC
Amendment #2	1.3, 18-Mar-2016	Submitted to EC/ CA
Answer conditions Amendment #2 EC	1.4, 12-May-2016	Answer Submitted to EC<br>Non-substantial Amendment for CA
Amendment #3	1.5 10-Nov-2016	Submitted to EC/ CA
Amendment #4	1.6 21-July-2017	Submitted to EC/ CA
Answer conditions EC	29.11.2017	Answer Submitted to EC
Non-substantial Amendment for EC	20.12.2017	Submitted to EC
Amendment #4	1.7 18-May-2018	

Translated version of the German trial protocol

Table of contents

List of abbreviations 6

1 Synopsis 7

2 Flowchart 18

2.1 Introduction and background 19

2.2 State of knowledge of the investigational drug/therapy under investigation. 24

2.3 Question and justification of the project (rationale) 24

3 Objectives of the clinical trial 25

Secondary endpoints 26

3.1 Study design 26

3.2 Schedule 28

4 Patient selection 28

4.1 Inclusion criteria 28

4.2 Exclusion criteria 28

4.3 Note on contraception 29

5 Treatment plan 30

5.1 Description of the test medication 30

5.1.1 List of side effects and interactions 30

5.1.2 Treatment scheme 33

5.1.3 Storage, issue and return 33

5.1.4 Compliance 33

5.2 Placebo/ comparator medication 34

5.3 Concomitant medication 34

5.4 Emergency measures 34

5.5 Blinding and emergency envelopes 34

5.6 Unblinding (breaking the code) 34

6 Course of studies 35

6.1 Procedure of recruitment / screening 35

6.2 Informed consent procedure 35

6.3 Procedures to avoid simultaneous inclusion in multiple studies. 35

6.4 Enrollment, registration, and randomization (allocation of study medication). 36

6.5 Patient inclusion (enrollment) 37

6.6 Clinical examinations and deviations from standard clinical practice, visit schedule. 37

6.6.2 Pre-screening and screening examination (visit 1) 40

6.6.3 Initial examination (visit 2) 40

Translated version of the German trial protocol

6.6.4 Follow-up examinations (visit 3-7) ... 41
6.6.5 Final examination (visit 8) ... 41
6.6.6 1st follow-up examination (visit 9) ... 42
6.6.7 2. follow-up examination ... 42
6.6.8 Survey of target parameters: ... 42
6.6.9 Laboratory tests / handling of samples ... 43
6.7 Duration of study participation for the individual patient ... 48
7 Risk-benefit assessment ... 48
7.1 Risks, side effects, burdens, advantages and disadvantages for the participant ... 48
8 Termination and further treatment ... 49
8.1 Early study termination of a single patient ... 49
8.2 Premature termination of the entire clinical trial ... 50
8.3 Plan for continuing treatment and medical care after training/graduation ... 50
9 Adverse events ... 51
9.1 Definitions ... 51
9.2 Intensity assessment ... 52
9.3 Assessment of the causal relationship ... 52
9.4 Documentation of AEs and SAEs ... 52
9.5 Reporting of SAEs and suspected serious unexpected adverse reactions (SUSARs) ... 53
9.6 Pregnancies ... 54
9.7 Contact person and person responsible for the reports ... 54
9.8 Data and Safety Monitoring Board ... 54
10 Documentation ... 55
10.1 Patient documentation forms (CRF) ... 55
10.2 Principal investigator folder ... 55
10.3 Documentation of study medication (drug accountability) ... 55
11 Quality management ... 55
11.1 Monitoring of the study process and data quality ... 55
11.1.1 Monitoring ... 55
11.1.2 Audits/ inspections ... 56
12 Data entry and data management ... 56
12.1 Data security ... 57
12.2 Data protection ... 57
12.3 Data quality ... 58
12.4 Data transfer ... 58

Translated version of the German trial protocol

12.5 Generation of the pseudonym ... 59
13 Statistical analysis ... 59
13.1 Case Count Estimate ... 59
13.2 Randomization ... 59
13.3 Statistical evaluation ... 59
13.3.1 Hypotheses ... 59
13.3.2 Definition of the evaluation population ... 60
13.3.3 Evaluation of primary and secondary target parameters ... 60
13.3.4 Safety evaluation ... 61
13.3.5 Potential interim outcomes and endpoints for early dropout. ... 61
14 Reporting ... 61
14.1 Biometric report ... 61
14.2 Final Report ... 61
14.3 Publication ... 61
15 Ethical, legal and administrative aspects ... 61
15.1 Vote of the Ethics Committee (according to AMG § 42 (1) and GCP-V § 7) ... 61
15.2 Approval of the higher federal authority (according to AMG § 42 (2) and GCP-V § 7) ... 62
15.3 Notification to the state authorities (according to AMG § 67) ... 62
15.4 Patient information and consent form ... 62
15.5 Proband insurance ... 62
15.6 Data protection ... 62
15.7 Retention of data and access to data ... 63
16 Bibliography ... 64

Translated version of the German trial protocol

List of abbreviations

AE	Adverse Event = Adverse Event
BDI	Becks Depression Inventory
CGI-S	Clinical Global Impressions-Severity-Scale
CTQ	Childhood Trauma Questionnaire
HAMD-17	Hamilton Depression Rating Scale 17
ITT	Intent to treat Population
MADRS	Montgomery-Åsberg Depression Rating Scale
MGH ATRQ	MassachusettsGeneral Hospital Antidepressant Treatment History Questionnaire.
PANSS	Positive and Negative Syndrome Scale
PBMC	Peripheral blood mononuclear cells
PPP	Per protocol population
SAE	Serious Adverse Event
SCL-90R	Symptom Check List 90R
TMT	Trail Making Test
CNS	Central nervous system

Translated version of the German trial protocol

1 Synopsis

**Study title**	A double-blind, placebo-controlled, randomized, multicenter trial to evaluate the efficacy of minocycline augmentation in patients with refractory unipolar depressive disorder
**Type of project**	Clinical trial according to the AMG of phase II
**Sponsor**	Charité University Medicine Berlin
**Representative of the sponsor and head of the clinical trial**	Prof. Dr. Dipl.-Psych. Isabella Heuser Institute<br>Clinic for Psychiatry and Psychotherapy Charité -<br>Campus Benjamin Franklin Hindenburgdamm 30<br>12203 Berlin<br>Phone +49 30 450 517522<br>Fax +49 30 450 517930<br>Email Isabella.Heuser@charite.de
**Examiner (Berlin Examination Office)**	Prof. Dr. Dipl.-Psych. Isabella Heuser Institute<br>Clinic for Psychiatry and Psychotherapy Charité -<br>Campus Benjamin Franklin Hindenburgdamm 30<br>12203 Berlin<br>Phone +49 30 450 517522<br>Fax +49 30 450 517930<br>Email Isabella.Heuser@charite.de
**Deputy Examiner (Berlin Examination Office)**	Dr. Julian Hellmann-Regen<br>Institute Clinic for Psychiatry and Psychotherapy<br>Charité - Campus Benjamin Franklin<br>Hindenburgdamm 30<br>12203 Berlin<br>Phone +49 30 450 517 654<br>Fax +49 30 450 517 947<br>Email Julian.Hellmann@charite.de
**Hypothesis**	Minocycline, a pleiotropic, CNS-transportable tetracycline, reduces the symptoms of depressive patients as part of an adjunctive therapy. The reasons for this include an anti-inflammatory and neuroprotective effect.
**Research Question**	With a prevalence of 8.1% in Germany, unipolar depression is one of the most common mental disorders. Standard therapy with conventional antidepressants does not lead to a sufficient response in a high number of patients, accompanied by a satisfactory reduction of depressive symptoms. In order to achieve an improvement here, new substances in the pharmacotherapy of depression are urgently needed. Minocycline is a CNS-permeable tetracycline with pleiotropic activity. Because of its antibiotic effects, minocycline is useful in many indications, such as infections of the<br>upper respiratory tract, approved and is characterized by a good

Translated version of the German trial protocol

	tolerability. In the context of its pleiotropic efficacy, neuroprotective as well as anti-inflammatory effects, which are not due to the antibiotic effect of minocycline, have been demonstrated. Depression has a complex pathogenesis, which in addition to Components of neurotransmitter homeostasis, is also influenced by neuroinflammatory and neurotrophic mechanisms. Furthermore, studies show a detrimental influence of neuroinflammatory processes on neurotransmitter homeostasis, which are targets for antidepressants. This could explain the non-response to conventional antidepressants. An increase in inflammatory markers such as CRP, TNF-α, and IL-6 is known to occur in depressed patients. In this light, giving minocycline as an adjunctive therapy to antidepressants is a very promising approach. In the treatment of schizophrenic patients, a reduction of depressive symptoms could be achieved. In patients with a depressive disorder with psychotic symptomatology, a treatment trial with minocycline has so far only been carried out in the context of an open trial by achieving a significant improvement in psychotic symptomatology. For an evidence-based answer to the question of whether minocycline is effective as an adjunctive therapy in major depression, the conduct of a randomized controlled trial is urgently needed.
**Trial medication / treatment strategy**	Minocycline 50mg each 2 x morning and evening (200mg/day).
**Comparative medication**	Placebo
**Study design**	Prospective double-blind, placebo-controlled, two-arm, randomized, multicenter trial; the investigational drug or placebo drug is used as an adjunctive medication to standard therapy consisting of conventional antidepressant medication.
**Schedule**	Start of recruitment (first patient first visit, FPFV) >12/2015< End of recruitment >06/2020<<br>End of clinical trial (last patient last visit, LPLV) >06/2020< Duration of individual study participation: 6 weeks.<br>Period follow up: >02/2016-12/2020<
**Total number of patients**	160
**Study population**	Screening population: 300<br>Number of patients to be included in the study: 160 Total number: 160 (80 per study arm).<br>Number drop outs: 32<br>Number of patients to be evaluated: 160 in ITT evaluation,<br>of which 160-32= 128 (64 patients in the verum arm and 64 patients in the placebo arm) with complete follow-up.

Translated version of the German trial protocol

**Inclusion criteria**	• patient education and written consent (according to AMG §40 (1) 3b)<br>• Age 18 - 75<br>• BMI: 18 - 40 inclusive<br>• negative pregnancy test<br>• adequate contraception (pearl index < 1) (see also section 4.3)<br>• Meeting the criteria for an episode of major depression according to DSM 5<br>• Hamilton Depression Rating Scale (HAMD-17) score of at least 16<br>• Clinical Global Impression Scale (CGI-S) score of at least 4<br>• Pharmacotherapy with an antidepressant approved for the treatment of depression in a sufficient dosage according to the professional information for at least 6 weeks at the time of screening without a sufficient response according to the criteria of the MGH ATRQ. Simultaneous treatment with other antidepressants or with quetiapine or aripiprazole in any dosage (e.g. for sleep induction) is possible. All antidepressants must have been taken for at least 6 weeks prior to screening, of which at least 2 weeks must have been in constant dose must have been administered.
**Exclusion criteria**	• Presence of a neurodegenerative disease<br>• Presence of a neurological disease in the context of which the depressive symptomatology occurred<br>• Presence of any severe, unstable somatic disease, including chronic inflammatory diseases such as rheumatologic diseases or chronic inflammatory bowel disease.<br>• Depressive symptomatology is better explained by the presence of a possibly comorbid other psychiatric disorder, especially a personality disorder.<br>• Improvement of more than 50% in HAMD-17 value during the last 14 days.<br>• Pregnancy and breastfeeding women<br>• Substance or alcohol abuse within the past 6 months or a positive urine drug screen (medication with benzodiazepines allowed, as well as nicotine use)<br>• Thyroid dysfunction (if not euthyroid substituted), severe liver or kidney dysfunction.<br>• Known autoimmune disease (lifetime prevalence) other than euthyroid Hashimoto's thyroiditis<br>• Clinically relevant laboratory abnormalities<br>• Current medication with anti-inflammatory substances (NSAIDs, corticosteroids)<br>• Current medication with retinoids (retinol, retinoic acid, or rentinoid receptor agonists).

Translated version of the German trial protocol

	• Any past adverse event associated with previous tetracycline use, including any hypersensitivity reaction to tetracyclines and tetracycline intolerance<br>• Any medication that leads to known interactions with minocycline. Should such medication become necessary during the study period, this will result in discontinuation of minocycline.<br>• Current medication with barbiturates and other anticonvulsant drugs (e.g., carbamazepine, diphenylhydantoin, and primidone).<br>• Current medication with beta-lactam antibiotics, such as penicillins or cefalosporins.<br>• Current medication with isotretinoin or medication with isoretionin in the last 4 weeks.<br>• Current medication with theophylline<br>• Current medication with sulfonylurea antidiabetics and anticoagulants of the coumarin type<br>• Current medication with ciclosporin A<br>• Current medication with methotrexate<br>• Performance of methoxyflurane anesthesia or medication with other substances that can damage the kidney.<br>• Lack of willingness to store and share pseudonymized disease data in the context of clinical trials<br>• Concurrent use of other psychotropic substances in addition to standard antidepressant medication. Lorazepam (max. 4 mg/day) or Z-hypnotics (zolpidem (max. 10 mg/day), zopiclone (max. 7.5 mg/day)) are permitted exclusively for the treatment of acute agitation/anxiety or severe sleep disorders.<br>• Participation in a clinical trial according to the AMG within the last 8 weeks<br>• Placement in an institution by court or official order<br>• Patients who are dependent on the investigator, sponsor, or trial site<br>• Acute suicidality, suicide plans or attempts in the current episode of illness, or suicide attempt within the past 5 years
**Documentation times**	Prescreening:<br>Selection of possible suitable patients<br>Patient information and consent to the study.<br><br>Assessment of suicidality (by means of item 10 "Suicidal thoughts" in the MADRS score.<br><br>Screening:<br>Up to 1 week before study inclusion (verification of enrollment and Exclusion criteria, history taking, performance HAMD-17,

Translated version of the German trial protocol

	CGI-S, BDI, MGH ATRQ, physical examination, vital signs, weight, height, safety laboratory, pregnancy test in women of childbearing age, urine drug screening, ascertainment of concomitant treatment, ascertainment of suicidality (using item 10 "suicidal ideation" in the MADRS score).<br><br>Regular Study Visits:<br>Time point of study entry (day 0, week 1), week 2-6, time point of study end (week 7).<br><br>Control examinations:<br>6 weeks after the end of the study,<br>6 months after end of study by phone
**End Points**	• primary end points:<br>Change in MADRS score from start time (week 1) to week 7, weekly examination.<br>Time point of determination of the primary outcome parameter: weeks 1-6, 7, 6 weeks, and 6 months after study end.<br><br>• secondary and exploratory end points:<br>Remission; remission is defined as reduction of the MADRS value to less than 9<br>Response to therapy (response is defined as a reduction in MADRS of more than 50% compared to week 1).<br>• Scores of CGI-S, BDI, HAMD-17 and SCL-90R, TMT A and B (determination times CGI-S, BDI, HAMD-17: each at the time of screening, during the study in each week 1-6 and at the end of the study (week 7) as well as each 6 weeks and 6 months after the end of the study (not BDI); TMT A and B, SCL 90R: each at the time of screening, during the study in week 4 (only SCL-90R) and at the end of the study (week 7) as well as in the 1st follow-up (6 weeks after the end of the study). Follow-up (6 weeks after end of study).<br>• Expression levels in peripheral blood mononuclear cells (PBMCs) and serum concentrations of various cytokines, cell type-specific markers, neurotrophic factors, and factors involved in inflammatory processes and cell metabolism (see Section 6).<br>Time of determination per week 1, 4 (non-PBMCs) and week 7 (after end of study).<br>• Genotype determination of therapy-influencing genetic variants (e.g., rs2032583, rs2235015, rs2235040, rs1045642, rs2032582, rs1128503 in the ABCB1 gene) (week 1).
**Safety Measures**	Pregnancy test in women of childbearing age. Vital signs, weight, laboratory parameters and physical examination at screening, baseline, end of study and weekly during the study phase. Control of vital signs, and weight and physical examination in addition 2 weeks after study end. Adverse event survey

Translated version of the German trial protocol

	Study start weekly and at the end of the study, also 6 weeks and 6 months after the end of the study.
**Termination criteria**	for the (individual) study participant:<br>• Occurrence of serious adverse events if they are likely to be related to the study drug<br>• Paraclinical parameters assessed by the investigator as clinically relevant and collected in the safety laboratory, which may indicate disturbances of liver and kidney and pancreas function, changes in the blood count as well as electrolyte balance<br>• Any sign of minocycline-related allergic reaction.<br>• Any evidence of liver or kidney toxicity during regularly scheduled physical examinations<br>• Occurrence of clinical or paraclinical evidence of other minocycline-associated adverse reactions, such as dental or skin discoloration, severe headache, visual disturbances, pericarditis, nephritis, hepatitis, pancreatitis, systemic lupus erythematosus, severe diarrhea with evidence of pseudomembranous colitis<br>• Withdrawal of consent,<br>• Occurrence of new concomitant diseases that make further study participation not possible/ not reasonable;<br>• Violation of inclusion criteria,<br>• Subsequent occurrence of an exclusion criterion<br>• Occurrence of acute suicidal tendencies: Assessment by means of item 10 "Suicidal ideation" in MADRS score, discontinuation if expression > 4 points.<br><br>for the entire study:<br>• Decision of the study management in case of unacceptable risks and toxicities under benefit-risk consideration<br>• new (scientific) findings during the duration of the clinical trial that may jeopardize the safety of the trial participants (positive benefit-risk assessment no longer given)<br>• Request of the sponsor (if applicable, the head of the trial) or the responsible higher federal authority<br>• safety reasons, e.g. on the advice of the independent Data and Safety Monitoring Board (DSMB) deemed it necessary to terminate the study;<br>• Lack of recruitment
**Statistical evaluation**	Efficacy: The primary endpoint of change in MADRS sum scores from week 1 to week 7 will be evaluated by a mixed model repeated measures (MMRM) linear model.

Translated version of the German trial protocol

	Study population: the primary evaluation population is the intention-to-treat population.<br><br>Safety: The frequencies (percentages) of adverse AEs and SAEs, as well as the occurrence and severity of treatment-related adverse events (ARs), are reported for the treatment groups. Kaplan-Meyer curves of times from initiation of therapy to occurrence of the event will be generated for selected events. In addition, descriptive statistics will be calculated for clinically significant changes in vital signs, weight, etc.<br><br>Secondary outcome parameters:<br>The evaluation of the other scales such as CGI-S, BDI, SCL-90R and HAMD-17 as well as the TMT A and B will be performed analogously to the evaluation described above for MADRS. The secondary endpoints remission and response to treatment will be evaluated using logistic regressions.
**Pharmacological-toxicological testing**	The substance under investigation, minocycline, is a widely used representative of the established tetracycline antibiotics. Minocycline is used worldwide as an antibiotic and as an anti-inflammatory substance, both systemically and topically, e.g. in anti-acne therapy. The patent protection for minocycline has expired and numerous generic preparations already exist.<br><br>Due to frequently observed pleiotropic, in particular anti-inflammatory and neuroprotective effects in various animal and cell culture models, minocycline is increasingly being investigated in the neuropsychiatric, and in particular also in the clinical context. Thus, controlled clinical studies have already demonstrated that minocycline leads to an improvement of negative symptoms in patients suffering from schizophrenia.<br><br>Interestingly, inflammatory processes, including pathological activation of the brain's own macrophages (microglial cells), appear to play an important role in the pathogenesis of both negative symptoms and depression. Preclinical studies have also shown that chronic microglial activation leads to increased degradation of retinoic acids and thus to the deprivation of important endogenous neuroprotective factors.<br><br>Especially for refractory courses, an association with elevated (neuro)-inflammatory parameters is known and recently efficacy of specific anti-inflammatory interventions in this patient group could be demonstrated.<br><br>Preclinical data convincingly demonstrate that inhibition of microglial cell activation is a key target of minocycline.
**Potential risks,**	**Potential risks:**

Translated version of the German trial protocol

	No indication-specific risks are expected

Translated version of the German trial protocol

**Side effects,**	<u>**Possible side effects**</u> (the following information is taken from the expert information):<br><br>**Gastrointestinal disorders:** Heartburn, gastric pressure, vomiting, meteorism, fatty stools, and mild diarrhea. Very rarely oral and pharyngeal mucositis, hoarseness and difficulty swallowing, pseudomembranous enterocolitis.<br><br>**Nervous system:** dizziness, nausea, ataxia and fatigue.<br>Rare: reversible pseudotumor cerebri with headache, nausea, vomiting, and possibly papilledema. In addition, paresthesias may occur.<br><br>**Sensory organs:** Very rarely temporary myopia. Pigment deposits in the eye (cornea, sclera and retina) may occur.<br><br>Rarely, tinnitus and hearing deterioration may occur.<br><br>**Skin and skin appendages:**<br>Occasional: Allergic skin reactions.<br>Under sun exposure: phototoxic reactions with erythema, skin edema, blistering and occasionally nail detachment and discoloration. In case of preceding inflammatory skin changes at long-term therapy blue-gray hyperpigmentation possible.<br>After prolonged high-dose therapy very rarely blackish discoloration of the nails. In addition, a so-called "black hair tongue" may occur.<br><br>**Blood and blood cells:** rare: reversible leukopenias, thrombopenias, anemias, leukocytoses, agranulocytoses, eosinophilia, atypical lymphocytes, and toxic granulations of granulocytes.<br><br>**Liver and bile ducts, pancreas, kidneys:** immunologically caused hepatitis. Non-immunological hepatocellular damage and renal damage especially with long-term therapy. In case of overdoses: Liver and kidney damage, pancreatitis.<br><br>**Hypersensitivity symptoms:**<br>Allergic reactions such as generalized exanthema, urticaria, erythema and skin itching are common. With unknown frequency, Quincke's syndrome, erythema exsudativum multiforme, toxic epidermal necrolysis (Lyell's syndrome (or, in a milder form, Stevens-Johnson syndrome)), fixed drug exanthema of the genitals and other body regions, and serum sickness-like reaction with fever, headache, and joint pain may also occur. Pericarditis and exacerbations of systemic lupus erythematosus have been reported. Drug-induced lupus erythematosus may occur.<br>Hypersensitivity syndrome (DRESS, fatal in some cases) may occur, manifested by skin reactions (such as rash or exfoliative dermatitis, eosinophilia, and one or more of the

Translated version of the German trial protocol

	following symptoms: Liver inflammation, inflammation of the lungs, kidney inflammation, myocarditis or pericarditis, fever and lymphadenopathy.<br><br>Clinically, joint symptoms (polyarthritis and polyarthralgia) with negative rheumatoid factor, fever, fatigue, exanthema, and lymphadenopathy have been described. Eosinophilic pulmonary infiltrates possible.<br>Lab:<br>elevated ESR, eosinophilia, elevated ANA titer. Severe acute hypersensitivity symptoms are possible with facial edema, tongue swelling, internal laryngeal swelling with constriction of the airways, cardiac tachycardia, dyspnea (shortness of breath), drop in blood pressure up to threatening shock.<br>There is complete cross-allergy within the tetracycline group.<br><br>**Other:** Candida colonization of the skin or mucous membranes (especially the genital tract and the mucous membranes of the mouth and intestines) by selection with symptoms such as inflammation of the mouth and pharyngeal mucosa (glossitis, stomatitis), acute inflammation of the external genital organs and the vagina in women (vulvovaginitis) and pruritus ani.<br><br>Medication with minocycline may cause an exacerbation of myasthenia gravis.<br><br>Children under 8 years of age: occasional irreversible tooth discoloration and enamel damage, reversible bone growth retardation.<br><br>Adults: after prolonged high-dose therapy with minocycline, occasional blackish discoloration of teeth, nails, and bones and of the thyroid gland. In case of preceding inflammatory skin lesions At long-term therapy, blue-gray hyperpigmentation possible.
**Contraindications,**	<u>**Contraindications:**</u><br>Known hypersensitivity to minocycline, other tetracyclines or other ingredients, and severe liver dysfunction.<br>Do not give to children under 8 years of age.<br>Do not administer during pregnancy and lactation.
**Measures to be taken in case of possible incidents**	<u>**Measures to be taken in case of any adverse events:**</u> In case of allergic reactions and drug-induced lupus erythematosus, discontinue medication immediately.<br>In case of severe acute hypersensitivity symptoms, immediate medical attention is required.
**Risk-benefit assessment**	<u>Known Risks (Verum):</u><br>Minocycline is a long-tested and proven drug that is approved for other indications under the German Medicines Act. Side effects and contraindications are known, participants are informed about these

Translated version of the German trial protocol

explained in detail. As with any substance, new, previously unknown side effects may occur with the use of minocycline. Indication-specific risks in the treatment of depressed patients are not expected.

Residual risk (Verum):
The risks of the Verum are explained in detail in Chapters 5.1 and 7. Against the background of the risks presented, which remain as a residual risk, and against the background of the expected benefit for medical science, the residual risk is justifiable from a medical point of view.

Known risks (placebo):
The placebo has the same composition as the verum, but without the active substance. Pharmacological effects or side effects are not assumed.

Individual benefit to subjects:
If the study hypothesis is confirmed, minocycline is expected to lead to a significant reduction in depressive symptoms up to remission of the disease. Since patients classified as treatment-resistant up to that point are being treated in particular, an individual benefit may also be expected if the participant receives the verum.

Benefit to the general public:
The results obtained from the study will provide valuable insights into the pathomechanism of depression and in particular a possible involvement of neuroinflammatory processes as a pharmacologically modulable target in treatment-resistant depression. This alone may enable the targeted development of new antidepressant therapeutic options for patients previously classified as treatment-resistant. In the short and medium term, the results may represent an important milestone on the way to a clinical approval of minocycline in the indication of augmentation of antidepressant treatment.

Result of the risk-benefit assessment:
Study participation is associated with at most a minor additional patient burden.
In addition to the considerable general gain in knowledge, there is a possible benefit for the individual patient. Against the background of the generally low risk of the drug minocycline, which is used worldwide in its antibiotic indication and has been established and well tolerated for decades, the offer of study participation is well justifiable from a medical point of view.

Translated version of the German trial protocol

2 Flowchart

Pre-screening:

1) Selection of suitable patients
2) Informed consent
3) Survey of current medication/possibly therapy optimization

N=160 to
recruiting patients
80 Pat. placebo
80 Pat. Verum

Screening:

- Review of the inclusion and exclusion criteria
- Medical history collection,
- - Symptom survey by questionnaires

If inclusion criteria are met and exclusion criteria are not present:

- Randomization / study inclusion

Baseline examination prior to the start of the study:

- Physical examination, laboratory control with collection of specific values,
- Laboratory control
  symptom collection via questionnaires, recording of adverse events
- Issue of study medication (minocycline or placebo 50 mg as adjunctive therapy, target dose: 100-0-100 mg.

6 week study period:

- Drug therapy
- Weekly visit appointments with physical examination, laboratory control, symptom survey via questionnaires, recording of adverse events

Final examination after the end of the study (week 7):

- Termination of drug therapy
- Physical examination, laboratory control and collection of specific values, symptom survey by questionnaires, recording of adverse events

Follow-Up Investigations:

- 6 weeks after end of study with physical examination, symptom survey by questionnaires, recording of adverse events.
- 6 months after end of study: Telephone contact with symptom survey via questionnaires, recording of adverse events.

continuation of *standard antidepressant therapy.*

Translated version of the German trial protocol

2. Introduction

2.1 Introduction and background

Depression is one of the most prevalent mental illnesses globally and accounts for a large proportion of the global burden of disease [1]. In Germany, depressive disorders form one of the most common mental illnesses with a prevalence of 8.2%. The lifetime prevalence is 11.6% [2]. Despite enormous efforts in recent decades, standard therapy with conventional antidepressants does not lead to a sufficient response in a relevant number of patients, accompanied by a satisfactory reduction of depressive symptoms. Only 50% of patients respond to initial antidepressant medication. Remission is achieved in only 15-40% of cases even after multiple changes and adjustments in treatment regimens [3]. Treatment refractory depression is defined as a failure to achieve remission. This is associated with multiple negative outcome parameters such as significantly increased risk of relapse, significantly increased number of somatic symptoms, and impaired social skills [4]. Treatment resistance, associated with a high individual and social burden, thus remains one of the most important challenges in depression research.

Non-response, treatment resistance, and chronicity of psychopathology are therefore massive problems in clinical depression treatment. Finding new therapeutic options that specifically help these patients with refractory depressive symptoms is therefore of utmost relevance from both an individual and a societal perspective. In order to achieve an improvement here, new substances in the pharmacotherapy of depression are urgently needed.

Inflammatory pathogenesis of depression

The pathogenesis of depression is of higher complexity than previously thought. Specifically, the monoamine hypothesis, based on which depression is attributed to a reduction in activity of cerebral serotonergic and noradrenergic synapses, is insufficient on its own. Clinical and preclinical evidence implicating neuro-inflammatory and neurotrophic mechanisms as one of the central neurobiological bases of depressive pathogenesis is accumulating [5, 6].

Thus, a significant increase in inflammatory markers has been found in depression [6-8]. Studies show that increased inflammatory levels are particularly associated with poorer response to standard antidepressant medication [9, 10]. In this context, microglial cells undoubtedly play a particularly important role. Microglial cells are the brain's own immune cells (macrophages) and regularly involved in neuroinflammatory processes of any kind. Interestingly, an involvement of this cell type in the pathogenesis of depressive disorders is also discussed [11]. For example, a recently published controlled study demonstrated an increase in activated microglial cells in depressed patients compared to healthy individuals using an imaging technique [12]. Furthermore, neuropathological studies in suicidal patients postmortem showed an increased number of activated microglial cells in the depression-associated area of the dorsal-anterior cingulate cortex [13]. The neuro-inflammatory component associated with marked microglial activation appears to represent a central mechanism in the pathogenesis of depression. It thus represents a promising target for new drug therapies.

Minocycline - anti-inflammatory and neuroprotective effect.

Minocycline is a CNS-trafficking tetracycline with pleiotropic activity. Because of its antibiotic effects, minocycline is used for many infectious indications, such as the treatment of

Translated version of the German trial protocol

upper respiratory tract infections, and is characterized by good tolerability. Due to frequently observed pleiotropic, especially anti-inflammatory and neuroprotective effects in various animal and cell culture models, minocycline is increasingly being investigated in neuropsychiatric issues, and recently also increasingly in the clinical context [14-17].

Preclinical data convincingly demonstrate that inhibition of microglial cell activation is a key target of minocycline [18-22]. In our own preclinical studies on the effects of pathological activation of the brain's own macrophages (microglial cells), it has recently been shown that chronic microglial activation leads to increased degradation of retinoic acid, and thus to the deprivation of important endogenous neuroprotective factors [23]. These changes may represent part of the molecular basis of destructive effects of chronic activation of microglial cells on surrounding neuronal structures and may be excellently disrupted by minocycline. In this context, a direct effect of minocycline on t h e endogenous retinoid homeostasis in human cell lines was recently demonstrated and discussed as a possible mechanism of minocycline action [23]. Preliminary, as yet unpublished data show that this mechanism also seems to apply to a marked extent to murine brain tissue and possibly even the pleiotropic effects of minocycline in dermatological application could be mediated via the (neuroprotective) retinoid system [23].

Minocycline leads to reduction of depressive behaviors in animal models The analysis of mental disorders in animal models is challenging due to the complexity of the disorders. There are now accepted models of depressive behavior in mice and rats that measure response to antidepressant medication with good reliability. Two well-known tests are the forced swim test (FST) and the tail suspension test (TST). In both tests, immobility is scored as a depressive behavior, and a reduction in it is scored as a response to medication. Animal models of depression in rats include rats with learned helplessness, which show typical depressive behaviors such a s weight loss, less activity, and reduced libido [24]. An antidepressant effect of minocycline was demonstrated for the first time in animal models. Molina- Hernández et al. treated male Wistar rats with minocycline. The treatment resulted in reduced immobility (presented in behaviors per 5-second period: control: 32.63±3.8, minocycline 60mg/kg: 14.0±2.4*) and increased climbing (control: 14.5±3.7, minocycline 60mg/kg: 35.6±3.6*) in the forced swimming test. Furthermore, minocycline enhanced the antidepressant efficacy of the tricyclic antidepressant desipramine (shown each for the combination treatment minocycline 50mg/kg and desipramine 5mg/kg, each control vs. treatment: Immobility: 34.5±2.5 vs. 12.5±3.6, Swimming 10.8±3.5 vs. 11.2±4.2, Climbing: 14.7±3.2 vs. 36.3±3.5*) **[25]**. Bilateral intraventricular infusion of minocycline, also in male Wistar rats, reduced depressive behavior in the swimming test (for 1µg minocycline/ventricular side, shown in behavioral points per 5 sec period: immobility significantly reduced from 32.6±3.1 to 11.2±3.6; swimming behavior remained relatively the same from 12.7±3.5 to 11.5±3.9; climbing behavior significantly increased from 14.7±3.2 to 37.3±3.4)[26]. Minocycline was also infused into the cerebral ventricle of rats with learned helplessness. This also resulted in pronounced antidepressant effects such as reduced latency in the conditioned avoidance test (for 160µg/ ventricular side; failure to escape: F= 4.052*, latency: F=3.861*) [27]. In a mouse model of inflammation-associated depression (treatment with the proinflammatory cytokine interferon-α), additional administration of minocycline (50mg/kg) also resulted in a significant reduction (p<0.01) in interferon-α-induced depressive behavior as measured by immobility in the forced swim test, as well as in the tail suspension test[28]. Furthermore, it was demonstrated in the mouse model that lipopolysaccharide-induced depressive behaviors were significantly reduced by minocycline administration (50mg/kg) (p<0.01). In addition, minocycline exposure led to a decrease in LPS-.

Translated version of the German trial protocol

induced expression of inflammatory cytokines (TNF-α, IL-1β and IFN-γ; $p<0.01$) [29]. In conclusion, the antidepressant effect of minocycline has already been convincingly demonstrated in animal models.

Minocycline reduces negative symptomatology in schizophrenic patients.
The first controlled human studies have already demonstrated that minocycline leads to an improvement in (depression-like) negative symptoms in patients suffering from schizophrenia. The clinical picture of schizophrenia, especially the occurrence of negative symptomatology, is characterized by increased inflammatory parameters, as is depression [30]. Negative symptomatology in schizophrenia includes symptoms such as anhedonia, listlessness, decreased psychomotor function, and cognitive impairment, which are very similar to those of depression. Chaudhry et al. found a significant reduction in negative symptoms after minocycline in a study successfully completed with 94 patients (mean improvement in negative symptoms according to the Positive and Negative Syndrome Scale (PANSS) in the minocycline group: 9.2, placebo group: 4.7; $p<0.001$). Patients received 200 mg minocycline daily for 8 weeks as an augmentation to standard therapy compared to the placebo-controlled comparison group, and the observation period was 1 year [31]. Another controlled clinical trial with 70 patients suffering from schizophrenia showed a comparable result. The observation period was 6 months and patients received 200mg minocycline/day or placebo as augmentation to therapy with the atypical antipsychotics for a period of 22 weeks. In addition to good tolerability of minocycline, negative symptomatology as measured by PANSS was significantly lower in the minocycline group (17.10±5.91) than in the placebo group (20.32±6.53; $p<0.001$) [32]. Furthermore, a study of augmentation of risperidone with 100 mg minocycline per day in the first week and 200 mg minocycline daily in the following 7 weeks showed that the treatment group was the strongest predictor of change in negative symptomatology (β=-0.94, t=-10.59, $P<0.001$). Thirty-eight of 40 patients suffering from schizophrenia completed the study, and the side effect profiles between the verum and placebo groups did not differ significantly [33]. An eleven-week augmentation with 200 mg minocycline daily to the atypical antipsychotic clozapine resulted in a significant reduction in depressive symptoms in a study of 52 refractory schizophrenia patients compared to the placebo-controlled comparison group (assessed using the Brief Psychiatric Rating Scale (BPRS), reduction in anxiety/depression factor, Mixed Model Treatment Difference Estimates: -0.9±0.4, p=0,03) [34]. A 2014 meta-analysis on minocycline augmentation in schizophrenia, analyzing data from a total of 330 patients, concluded that minocycline as an add-on therapy in schizophrenia resulted in a significant reduction in negative symptoms (assessed using the PANSS negative Scale, Standardized Mean Difference (SMD) =-0.86) and was characterized by good tolerability. Significant differences between the placebo and minocycline groups studied in terms of all-cause study termination, study termination due to ineffectiveness, and study termination due to adverse events and study termination due to death were not observed. The minocycline groups even showed a lower mean incidence of extrapyramidal motor side effects (SMD =-0.32) [35]. The efficacy of minocycline against negative symptomatology in schizophrenia, which closely resembles the symptomatology of depression, has already been demonstrated in controlled human studies, as shown.

Antidepressant effect of minocycline in an open-label study with depressed patients.
In depressed patients, the antidepressant effect of minocycline has been demonstrated only in an open-label human study. 25 patients with psychotic depression received an augmentation with 150 mg minocycline/day over a period of 6 weeks to their antidepressant therapy with a selective serotonin reuptake inhibitor. A significant reduction of depressive symptoms after minocycline augmentation was shown (assessed by the Hamilton Depression Rating Scale-21; baseline value: 40.4±2.5),

Translated version of the German trial protocol

End of study: 6.7±1.9; p<0.008). No serious adverse effects from minocycline occurred during the study, and safety laboratory values remained within the reference range. Mild and transient headache occurred as an adverse side effect in 2 cases. Study discontinuation due to adverse effects did not occur [36]. Currently, another open-label study is investigating the combination of minocycline and aspirin in the treatment of the depressive phase in bipolar disorder [37]. Accordingly, initial results indicate an antidepressant effect of minocycline. More controlled studies are needed to systematically evaluate the potential effect of minocycline in depression.

The planned study is based on the hypothesis, supported by preclinical data, of the anti-inflammatory effect of minocycline and the associated antidepressant effect, particularly in treatment-refractory depression. The preclinical results listed demonstrate an antidepressant effect of minocycline in animal models, and initial clinical data also indicate a potential antidepressant effect of minocycline.

Planned studies to evaluate minocycline in psychiatric disorders.
A query of the clinical trials database "clinicaltrials.gov" was performed during the planning phase of the study on 04.03.2014. Minocycline was investigated in the neuropsychiatric context in a total of three controlled clinical trials at that time: In addition to a study on the efficacy of minocycline in bipolar affective disorder in combination with aspirin (NCT01429272), in Angelman syndrome (NCT01531582), and regarding remission-maintaining effects in depressed patients after ketamine intervention (NCT01809340) (clinicaltrials.gov; 04/03/2014). A new search on 03/13/2015 additionally reveals several recent studies examining minocycline in the context of depressive symptomatology: Two studies with a minocycline treatment trial in bipolar disorder (NCT01514422, NCT01403662) and one open-label study with minocycline administration in unipolar depression (NCT01574742) are listed. In addition, a study of minocyclin in depressive disorder with an outcome focus of imaging for microglial activation (NCT02362529) and an open-label study of minocycline medication in geriatric depression (NCT01659320). A controlled trial is investigating the effect of minocycline as add-on therapy to conventional therapy for refractory depressive symptoms (NCT02263872).

In the online database "Medline" for medical literature, there are no references to planned or previously conducted controlled clinical studies on the antidepressant efficacy of minocycline in treatment-resistant depression. A planned controlled trial aims to use minocycline as an add-on therapy for depression. The target population is patients with major depression; treatment resistance is not mandatory [38].

The planned study is aimed at patients with a diagnosed depressive disorder in whom conventional antidepressant therapy has led neither to a significant reduction in symptoms nor to remission. Patients will continue to receive their regular standard antidepressant therapy throughout the duration of the study.

Guideline recommendations for augmentation therapy in depression.
In Germany, guidelines have the function of recommendations for action. The current version of the S3 guideline/national health care guideline (attached) was prepared in 2009.
5.version of this edition before.

If there is no response to antidepressant monotherapy, the National Health Care Guideline provides the following option for medication augmentation:

"3-25: An attempt at augmentation with lithium should be considered by the experienced clinician in patients whose depression has not responded to antidepressants." In the presence of treatment-resistant depression, augmentation with lithium results in a response rate of 45% compared with 18% in the placebo group, according to a meta-analysis [39].

Translated version of the German trial protocol

According to another meta-analysis, the number of patients needed to treat to obtain another responder in refractory depression is 3.7 [40]. These data indicate that lithium augmentation is a good option for pharmacotherapy in treatment-resistant depression, but that more than half of patients continue to fail to respond. Furthermore, lithium therapy is associated with a significant number of side effects and is associated with complex serum level measurements, especially at the beginning of therapy, due to the high toxicity of the drug, which is associated with a very low therapeutic range in terms of serum concentration (for the need of further information, the professional information of lithium is attached). Therefore, the guidelines provide the following restriction on lithium augmentation:
"Disadvantages consist in the additional side effects and interactions of lithium [...] (especially cardiovascular). Lithium augmentation should be reserved for trained or experienced physicians [...]".Despite the possibility of augmentation with lithium, a high demand for further, effective augmentation procedures is therefore to be assumed due to the 50% chance of not responding to lithium augmentation. This is especially true since lithium is associated with high toxicity and consequent close serum level monitoring, as well as a variety of side effects and contraindications, that it is only suitable for a specific patient clientele with high compliance. The augmentation with lithium is a meanwhile often questioned procedure, which is hardly used in psychiatric-clinical practice for therapy-refractory depressive patients due to various side effects.

Augmentation of antidepressants using neuroleptics or other medications is explicitly not recommended in the depression guidelines: "3-28: Augmentation of antidepressants using carbamazepine, lamotrigine, pindolol, valproate, dopamine agonists, psychostimulants, thyroid or other hormones is not recommended as a routine use in treatment-resistant depression." The rationale is as follows: "Antipsychotics are indicated only in delusional depression [...] because of the side effect profile (risk of tardive dyskinesia, weight gain, diabetes mellitus, etc.) and insufficient evidence of efficacy. This also applies to conventional depot antipsychotics (e.g., fluspirilene, haloperidol decanoate), which carry a particular risk of tardive dyskinesia [...]".

Overall, it should be noted that the recommendations from the S3 guideline on unipolar depression are from a relatively old edition that is currently being updated. Implementation of the recommendation is by no means mandatory. In particular, the procedure of lithium augmentation mentioned in the guideline is a procedure that has been widely questioned by experts and is hardly used in psychiatric clinical practice for depressive patients who are refractory to therapy due to various side effects. The participants of the Mino-TRD study are therefore not deprived of any standardized therapy that is regularly applied in practice. Of course, in case of a non-response to minocycline, patients will be offered further treatment according to the current state of knowledge.

In summary, the use of minocycline in depressed patients is based on a scientifically well-supported hypothesis-driven approach that alternative mechanisms such as neuroinflammatory and neurotrophic processes are crucially involved in the pathogenesis of depression, and minocycline addresses precisely this issue. In particular, this applies to patients with refractory depression under the hypothesis that neuroinflammatory processes prevent a response to conventional antidepressants. Minocycline, a widely used, generally well-tolerated, and inexpensive drug, may represent an important contribution to the optimized treatment of refractory depression. From a health economic and scientific perspective, but above all from the point of view of the patients affected, this would be a milestone in the treatment of the enormous disease burden of depression.

Translated version of the German trial protocol

2.2 State of knowledge of the investigational drug/therapy under investigation.

The substance under investigation, minocycline, is a widely used representative of the established tetracycline antibiotics. Minocycline is used worldwide as an antibiotic and as an anti-inflammatory substance, both systemically and topically, e.g. in anti-acne therapy. The patent protection for minocycline has expired and numerous generic preparations already exist. It is approved both nationally and internationally.

The investigational product used, Udima 50mg, contains 57.92 mg minocycline hydrochloride dihydrate per capsule, corresponding to 50 mg minocycline. The manufacturer Dermapharm AG, Lil-Dagover-Ring 7, 82031 Grunwald has held the marketing authorization since September 17, 1995, which was extended on January 29, 2007. However, the approval in Germany is expected to expire in July 2018. The manufacturer retains the approval for this medication for Austria. For this reason, we refer to the SMPC of the company at the current time and as a reference document as well as the expert information on the analog preparation Minocycline 100 mg of the company Ratiopharm (see appendix) Information on other ingredients, pharmacokinetics, chemical and physical properties as well as toxicological testing and the mechanism of action can be taken from the manufacturer's expert information.

No increased risks are expected in the context of the indication depression. The combination of minocycline with antidepressants is permissible; there are no indications of addition and potentiation effects or a change in pharmacokinetics. The study's planned dosage of 200mg daily is consistent with an approved dosage. The duration of 6 weeks is a relatively long period for therapy with minocycline at 200mg per day. The administration of 100mg per day is quite common in acne therapy for a period of 6 weeks, for example. In case of an application longer than 21 days, initial and therapy-accompanying controls of differential blood count, renal retention values and liver transaminases are recommended, which is done weekly in the study. Much higher doses of 400mg over a 9-month period have been used in clinically controlled trials [41]. The study drug should be taken, if possible, in the morning and evening at the same time as a meal with plenty of liquid (no milk) usually over a period of 4 - 6 weeks. Taking the drug during a meal may reduce the frequency of gastrointestinal disturbances.

2.3 Question and justification of the project (rationale)

With a prevalence of 8.1% in Germany, unipolar depression is one of the most common mental disorders. Standard therapy with conventional antidepressants does not lead to a sufficient response in a high number of patients, accompanied by a satisfactory reduction of depressive symptoms. In order to achieve an improvement here, new substances in the pharmacotherapy of depression are urgently needed. Minocycline is a CNS-permeable tetracycline with pleiotropic activity. Minocycline is approved for many indications, such as upper respiratory tract infections, due to its antibiotic effects and is characterized by good tolerability. In the context of its pleiotropic efficacy, neuroprotective as well as anti-inflammatory effects not due to the antibiotic action of minocycline have been demonstrated. Depression has a complex pathogenesis that is influenced by neuroinflammatory and neurotrophic mechnanisms in addition to components of neurotransmitter homeostasis. Furthermore, studies show a detrimental influence of neuroinflammatory processes on neurotransmitter homeostasis, which are targets for antidepressants. This could explain the nonresponse to conventional antidepressants. An increase in inflammatory markers such as CRP, TNF-α, and IL-6 is known in depressed patients. In this light, giving minocycline as an adjunctive therapy to antidepressants is a very promising approach. In the treatment of schizophrenic patients, a reduction of depressive symptomatology could be

Translated version of the German trial protocol

achieved. In depressed patients, a treatment trial with minocycline has so far only been conducted in the context of an open trial, in which a significant symptom improvement could be achieved. For an evidence-based answer to the question of whether minocycline is effective as an adjunctive therapy in treatment-resistant major depression, the conduct of a randomized controlled trial is urgently needed.

3 Goals of the clinical trial

The aim of the project is the systematic evaluation of an augmentative antidepressant efficacy of the antibiotic minocycline in patients with unipolar depression who do not show improvement under guideline-based conventional antidepressant pharmacotherapy. This is done by estimating the treatment effect on the Montgomery-Åsberg Depression Rating Scale (MADRS), which measures the severity of depressive symptomatology.

The use of the pleiotropic tetracycline minocycline is blinded over 6 weeks and as an augmentation of conventional antidepressant therapy. Parallel to the blinded treatment, an exact characterization of all patients is performed. In addition to the systematic recording of clinical parameters, the study aims to investigate relevant biomarkers during the course of treatment that are associated with the pathogenesis of depressive disorders and/or a response to antidepressant intervention. The aim would be to identify potential mechanisms of action of the neuroprotective, anti-inflammatory minocycline in treatment-resistant depression and to identify early, and possibly even predict, a response specifically to intervention with minocycline. Among other things, we will investigate whether concomitant therapy with minocycline is more effective in patients with elevation of peripheral inflammatory markers. This is of particular interest in male patients who are known to have a marked inflammatory response associated with depression. An exploratory goal is to analyze effects of minocycline treatment on the expression of inflammatory and neuroplasticity markers and factors involved in cell metabolism in peripheral blood mononuclear cells ("PBMC") and microglial cells. In addition, a determination of the serum concentration of these values will be performed.

Confirmation of the hypothesis of antidepressant efficacy of minocycline would be an important milestone on the way to clinical approval of minocycline in the indication as an augmentation strategy of antidepressant treatment and a breakthrough in the pharmacological treatment of treatment-resistant depression. The knowledge gained from secondary endpoints on possible underlying mechanisms of minocycline will benefit the preclinical further development of antidepressants, e.g. in the optimization of chemically modified, non-antibiotic, but still anti-neuroinflammatory tetracyclines.

Primary outcome

The primary outcome parameter is a change in the Montgomery-Åsberg Depression Rating Scale MADRS) between the beginning and end of the study. The MADRS is a depression questionnaire specifically designed to investigate treatment outcomes in depression. It consists of 10 questions, each of which is scored 0-6. The maximum score is 60, the minimum 0 points. The higher the score obtained, the higher the depressive symptomatology [42]. The MADRS has a high sensitivity to changes in symptom severity and is therefore suitable for monitoring the longitudinal course of depression. The interrater reliability ranges from 0.89 to 0.97. The internal consistency (Cronbach's alpha value) is 0.86. The homogeneity (Loevinger's homogeneity coefficient) is 0.31 [43]. Due to the excellent internal consistency as well as good validity and it finds use in both research and clinical settings [44]. The time points for determining the primary outcome parameter are: Week 1-7, 6 weeks, and 6 months

Translated version of the German trial protocol

after the end of the study. The survey is conducted as a questionnaire during the appointments at the study center. The change in the MADRS value between the intervention and control groups is compared (see statistical analysis). According to the study hypothesis, this change is greater in the intervention group than in the control group.

Primary endpoint

Primary endpoint is change in MADRS score from baseline (week 1) to week 7.

Secondary endpoints

- Response to treatment. This is defined as a reduction in MADRS of more than 50% compared to week 1.
- Remission of depressive symptoms during the study. Remission is defined as reduction of the MADRS score to less than 9.
- Elicitation of the Clinical Global Impressions Scale (CGI-S), global assessment of disease severity.
- Survey of the Beck Depression Inventory (BDI), one of the most widely used depression measurement tools in the world during self-report.
- Survey of the Hamilton Depression Scale (HAMD-17), a third-party assessment scale to assess the severity of depressive symptoms.
- Survey of the symptom checklist SCL-90R, which records the subjectively perceived impairment due to physical and psychological symptoms.
- Survey of Trail Making Tests A and B (TMT A and B) to measure executive function.
- Time points CGI-S, BDI, HAMD-17, MADRS: at the time of screening (not MADRS), during the study in weeks 1-6 and at the end of the study (week 7) as well as 6 weeks and 6 months after the end of the study (not BDI); TMT A and B, SCL 90R: at the time of screening, during the study in week 4 (only SCL-90R) and at the end of the study (week 7) as well as in the 1st follow-up (6 weeks after the end of the study, only SCL-90R). Follow-up (6 weeks after end of study, Scl-90R only) measurement of expression levels in peripheral blood mononuclear cells (PBMCs) and serum concentrations of various cytokines, cell type-specific markers, neurotrophic and anti-inflammatory processes and factors involved in cell metabolism. Time of determination at week 1, 4 and week 7 (after end of study).

3.1 Study design

This is a prospective double-blind, placebo-controlled, two-arm, randomized, multicenter, national proof-of-concept study (phase II according to AMG). A total of 160 patients with a diagnosis of moderate or major depression who have not responded to pharmacotherapy with a standard antidepressant medication at a sufficient dosage for at least 6 weeks during the current episode of illness and who have been receiving stable dosing for 2 weeks will be enrolled in the study. In addition to the Department of Psychiatry and Psychotherapy - Benjamin Franklin Campus, the following investigational sites are planned to participate in the study (documents from the investigational sites listed below will be submitted to the relevant ethics committees at the appropriate time):

- University Hospital and Polyclinic for Psychiatry and Psychotherapy at the Regensburg District Hospital, Universitätsstraße 84, 93053 Regensburg, Germany
- Department of Psychiatry and Psychotherapy, University Medical Center Göttingen, Von-Siebold-Str. 5, 37075 Göttingen, Germany
- Psychiatric Clinic of the LMU Munich, Nussbaumstrasse 7, 80336 Munich, Germany
- Max Planck Institute of Psychiatry, Kraepelinstr. 2-10, 80804 Munich, Germany
- Psychiatric and Psychotherapeutic Clinic, University Hospital Erlangen, Schwabachanlage 6, 91054 Erlangen

Translated version of the German trial protocol

- Heidelberg University Hospital, Department of General Psychiatry, Voßstr. 2, 69115 Heidelberg, Germany
- Department of Psychiatry, Psychotherapy and Psychosomatics, RWTH Aachen University Hospital, Pauwelsstraße 30, 52074 Aachen, Germany
- Department of Psychiatry, Psychosomatics and Psychotherapy, Frankfurt University Hospital, Heinrich-Hoffmann-Strasse 10, 60528 Frankfurt am Main, Germany

In a preliminary examination, the patients are screened with regard to their health and whether they meet the inclusion criteria. Subsequently, patients are randomly assigned to the intervention and control groups. In the intervention group, patients receive the investigational drug minocycyline, and in the control group, they receive the placebo drug. Both groups receive standard antidepressant therapy. Eighty patients will be enrolled in each study arm. The study will last 6 weeks. Pre-study (week 1), as well as weekly during the study (weeks 2, 3, 4, 5, 6), and at the end of the study (week 7), contact occurs at the study center for physical examination, laboratory monitoring, collection of blood parameters, and symptom severity assessment (see Chapter 6). A follow-up visit will take place 6 weeks after the end of the study. In addition, a telephone follow-up appointment will take place 6 months after the end of the study.

Translated version of the German trial protocol

3.2 Schedule

Start of recruitment (first patient first visit, FPFV) >12/2015<

End of recruitment >06/2020<

End of clinical trial (last patient last visit, LPLV) >06/2020<

Duration of individual study participation: 6 weeks

follow up: >02/2016-12/2020<

4 Patient selection

160 patients (see case number estimate in section 14.1) are included. No special gender distribution is taken into account, since no gender-specific differences in the efficacy and safety of the drug to be tested are to be expected (Cf. GCP-V
§ 7 (2) No. 12).

4.1 Inclusion criteria

- performed patient education and written consent (according to AMG §40 (1) 3b)
- Age 18 - 75
- BMI: 18- 40 inclusive
- negative pregnancy test
- adequate contraception (pearl index < 1)
- Meeting the criteria for major depression according to DSM 5
- Hamilton Depression Rating Scale (HAMD-17) score of at least 16
- Clinical Global Impression Scale (CGI-S) score of at least 4
- Pharmacotherapy with an antidepressant approved for the treatment of depression in a sufficient dosage according to the professional information for at least 6 weeks at the time of screening and for at least 2 weeks in a constant dose without a sufficient response according to the criteria of the MGH ATRQ. Simultaneous treatment with other antidepressants or with quetiapine or aripiprazole in any dosage (e.g. for sleep induction) is possible. All antidepressants must have been used for at least 6 weeks prior to screening, including at least 2 weeks in a constant dose.

4.2 Exclusion criteria

- Presence of a neurodegenerative disease
- Presence of a neurological disease in the context of which the depressive symptomatology occurred
- Presence of any severe, unstable somatic disease, including chronic inflammatory diseases such as rheumatoid diseases or inflammatory bowel disease.
- Depressive symptomatology is better explained by the presence of a possibly comorbid other psychiatric disorder, especially a personality disorder. Improvement of more than 50% in HAMD-17 score during the last 14 days.
- Pregnancy and breastfeeding women

Translated version of the German trial protocol

- Substance or alcohol abuse within the past 6 months or a positive urine drug screen (medication with benzodiazepines allowed, as well as nicotine use).
- Thyroid dysfunction (if not euthyroid substituted), severe liver or kidney dysfunction.
- Known autoimmune disease (lifetime prevalence) other than euthyroid Hashimoto's thyroiditis.

- Clinically relevant laboratory abnormalities
- Current medication with anti-inflammatory substances (NSAIDs, corticosteroids)
- Current medication with retinoids (retinol, retinoic acid, or rentionide receptor agonists).
- Any past adverse effect associated with previous tetracycline use, including any hypersensitivity reaction to tetracyclines and tetracycline intolerance
- Any medication that leads to known interactions with minocycline. Should such medication become necessary during the study period, this will result in discontinuation of minocycline.
- Current medication with barbiturates and other anticonvulsant drugs (e.g., carbamazepine, diphenylhydantoin, and primidone).
- Current medication with beta-lactam antibiotics, such as penicillins or cefalosporins.
- Current medication with isotretinoin or medication with isoretionin in the last 4 weeks.
- Current medication with theophylline
- Current medication with sulfonylurea antidiabetics and anticoagulants of the coumarin type
- Current medication with ciclosporin A
- Current medication with methotrexate
- Implementation of methoxyflurane anesthesia or medication with other substances that can damage the kidney
- Lack of willingness to store and share pseudonymized disease data in the context of clinical trials
- Concurrent use of other psychotropic substances in addition to standard antidepressant medication. Exclusively for the therapy of acute agitation/anxiety or severe sleep disorders, lorazepam (max. 4 mg/day) or Z-hypnotics (zolpidem (max. 10 mg/day), zopiclone (max. 7.5 mg/day)) are allowed as comedication.
- Placement in an institution by court or official order
- Participation in a clinical trial according to the AMG within the last 8 weeks
- Patients who are dependent on the investigator, sponsor, or trial site
- Acute suicidality, suicide plans or attempts in the current episode of illness, or suicide attempt within the past 5 years.

4.3 Note on contraception

Use of minocycline during pregnancy and lactation may cause tooth discoloration, enamel defects, and bone growth retardation in fetuses from 4 months of age due to tetracycline incorporation; therefore, participating women must not be pregnant during this clinical trial or become pregnant during the course of the trial. Women of childbearing potential can only be enrolled in the clinical trial if the pregnancy test is negative at the screening visit. Non-breastfeeding, non-pregnant childbearing women may participate in this clinical trial only if you agree to either be heterosexually abstinent for the entire duration of taking the study drug or if you agree to use highly effective methods of contraception (contraception with a

Translated version of the German trial protocol

Failure rate <1%, correct and reliable use during the entire period of taking the study drug The following estrogen-progestin preparations are considered highly effective hormonal contraceptive methods with a Pearl index <1 (contraception with a failure rate <1%): pill, hormone patch and vaginal ring, as well as the following progestin mono-preparations: estrogen-free ovulation inhibitors, injections (three-month injection), hormone implant and hormone coil. Highly effective barrier methods with a Pearl Index <1 include sterilization, vasectomy (sterilized partner), intrauterine devices (IUD): copper coil or copper chain. Should study participants nevertheless become pregnant during the clinical trial, study participation will be terminated immediately.

5 Treatment plan

5.1 Description of the test medication

The study medication is the tetracycline minocycline. The preparation used is Udima® from the company Dermapharm. The dosage form is capsules for oral use containing 50 mg minocycline (57.92 mg minocycline hydrochloride dihydrate). Other ingredients are corn starch, magnesium stearate, gelatin, titanium dioxide (E171), quinoline yellow (E104) and iron oxide red (E172).

The manufacturer mibe GmbH Arzneimittel (a company of the Dermapharm Group) provides verum capsules and external identical-looking placebo capsules free of active ingredients. Both products are packed in blister strips of 10 capsules each. The bulk products Verum and Placebo are labeled blinded in the pharmacy of the Charité according to GCP-V § 5, packed in folding boxes and released by the Qualified Person as Clinical Investigational Product.

The bulk products and investigational medicinal products are stored at 15-30°C. The shelf life results from the declared expiry dates of the bulk batches.

The packing unit consists of 10 blisters per folding box. For each random no. 2 packs are made.

The study dosage is 2 times 2 capsules of 50 mg each, i.e., the daily dose is 200 mg minocycline per day.

5.1.1 List of side effects and interactions

Possible side effects of minocycline include (according to the SmPC):

Gastrointestinal tract: During treatment, gastrointestinal disturbances may occur in the form of heartburn, gastric pressure, vomiting, meteorism, fatty stools and mild diarrhea. Taking after or with meals may reduce these adverse effects to some degree; absorption rate is only marginally affected. Very rarely, inflammation of the mouth and pharyngeal mucosa, hoarseness, and difficulty swallowing have been observed. In the event of severe and persistent diarrhea during or after therapy, the physician should be notified.

Translated version of the German trial protocol

because this can conceal a serious intestinal disease (pseudomembranous enterocolitis) that must be treated immediately.

Nervous System: Central nervous side effects occur significantly more frequently during therapy with minocycline than with other tetracyclines. Women are more frequently affected by these side effects than men. Symptoms include dizziness, nausea, ataxia, and fatigue. Rarely, an increase in intracranial pressure (pseudotumor cerebri) is observed, which is reversible after cessation of therapy. It is manifested by headache, nausea, vomiting, and possibly papilledema. In addition, paresthesias may occur.

Sensory Organs: Very rarely, temporary myopia (nearsightedness) has been observed while taking tetracyclines. Pigment deposits in the eye (cornea, sclera and retina) may occur.

Rarely, tinnitus and hearing deterioration may occur.

Skin and Skin Appendages: Allergic skin reactions to minocycline occur occasionally (see the Hypersensitivity Symptoms section). Exposure to sunlight may cause phototoxic reactions of the exposed skin areas with erythema, skin edema, blistering, and occasionally nail detachment and discoloration. Sunbathing outdoors or in solariums should therefore be avoided during therapy with minocycline.

Patients are additionally advised and instructed by the investigator to limit their exposure to sunlight and UV light, to always wear sunglasses and protective clothing, and to use high SPF sunscreens (including UV-A filters) for uncovered areas of skin. In the area of previous inflammatory skin lesions, blue-gray hyperpigmentation may occur during long-term therapy with minocycline. After prolonged high-dose therapy with minocycline, blackish discoloration of the nails has very rarely been described. In addition, a so-called "black hair tongue" may occur.

Blood and blood cells: In rare cases, as with all tetracycline therapy, the following changes in the blood may be induced and are reversible: Leukopenias, leukocytoses, agranulocytoses, thrombopenias, anemias, eosinophilia, atypical lymphocytes, and toxic granulations of granulocytes.

Liver and bile ducts, pancreas, kidneys: In overdoses, there is a risk of liver and kidney damage and pancreatitis. Immunologically induced hepatitis may occur during therapy with minocycline. In this case, discontinue the drug immediately. Non-immunologic hepatocellular damage and renal damage may also occur, especially with long-term therapy. In the case of long-term use (i.e., more than 21 days), the differential blood count, renal retention values, and liver transaminases should be monitored initially and regularly thereafter.

Hypersensitivity symptoms: Allergic reactions such asgeneralized exanthema, urticaria and erythema, skin itching are common. With unknown frequency, there may also be Quincke's symptomatology, erythema exsudativum multiforme, toxic epidermal necrolysis (Lyell's syndrome (or in milder form a Stevens-Johnson syndrome)), fixed drug exanthema on the genitals and other body regions and serum sickness-like reaction with fever, headache and joint pain. In these cases, immediately discontinue the drug and initiate appropriate countermeasures. Furthermore, pericarditis and exacerbation of systemic lupus erythematosus have been reported.

Drug-induced lupus erythematosus may occur during therapy with minocycline. The risk increases with duration of use.

Translated version of the German trial protocol

Hypersensitivity syndrome (DRESS, fatal in some cases) may occur, resulting in skin reactions (such as rash or exfoliative dermatitis, eosinophilia, and one or more of the following: Liver inflammation, inflammation of the lungs, kidney inflammation, myocarditis or pericarditis, fever, and lymphadenopathy.

Clinically, joint symptoms (polyarthritis and polyarthralgia) with negative rheumatoid factor, fever, fatigue, exanthema, and lymphadenopathy have been described. Eosinophilic pulmonary infiltrates are possible. Laboratory findings may include elevated ESR, eosinophilia, and an elevated ANA titer. In these cases, the drug should be discontinued immediately.

Severe acute hypersensitivity symptoms are possible. They may manifest as: Facial edema, tongue swelling, internal laryngeal swelling with constriction of the airways, heart palpitations, shortness of breath (respiratory distress), drop in blood pressure up to threatening shock. In case of occurrence of these phenomena, immediate medical assistance is required. Within the tetracycline group there is a complete cross-allergy.

Other: During therapy with minocycline, Candida colonization of the skin or mucous membranes may occur with symptoms such as inflammation of the mouth and pharyngeal mucosa (glossitis, stomatitis), acute inflammation of the external genital organs and the vagina in women (vulvovaginitis) and pruritus ani. After prolonged high-dose therapy with minocycline, blackish discoloration of teeth, nails, and bones, as well as of the thyroid gland, has occasionally been described. In the area of preceding inflammatory skin changes, blue-gray hyperpigmentation may occur during long-term therapy with minocycline.

Medication with minocycline may cause an exacerbation of myasthenia gravis.

Contraindications:

Minocycline must not be used in cases of hypersensitivity to minocycline, other tetracyclines or other ingredients, and in cases of severe liver dysfunction.

Even when used as directed, minocycline may alter the ability to react to such an extent that, for example, the ability to actively participate in road traffic or to operate machinery is impaired. This applies to a greater extent in combination with alcohol.

Pregnant and breastfeeding women should not take minocycline because it may cause discoloration of the teeth and slow bone growth in the fetus and infant.

As with all antibiotics, bacteria can develop resistance during treatment with minocycline. There is extensive cross-resistance of minocycline with other tetracyclines. A change in the body's physiological bacterial colonization may occur. In this context, a selection of bacteria resistant to minocycline may occur.

It cannot be excluded that in rare cases the safety of the contraceptive effect of hormonal contraceptives may be questioned during therapy with minocycline. Therefore, additional non-hormonal contraceptive measures (see also chapter 4.3) should be used.

Translated version of the German trial protocol

5.1.2 Treatment scheme

instructions for application:

Of the medication, 2 capsules á 50mg each are taken in the morning and evening with plenty of liquid (no milk). The simultaneous intake of milk or dairy products leads to a reduction of the effect. Taking during a meal may reduce the frequency of gastrointestinal disorders. Aluminum, calcium, and magnesium salts in antacids as well as iron supplements, medicinal charcoal, and colestyramine decrease the absorption of minocycline into the body. These drugs should therefore always be taken 2 to 3 hours before or after the investigational drug. The total dose is 200mg/day.

<u>Treatment duration: week 1-6</u>

		**morning**	**evening**
**Dosage**	50mg/placebo	2	2
**Dosage form**	Capsules		
**Application**	oral		

5.1.3 Storage, issue and return

The investigational medicinal products are stored in the Charité pharmacy or by the Charité tracking center or, after shipment, in the respective trial centers. Access by unauthorized persons can be excluded, as the investigational product is stored in appropriately secured premises.

Dispatch to the test sites by courier service is carried out by the pharmacy or by the site at Charité to the participating test sites. The drug must not be stored above 30 °C.

The study participants receive a package (á 100 capsules) at the beginning of the study (week 1) and a package after 3 weeks of study duration. In week 4 and week 7, the unused capsules are returned to the study centers where they are counted and documented. During monitoring, drug accountability, i.e. the traceability of the consumption of the study medication, is controlled. Previously dispensed packaging and blisters should not be used beyond a subsequent new visit. Unused study medication can be disposed of locally after drug accountability at the center in compliance with applicable guidelines (e.g., via the center's pharmacy) or returned to the sponsor. In either case, disposal must be documented in the investigator's folder. For further information on the investigational medication, please refer to the SmPC.

5.1.4 Compliance

The issued study medication per patient is documented in the Case Report Form. Outpatients will receive one pack of 100 capsules of minocycline at each of two study time points (Week 1 and Week 4). The empty packs or unused units will be returned to the study center at Weeks 4 and 7. The patients record each intake of the drug with date and quantity in the patient diary. The investigator will check the entries at each visit. For inpatients, the above measures are not necessary. They receive their medication daily in the frame by the nursing staff. Compliance will also be checked via therapeutic drug monitoring (serum level measurements of the study medication) at the time points week 1, 3, 5 and 7.

Translated version of the German trial protocol

5.2 Placebo/ comparator medication

The placebo preparation is manufactured analogous to the investigational product by the company mibe GmbH Arzneimittel and provided to the Charité pharmacy in bulk in blister packs. Further packaging and labeling as well as the final release are carried out by the Charité pharmacy. Packaging is analogous to the investigational product in blisters, 10 capsules per blister, 10 blisters per carton. Storage, distribution to the trial sites as well as to the patients is analogous to the investigational product.

The placebo preparation contains the same ingredients, but without the active ingredient. These are corn starch, magnesium stearate, gelatin, titanium dioxide (E171), quinoline yellow (E104), and iron oxide red (E172).

5.3 Concomitant medication

The study medication is given concomitantly with a standard antidepressant therapy. This will be continued according to the dosage before the start of the study. Medications that are not permitted are listed in the exclusion criteria. Any medication must be reported to the investigator prior to the start of the study. If a new medication becomes necessary during the course of the study, the investigator must be notified immediately. Any medication will be precisely documented.

5.4 Emergency measures

In the event of hypersensitivity reactions, immediate medical attention is required. In the event of severe, acute hypersensitivity symptoms with constriction of the airways, heart palpitations, shortness of breath and drop in blood pressure, immediate emergency medical assistance is required. The local emergency control center should be contacted immediately. In both cases, the test center should be informed as soon as possible.

In case of drug intoxication, seek medical attention immediately.

5.5 Blinding and emergency envelopes

<u>Blinding</u>

The production of the verum and placebo capsules is carried out by the company mibe GmbH Arzneimittel, the blinding and completion of the clinical trial preparations by the pharmacy of the Charité. Distribution to the study arms is done by the randomization procedure (see section 6.4). The investigational medicinal products (verum and placebo) are blinded (double-blinded) for both the study physicians and the patients.

<u>Emergency envelopes</u>

The emergency envelopes are prepared by the Charité pharmacy and sent to the study sites together with the study medication. For each study medication, the study sites receive a sealed envelope containing the unblinding (breaking of the code) for the respective drug number. An identical set of sealed envelopes containing the unblinding is with the sponsor. These envelopes contain information about which medication the patient is receiving with the corresponding medication number. The envelopes are to be kept in a safe place.

5.6 Unblinding (breaking the code)

<u>Premature unblinding</u>

Premature unblinding is not intended. In exceptional cases, e.g., in the presence of a life-threatening allergic reaction or the absolute necessity of administering a drug that interacts with minocycline, premature unblinding is possible. The exact circumstances that led to the premature unblinding must be noted on the unblinding envelope.

Translated version of the German trial protocol

Regular unblinding

Regular unblinding will take place after completion of the study. All envelopes are returned to the sponsor and examined for integrity before being opened and analyzed.

6 Course of studies

6.1 Procedure of recruitment / screening

Per center, with the exception of the two study centers in Munich, approximately 32 patients will be recruited during a recruitment period of 30 months. The study centers Psychiatric Clinic of the LMU Munich and Max Planck Institute of Psychiatry, Munich as well as the Psychiatric Clinics of the University Hospital Göttingen and the University Hospital Heidelberg will each recruit approximately 16 patients. This corresponds to approximately 1 patient per month per center, and approximately 1 patient every 2 months for the trial centers in Munich, Heidelberg, and Göttingen. Through their activities in previous and ongoing research on depression, all participating centers have regional psychiatric networks associated with high monthly treatment rates. Based on experience with previous studies and the experience of all participants at the recruitment centers, this recruitment rate is considered realistic. In addition, the study drug used is already approved and recognized in Germany for other indications, which should further facilitate patient recruitment. The inclusion and exclusion criteria are chosen very broadly and allow a higher degree of generalizability of the study results and at the same time allow easier recruitment of study patients.

Recruitment will occur through print and online media and during regular patient contacts with outpatients and inpatients.

6.2 Informed consent procedure

Participation in the study is preceded by a detailed medical consultation at pre-screening or screening in accordance with the requirements of the Declaration of Helsinki (1996) [45] and the ICH-GCP guideline, during which patients are informed about every aspect of the study procedure, the potential individual benefit and the personal risk. Among other things, it is reiterated that participation in the study should be completely voluntary, and the possible alternative treatment options outside the current study are again explained to the patient. Patients are given sufficient time to read the information and to ask any questions that may arise to the investigator providing the information. Participation in the study is only possible after the patient has provided written informed consent. The declaration of consent can be revoked at any time without giving reasons. No examinations or other activities will take place within the scope of the study until the written consent of the participant has been obtained.

A copy of the informed consent and patient education will be given to the patient, as well as a copy of the insurance terms and conditions for participation in the study.

If changes are made to the patient information or the consent form, the participants in the study will be informed of these changes. The new patient information and the consent form are again discussed in detail, the participant is again asked for written consent and the documents are handed over to the patient.

6.3 Procedures to avoid simultaneous inclusion in multiple studies.

Parallel participation in other clinical trials according to the AMG is not possible during participation in the Minocycline Therapy Study. In addition, only subjects who have not participated in any other clinical trial subject to the AMG in the last 8 weeks will be included. This is

Translated version of the German trial protocol

an exclusion criterion and is specifically requested and recorded when the consent form is signed.

6.4 Enrollment, registration, and randomization (allocation of study medication)

Randomized allocation to the respective treatment arms is performed to minimize the influence of random assignment of patients to a treatment arm on the study results.
and to guarantee a purely random distribution. In addition, this is intended to
known and previously unknown variables influencing the patients, so-called confounders, (e.g. demographic factors) on the course of the study and the study outcome should be distributed as evenly as possible between the treatment groups in order to increase the validity of the statistical analyses performed. The double-blind drug treatment conditions should ensure the measurement of clinical parameters and data collection as uninfluenced as possible by expectations towards the study substance.

Patients are randomly assigned at baseline to treatment with either minocycline or placebo. In order to weight the allocation to treatment groups as equally as possible at each of the centers, randomization lists are generated by block randomization, with the pre-determined block length unknown to the centers. The group assignment (or study drug assignment) of an enrolled patient based on the randomization list results from the order of the random numbers of the investigational drugs provided. The randomization list is sent directly to the pharmacy by the study statistician.

Investigators will receive randomization codes for patients at their center in opaque envelopes (emergency letters). This is to allow for the possibility of unblinding the treatment of a study patient in the event of medical necessity. With the exception of this circumstance, blinding of patient treatment should be maintained until the database is closed at the end of data collection and revision or amendment. Envelopes containing randomization codes will be collected at the time of center closure, and the center will be informed of the assignment of each study patient to the appropriate treatment arm after database closure.

Patient allocation to the treatment arms is based on a central randomization code generated by the study statistician Professor Dr. Tim Friede (University Medical Center Göttingen). Randomization will be blockwise, stratified by center, to achieve balanced allocation. The randomization list will be sent directly to the pharmacy by the study statistician. Blinding of evaluators and patients to treatment is possible and will be performed. No unblinding of evaluators regarding specific side effects from minocycline treatment is expected. The pharmacy (Clinical Pharmacist Cornelia Eberhardt, Charité-Universitätsmedizin Berlin, Campus-Virchow- Klinikum, Apotheke, Arbeitsbereich Klinische Prüfpräparate) will be instructed on how to perform proper allocation concealment. Based on the randomization code, the pharmacy will centrally provide each center in the series with the sequentially numbered, tamper evident containers that do not differ in weight or appearance. In addition, an audit trail will be established by writing the subjects' pseudonyms on the empty containers.

To ensure that the study is conducted in accordance with the study protocol, current legislation, and guidelines, training of all coordinators will occur prior to the start of the study.

Translated version of the German trial protocol

6.5 Patient inclusion (enrollment)

Patients who meet all inclusion criteria and have given their consent to participate in the study will be reported to the study center Clinic for Psychiatry and Psychotherapy, Charité - Campus Benjamin Franklin.

To be indicated here are:

- Name and address of the responsible central office/institution
- Name of the responsible employee or contact person
- Telephone and fax connection
- Times of accessibility of the study center
- If the patients can be reported by telephone, the data to be communicated must be listed in the protocol
- Investigation center, investigator
- Pseudonym of the patient to be randomized
- Gender
- Diagnosis/ Drug therapy

It is preferable to register by fax with the registration form on which the necessary information has been entered.

6.6 Clinical examinations and deviations from standard clinical practice, visit schedule.

Translated version of the German trial protocol

**6.6.1.1 Study plan**	Visit -1	Roun ds 1	Roun ds 2	Visit 3 to 7	Roun ds 8	Visit 9 Or earlier in the case of premature termination of studies	Visit 10 Telephone follow-up
Investigation	Prescreening	Screening (up to 1 weeks before)	Start of study Day 0, Week 1	Weeks 2,3,4,5,6	End of the study Week 7	Follow-up Week 13	follow-up 6 months
Informed consent	**X**	**X**					
Selection of suitable study participants	**X**	**X**					
Optimization of therapy if necessary	**X**						
Medical history		**X**					
MGH-ATRQ		**X**					
Childhood Trauma Questionnaire			**X**				
Barratt Impulsivity Scale			**X**				
TMT A, B			**X**		**X**		
HAMD-17		**X**	**X**	**X**	**X**	**X**	**X**
MADRS			**X**	**X**	**X**	**X**	**X**
CGI-S		**X**	**X**	**X**	**X**	**X**	**X**
BDI		**X**	**X**	**X**	**X**	**X**	
SCL-90R			**X**	**Wk. 4**	**X**	**X**	
Clinical interview			**X**	**X**	**X**	**X**	**X**
Whole blood sample (PBMC; transcriptome)			**X**		**X**		
Serum analysis (inflammatory parameters, neurotrophic parameters, etc.)			**X**	**Wk. 4**	**X**		
Genotype determination			**X**				
Physical examination		**X**	**X**	**X**	**X**	**X**	
Vital signs, weight		**X**	**X**	**X**	**X**	**X**	
Size		**X**					
Pregnancy test		**X**		**Wk. 4**	**X**		
Urine drug screening		**X**					
Safety laboratory		**X**	**X**	**X**	**X**		
Drug level			**X**	**Wk. 3 + 5**	**X**		
Adjunctive therapy		**X**	**X**	**X**	**X**	**X**	
Adverse events			**X**	**X**	**X**	**X**	**X**
Issue of the study medication (outpatients only)			**X**	**Wk 4**			

Translated version of the German trial protocol

Return of the study medication (outpatients only)				**Wk. 4**	**X**		

Translated version of the German trial protocol

6.6.1.1 **Study plan**	Visit -1	Roun ds 1	Roun ds 2	Visit 3 to 7	Roun ds 8	Visit 9 Or earlier in the case of premature termination of studies	Visit 10 Telephone follow-up
Patient diary (outpatients only)			**X**	**X**	**X**		
Monitoring of suicidality		**X**	**X**	**X**	**X**	**X**	**X**

Translated version of the German trial protocol

6.6.2 Pre-screening and screening examination (visit 1)

During pre-screening or screening, the patient is informed. If the patient consents to the study after sufficient time to think about it, the screening examination follows. In addition, the patient's current medication is evaluated. With the patient's consent, therapy may be optimized or switched to conventional antidepressant medication.

During the screening examination, the fulfillment of the selection criteria is checked. In addition, the baseline data relevant for efficacy and tolerability assessment are collected.

The maximum time interval between screening examination and study initiation is 1 week.

The screening examination program includes:

- Medical history and physical examination
- Confirmation of diagnosis by means of a clinical interview (e.g., Composite International Diagnostic Interview (CIDI)).
- Body height, body weight
- Blood pressure, pulse
- MGH-ATRQ
- HAMD-17, CGI-S, BDI
- Laboratory testing incl. safety laboratory (cf. section 6.6.9)
- Urine drug screening
- Pregnancy test
- Assessment of suicidality (by means of item 10 "Suicidal thoughts" in the MADRS score).
- Recording of the concomitant therapy, possibly optimization of the medication

6.6.3 Initial examination (visit 2)

The entry examination will take place at the beginning of the study on day 0 of week 1. The examination program of the initial examination includes:

- Physical examination
- Body weight
- Blood pressure, pulse
- Clinical interview
- HAMD-17, CGI-S, BDI, MADRS, SCL-90R
- Assessment of working memory using the Trail Making Tests A and B (TMT A, B)
- Capture early childhood traumatic events by means of the Childhood Trauma Questionnaire (CTQ)
- Assessment of impulsivity using the Barratt Impulsivity Scale.
- Assessment of suicidality (by means of item 10 "Suicidal thoughts" in the MADRS score).
- Acquisition of concomitant therapy
- Laboratory testing incl. safety laboratory (cf. section 6.6.8)
- Determination of drug levels
- Adverse event survey
- Further clinical diagnostics (see point 6.7)

Outpatients are also given the study medication. Inpatients receive the medication daily on the ward, provided by the nursing staff. Outpatients also receive a patient diary in which they document each intake of medication.

Translated version of the German trial protocol

6.6.4 Follow-up examinations (visits 3-7)

Follow-up visits will take place during the study weeks 2-6. The visits take place every 7 days (±1 day). The examination program of the follow-up examinations includes:

- Physical examination
- Body weight
- Blood pressure, pulse
- Clinical interview
- HAMD-17, CGI-S, BDI, MADRS
- SCL 90R (in week 4)
- Assessment of suicidality (by means of item 10 "Suicidal thoughts" in the MADRS score).
- Acquisition of concomitant therapy
- Control patient diary (only outpatients)
- Laboratory testing incl. safety laboratory (cf. section 6.6.8)
- Pregnancy test (in visit 5, week 4)
- In rounds 4 and 6 (weeks 3 and 5), the determination of drug levels also takes place
- Adverse event survey

In the case of outpatients, the study medication is also dispensed again during visit 5. For outpatients, the remaining medication and the empty medication containers are also returned during visit 5.

6.6.5 Final examination (visit 8)

The final examination takes place at the end of the study participation (week 7). This examination will take place as far as possible in all patients who leave the study prematurely (in the case of patients who leave voluntarily, with their consent).

The examination program includes:

- Physical examination
- Body weight
- Blood pressure, pulse
- Clinical interview
- HAMD-17, CGI-S, BDI, MADRS, SCL-90R
- Assessment of suicidality (by means of item 10 "Suicidal thoughts" in the MADRS score).
- Assessment of working memory using the Trail Making Tests A and B (TMT A, B)
- Acquisition of concomitant therapy
- Return of the patient diary (for outpatients)
- Laboratory testing incl. safety laboratory (cf. section 6.6.8)
- Pregnancy test
- Determination of drug levels
- Adverse event survey

In addition, for outpatients, the return of leftover medication and empty medication containers takes place.

Translated version of the German trial protocol

6.6.6 1st follow-up examination (visit 9)

The first follow-up visit will take place 6 weeks (± 5 days) after the end of the study, i.e. at week 13.

It includes:

- Physical examination
- Body weight
- Blood pressure, pulse
- Clinical interview
- HAMD-17, CGI-S, BDI, MADRS
- SCL 90R
- Assessment of suicidality (by means of item 10 "Suicidal thoughts" in the MADRS score).
- Acquisition of concomitant therapy
- Adverse event survey

6.6.7 2nd follow-up:

The second follow-up visit will take place 6 months (±1 week) after the individual study end date. It is a telephone contact.

This includes:

- Clinical interview
- HAMD-17, CGI-S, MADRS
- Assessment of suicidality (by means of item 10 "Suicidal thoughts" in the MADRS score).
- Adverse event survey

6.6.8 Survey of outcome parameters:

MADRS

The Montgomery-Åsberg Depression Rating Scale (MADRS) is a depression questionnaire specifically designed to examine treatment outcomes in depression by assessing depressive symptomatology. It consists of 10 questions, each of which asks about depressive symptoms, such as suicidal ideation, sleep disturbance, and sadness. If a symptom is answered in the negative, this means 0 points; if it is answered in the affirmative to the highest degree, 6 points. Maximum score to be achieved is therefore 60, and the minimum score is 0. The higher the score achieved, the higher the depressive symptomatology [43]. The time points of determination are: Week 1-6, 7, 6 weeks, and 6 months after the end of the study. The survey is done as a questionnaire during the appointments at the study center. If possible, the examination should always be performed by the same investigator during the course of the study.

HAMD-17

The Hamilton scale (HAMD-17) is used for quantitative assessment in depressed patients. It is a third-party assessment scale for surveying the severity of depressive symptoms. It consists of 17 questions, each of which asks about depressive symptoms, such as suicidal ideation and sadness. The examiner assesses how severe a symptom is, depending on the question on a scale of 0-2 or 0-4 [46]. The time points of determination are: Week 1-6, 7, 6 weeks, and 6 months after the end of the study.

Translated version of the German trial protocol

CGI-S

The Clinical Global Impressions-Severity-Scale (CGI-S) is used for the global assessment of disease severity using a 7-point rater-based assessment. A score of "1 "describes the absence of symptoms, and a score of "7" describes an extremely severe expression of disease [46]. The time points of assessment are: Week 1-6, 7, and 6 weeks after the end of the study.

BDI

The Beck Depression Inventory (BDI) is a 21-question questionnaire that patients complete themselves [47]. It queries depressive symptoms, such as loss of appetite and lack of interest. On a scale of 0-5, 0 means absence of symptomatology and 5 means full expression. The time points of assessment are: Week 1-6, 7, 6 weeks, and 6 months after the end of the study.

SCL-90R

The Symptom Checklist 90R (SCL-90R) is a self-report questionnaire in that patients assess their own psychological distress. It consists of 90 questions from the domains: Aggressiveness/Hostility, Anxiousness, Depressiveness, Paranoid Thinking, Phobic Anxiety, Psychoticism, Somatization, Insecurity in Social Contact, and Obsessiveness [48]. The time points of assessment are: Week 1-6, 7, 6 weeks, and 6 months after the end of the study.

Trail Making Tests A and B

A large number of depressed patients suffer from executive function disorder. Trail testing in the context of measuring the effectiveness of a novel therapy is recognized and can be investigated using the Trail Making Tests (TMT A, B). It gives information about processing speed, mental flexibility and executive functions [49].

The respective visits will last a maximum of 90 minutes. The visit at the beginning of the study (week 1) can be longer with approx. 120 minutes.

6.6.9 Laboratory tests / handling of samples

As part of the clinical trial, a weekly safety laboratory draw and analysis, including a differential blood count (see below), will be performed during minocycline use. The test material for the designated laboratory tests will be obtained by venous blood collection (standard procedure). Hematology and clinical chemistry are performed by the local laboratory of the respective test center.

As an example for the reference values of the undercutting or exceeding of the measured parameters of the laboratory examination of hematology and clinical chemistry, the threshold limit used for the apparative equipment of the central laboratory of the Charité, CVK, Labor Berlin - Charité Vivantes GmbH, Sylter Straße 2, 13353 Berlin are listed below. Depending on the equipment of the local central laboratory of the clinic in the corresponding additional testing site, these may differ (see the corresponding certificates of the laboratories).

Translated version of the German trial protocol

The following laboratory tests are performed at the screening visit and visits 2-8:

<u>Hematology:</u>

Erythrocyte material: blood in EDTA
Method: Impedance measurement Reference values:
m up to 65 years 4.3 to 5.8/pl w up to 65 years 3.9 to 5.2/pl m >65 years 4.0 to 5.6/pl
w >65 years 3.8 to 5.2/pl

Platelet Material:
Blood in EDTA
Method: Impedance measurement Reference values: 150 to 370/nl

Translated version of the German trial protocol

Hemoglobin
Material: Blood in EDTA
Method: photometric determination Reference values:
m to 65 years 13.5 to 17.0 g/dL
w to 65 years 12.0 to 15.6 g/dL
m > 65 years 12.5 to 17.2 g/dL
w > 65 years 11.8 to 15.8 g/dL

Hematocrit
Material: Blood in EDTA Method: Calculation Reference values:
m to 65 years 40 to 51%
w to 65 years 36 to 46%
m > 65 years 37 to 49%
w > 65 years 35 to 46%

Leukocytes
Material: Blood in EDTA
Method: Impedance measurement Reference values: 3.9 to 10.5 /nl

Basophils absolute
Material: Blood in EDTA
Reference values: 0 to 0.2 /nl

Eosinophils absolute
Material: Blood in EDTA
Reference values: 0.02 to 0.5 /nl

Translated version of the German trial protocol

Lymphocytes absolute
Material: Blood in EDTA
Reference values: 1.1 to 4.5
/nl

Monocytes absolute
Material: blood in
EDTA
Reference values: 0.1 to 0.9 /nl

Neutrophils absolute
Material: blood in EDTA
Reference values: 1.5 to 7.7
/nl

<u>Metabolites:</u>

Creatinine
Material: Serum
Method: Color
test Reference
values:
Women 44.0-80.0 µmol/L
Men 62.0-106 µmol/L

TSH (only once for screening test) Material: Serum
Method: Electro chemiluminescence immunoassay
Reference values: 0.27- 4.20 mU/l

<u>Liver enzymes:</u>

ASAT (GOT)
Material: Serum
Method: enzymatic color test Reference
values:
m <50 U/l
w < 35 U/l

ALAT (GPT)
Material: Serum
Method: enzymatic color test Reference
values:
m <41 U/l
w < 31 U/l

γ-GT (gamma-glutamyltransferase)
Material: Heparin blood
Method: photometric determination Reference
values:
m 8-61 U/l
w 5-36 U/l

Translated version of the German trial protocol

Electrolytes:

Sodium
Material: Heparin blood
Method: Ion-selective electrode Reference values: 131 - 146 mmol/l

Potassium
Material: Heparin blood
Method: Ion selective electrode Reference values: 3.4-4.5 mmol/l

Clinical Chemistry:

Lipase
Material: Heparin blood
Method: photometric determination Reference values: 13 to 60 U/l

HbA1c (only once for screening test) Material: blood in EDTA
Method: High performance liquid chromatography
Reference value: <6%.

HDL cholesterol
Material: Serum
Method: immunoturbidimetry
Reference values:
m ≥35 mg/dl
w ≥45 mg/dl

LDL cholesterol
Material: Heparin blood
Method: photometric determination Reference values: below 130 mg/dl

Total cholesterol
Material: Serum
Method: enzymatic color test Reference value: Up to 200 mg/dl

Triglycerides
Material: Heparin blood
Method: photometric determination Reference values: ≤200 mg/dl

CRP highly sensitive
Material: Serum
Reference values not available

Translated version of the German trial protocol

Urinalysis:
Drug screening (one-time screening only).

Therapeutic Drug Monitoring (TDM):

The TDM takes place in the laboratory of the Max Planck Institute of Psychiatry, Kraepelinstr. 2-10, 80804 Munich. Reference values are not available. Determination times are 1, 3, 5 and 7 per week.

Other laboratory tests:

Measurement of expression levels in peripheral blood mononuclear cells (PBMCs, only in patients of the Charité study center) and serum concentrations of various cytokines, cell type-specific markers, neurotrophic factors and factors involved in inflammatory processes and cell metabolism. The determination times are week 1,3 and week 7 (after the end of the study). The examinations take place in the in-house neurobiological laboratory of the Clinic for Psychiatry and Psychotherapy, Charité
- Campus Benjamin Franklin, Hindenburgdamm 30, 12203 Berlin.

Approximately 70 ml (without PBMCs: 30 ml, PBMC analysis only for patients at the testing center as Berlin) of blood is required for these laboratory tests.

Genotype determination of therapy-influencing genetic variants (e.g. rs2032583, rs2235015, rs2235040, rs1045642, rs2032582, rs1128503 in the ABCB1 gene). The determination time point is in week 1.
The examination takes place in the laboratory of the Max Planck Institute of Psychiatry, Kraepelinstr. 2-10, 80804 Munich. Approximately 8 ml of blood is required for these laboratory tests.

In the course of the study, venous blood samples will be taken eight times. The amount of blood corresponds to five times approx. 15 ml, once approx. 35 ml, once approx. 80 ml as well as once approx. 88 ml blood per collection for patients at the study center Berlin, for patients at the other centers: five times approx. 15 ml, twice approx. 45 ml as well as once approx. 53 ml. Of these max. 88 ml blood, approx. 17-70 ml blood will be sent to the Neurobiological Laboratory of the Clinic for Psychiatry and Psychotherapy, Charité - Campus Benjamin Franklin, Berlin for further laboratory tests, depending on the time point and study center, and approx. 8.5 (or week 1: 16.5 ml) at the time points week 1, 3, 5 and 7 to the laboratory of the Max Planck Institute for Psychiatry, Kraepelinstr. 2-10, 80804 Munich for TDM and genotyping. The remaining approx. 10 ml of blood will be analyzed and stored - as for the other blood collections - in the local "Central Laboratory/Laboratory for Clinical Chemistry" of the locally involved testing laboratory, which is contractually connected to this laboratory. This is somewhat more than in the context of routine collections, but a harmless quantity for adults.

Encoding of the samples:

The samples are stored and analyzed in pseudonymous form. No name, date of birth or address of the participant is mentioned. As a pseudonym, each participant receives an abbreviation consisting of the identification letter of the center and a consecutive number (e.g. Mino001). The coded samples are sent to the respective local contract laboratory of the respective local test center (for example, for the test center of the LKP, this is the central laboratory CVK, Laboratory Berlin - Charité Vivantes GmbH, Sylter Straße 2, 13353 Berlin) as the sample recipient, and examined according to the listed laboratory parameters. The analysis of the samples for Therapeutic Drug Monitoring (TDM) are sent across centers, also encrypted, to the laboratory of the Max Planck Institute for Psychiatry, Kraepelinstr. 2-10, 80804 Munich. The shipment of samples for further analyses (serum concentration of various

Translated version of the German trial protocol

cytokines, cell type specific markers, neurotrophic as well as anti-

Translated version of the German trial protocol

inflammatory processes and factors involved in cell metabolism) is sent to the Neurobiological Laboratory, Department of Psychiatry and Psychotherapy, Charité - Campus Benjamin Franklin, Hindenburgdamm 30, 12203 Berlin.

Storage period:

The samples in the Neurobiological Laboratory of the Charité as well as in the MPI Munich will be stored for a maximum period of 5 years after the end of the study for the purpose of post-testing the study results and will be destroyed afterwards. There will be no permanent storage of the samples. Other samples will be stored for a maximum period of 4 weeks. The storage of the samples should be done by the analyzing laboratory, unless the samples were consumed during the analysis.

Discarding of Samples:

Unused sample material will be stored for a maximum period of 5 years after the end of the study and then destroyed.

6.7 Duration of study participation for the individual patient

End of regular treatment / study participation

The regular end of the patient's participation in the study is after 6 weeks of therapy with the study medication. Follow-up continues for 6 months after the end of therapy in the form of two follow-up visits (see Section 6.6.5 and 6.6.6). A change of the treatment arm is not planned.

7 Risk-benefit assessment

7.1 Risks, side effects, burdens, advantages and disadvantages for the participant

In light of the urgent need for new antidepressant medication, a clinical trial to test new compounds is needed.

The study has a relatively short duration of 6 weeks. In addition to a pre-examination, a follow-up examination and a telephone appointment in the follow-up, there are 8 visits during the course of the study and 2 follow-up appointments. It should be noted that these are sick patients who have regular examinations due to their drug therapy even without the study. They are therefore only slightly more burdened than they would be in the course of a routine therapy outside the study.

There will be no invasive procedures except for intravenous blood sampling, the risks of which will be explained to the subjects as part of the study information. The standard therapy will not be withheld from the patients during the study (see 2.1).

According to the study, the main risk for patients is medication with minocycline.

Minocycline is a long-tested and proven drug that is approved under the German Medicines Act for the treatment of infectious diseases and acne. Side effects and contraindications are known, and participants are informed about them in detail. As with any substance, new, previously unknown side effects may occur with the use of minocycline. According to current knowledge, a clustered occurrence of adverse events is not to be expected. Indication-specific risks in the treatment of depressed patients are also not expected. Minocycline is used as standard treatment for infections of the respiratory tract and the ear, nose, and throat, for infections of the genitourinary tract, and for other infections,

Translated version of the German trial protocol

of the gastrointestinal tract as well as in dermatological indications such as acne treatment and is approved for these indications. The standard daily dosage varies in the range of 50 mg to 200 mg. The duration of administration is usually up to 6 weeks.

In this study, minocycline is given orally at the approved dosage of 2x 100 mg minocycline per day. The medication can be decreased or discontinued at any time if needed. No tapering is necessary. Minocycline will be administered for 6 weeks as part of the study. The clinical information (see appendix) recommends initial and concomitant monitoring of differential blood counts, renal retention values, and liver transaminases beyond 21 days of minocycline administration, which will be performed weekly during the study. In other clinical trials in the psychiatric context, minocycline at the dosage of 200mg/day has already been given for periods of 8 weeks without serious adverse events occurring and without any significant change in the side effect profile in the minocycline and placebo groups when given as add-on therapy [33].

The placebo has the same composition as the verum, but without the active ingredient. Pharmacological effects or side effects are not assumed.

If the study hypothesis is confirmed, minocycline is expected to lead to a significant reduction in depressive symptoms up to remission of the disease. Since patients classified as treatment-resistant up to that point are being treated in particular, an individual benefit may also be expected if the participant receives the verum. However, an individual benefit cannot be guaranteed, which is expressed in the patient information.

The results obtained from the study will provide valuable insights into the pathomechanism of depression and in particular a possible involvement of neuroinflammatory processes as a pharmacologically modulable target in treatment-resistant depression. This alone may enable the targeted development of new antidepressant therapeutic options for patients previously classified as treatment-resistant. In the short and medium term, the results may represent an important milestone on the way to a clinical approval of minocycline in the indication of augmentation of antidepressant treatment.

Study participation is associated with at most a minor additional patient burden.

In addition to the considerable general gain in knowledge, there is a possible benefit for the individual patient. Against the background of the generally low risk of the drug minocycline, which is used worldwide in its antibiotic indication and has been established and well tolerated for decades, the offer of participation in the study is well justifiable from a medical point of view.

8 Termination and further treatment

8.1 Premature study termination of a single patient

<u>Premature withdrawal of a patient from the study (discontinuation criteria):</u>

- Occurrence of serious adverse events <u>if they are likely to be related to the study drug</u>
- Paraclinical parameters assessed as clinically relevant by the investigator and collected in the safety laboratory, which may indicate disturbances in liver and kidney and pancreas function, changes in blood count, and electrolyte balance,
- Any evidence of minocycline-related allergic reaction,
- Any evidence of liver or kidney toxicity during regularly scheduled physical examinations,
- Occurrence of clinical or paraclinical signs of other minocycline-associated side effects, such as dental or skin discoloration, severe headache, visual disturbances,

Translated version of the German trial protocol

Pericarditis, nephritis, hepatitis, pancreatitis, Systemic Lupus Erythematosus, severe diarrhea with evidence of pseudomembranous colitis ,

- Withdrawal of consent,
- Occurrence of new concomitant diseases that make further study participation not possible/ not reasonable;
- Violation of inclusion criteria,
- Subsequent occurrence of an exclusion criterion
- Occurrence of acute suicidal tendencies: Assessment by means of item 10 "Suicidal thoughts" in the MADRS score, discontinuation if > 4 points.

8.2 Premature termination of the entire clinical trial

Premature end of study / discontinuation of the entire study

Reasons for premature termination of the entire study may include:

- Decision of the study management in case of unacceptable risks and toxicities under benefit-risk consideration.
- new (scientific) findings during the duration of the clinical trial that may jeopardize the safety of the trial participants (positive benefit-risk assessment no longer given)
- Request from the sponsor (if applicable, the LKP) or the responsible higher federal authority
- safety reasons on the advice of the independent Data and Safety Monitoring Board (DSMB) deemed it necessary to terminate the study;
- Lack of recruitment.

Decision-making body

The decision to discontinue the study may be made by:

- Study management, if necessary in coordination with the sponsor
- The Data and Safety Monitoring Board (DSMB) may also make a recommendation to discontinue the study, on which the sponsor must then take a position.

8.3 Plan for continuing treatment and medical care after training/graduation

Further procedure after retirement

Patients will continue to receive treatment as part of routine care upon discontinuation of therapy, just as they do after the end of the study, and will be examined according to the same schedule as after the regular end of therapy. They will also be examined, as far as possible, at the regular follow-up examinations.

Further procedure after completion

After completion of the study, patients will continue to be treated as part of routine care.

Translated version of the German trial protocol

9 Adverse events

9.1 Definitions

Adverse Event (AE)

"Any adverse event that occurs to a patient or subject who has been administered a drug and that is not necessarily causally related to that treatment"
These may be conditions, signs of illness, or symptoms that occur or worsen after the patient is included in the study.
The expression is evaluated as follows:

- easy
- moderate
- heavy
- Whether the criteria for an SAE are met

A causality assessment must be made for each event:

- no connection
- possible link
- probable link

Serious Adverse Event (SAE)

Serious is an Adverse Event that is.

- fatal or life-threatening,
- requires inpatient treatment or its prolongation,
- results in a permanent or serious disability or incapacity,
- or results in a congenital anomaly or birth defect.

Suspected case of a unexpected serious Adverse Reaction" (Suspected Unexpected Serious Adverse Reaction - SUSAR).

SUSARs are suspected cases of unexpected serious adverse reactions, with the following criteria:

- Type or severity is not consistent with the available information on the investigational product (GCP-V §3 (9)).
- severe:
  - Life-threatening or fatal
  - Inpatient treatment required or prolonged
  - Permanent or serious disability or incapacity
  - Birth defect or congenital anomaly
- Adverse reaction (adverse and unintended response to an investigational drug, regardless of its dosage, GCP-V § 3 (7)).

This refers to side effects that are not described in the SmPC and are classified as both unexpected and severe in their occurrence (e.g., severe intensity, more frequent occurrence).

Translated version of the German trial protocol

9.2 Intensity assessment

Mild: The adverse event is transient and easily tolerated by the patient.

Moderate: The adverse event causes inconvenience to the patient and interferes with the patient's usual activities.

Severe: The adverse event causes significant disruption to the patient's usual activities.

9.3 Assessment of the causal relationship

The following definitions are used to assess the relationship between the use of the test product and an AE:

Safe: A response that follows a traceable time course after use of the test product or in which drug concentration has been measured in body tissue or fluid, follows a known or expected pattern of response to the suspected test product, and disappears after discontinuation or dose reduction and recurs upon re-exposure.

Probable: a response that follows a traceable time course after use of the investigational product, follows a known or expected pattern of response to the suspected investigational product, and disappears after discontinuation or dose reduction and cannot be explained by the known characteristics of the patient's clinical condition.

Possible: A response that follows a traceable time sequence after the test product is used, follows a known or expected pattern of response to the suspected test product, but could easily have been caused by a number of other factors.

Unlikely: A reaction that follows a comprehensible time course after the time of its occurrence makes a connection with the administration of the drug unlikely and that can be explained by other circumstances, such as concomitant diseases or comedication.

Unrelated: A reaction for which there is sufficient information to assume that there is no relationship to the test product.

Not assessable: An assessment of the correlation is not possible.

9.4 Documentation of AEs and SAEs

All serious adverse events (SAEs) as well as all adverse events (AEs) are documented, regardless of whether or not the investigator believes there is a causal relationship with the investigational drug. Documentation includes the type of event, onset, duration, manifestation/severity, and causality.

As far as possible, related signs, symptoms and changes in laboratory values are combined into a single disease. The documentation sheet (CRF) is available for documentation purposes. SAEs are additionally documented on a separate SAE sheet.
Laboratory data that lie outside the normal range are evaluated by the investigator with regard to their clinical significance and - if relevant - are also recorded as an adverse event.

All adverse events are followed until resolved or stabilized.

Translated version of the German trial protocol

9.5 Reporting of SAEs and suspected cases of serious unexpected adverse reactions (SUSARs).

The investigator will notify the sponsor immediately (within 24 hours) after becoming aware of the occurrence of a serious adverse event and subsequently provide the sponsor with a detailed written report.

The report should be sent by fax to:

Charité Universitätsmedizin Berlin
Coordination Center for Clinical Studies KKS
Charité
Augustenburger Platz 1
D-13353 Berlin
Fax number: 030 4507553856

A separate SAE report form will be completed for each of these events and promptly forwarded to the address provided. If the required information is not available at that time, follow-up reports must be completed. For deaths, a copy of the autopsy report should be included if possible.

The sponsor documents in detail all adverse events reported to him by the investigators. Upon request, the sponsor shall forward these records to the competent higher federal authority and to the competent authorities of other EU member states and other states party to the Agreement on the EEA in whose territory the study is conducted.

The sponsor reports every suspected case of an unexpected serious adverse reaction (SUSAR) that comes to his attention without delay, but at the latest within 15 days of becoming aware of it, to the competent ethics committee, the competent higher federal authority and the competent authorities of other EU member states and other signatory states to the Agreement on the *EEA in whose territory the study is being conducted. Furthermore,* he/she shall inform all investigators involved in the study.

In the event of a SUSAR that has led to a death or is life-threatening, the sponsor shall immediately, but no later than 7 days after becoming aware of it, provide the competent ethics committee, the competent higher federal authority and the competent authorities of other EU member states and other states party to the Agreement on the EEA in whose territory the study is being conducted, as well as all investigators involved, with all information important for the evaluation and, within a maximum of 8 further days, with the other relevant information.

The sponsor shall inform the competent ethics committee, the competent higher federal authority and the competent authorities of other Member States of the EU and other states party to the Agreement on the EEA in whose territory the study is being conducted without delay, but at the latest within 15 days of becoming aware of it, of any facts that require a renewed review of the benefit-risk assessment of the investigational medicinal product. This includes in particular:

- Individual case reports of expected serious adverse events with an unexpected outcome
- Increase in the frequency of expected serious adverse events that is judged to be clinically relevant.

Translated version of the German trial protocol

- SUSARs that occurred after the subject had already completed the clinical trial
- Events related to the conduct of the study or the development of the investigational product that could potentially affect the safety of affected subjects

Personal data is always pseudonymized before it is transmitted. Before reporting a SUSAR, the blinding is removed for this patient.

9.6 Pregnancies

The occurrence of pregnancy must be reported to the sponsor within 24 h of becoming aware of it. A pregnant patient will be excluded from the study immediately.

Patients who become pregnant during the study should be observed at least until the outcome of the pregnancy is known.
This includes spontaneous or voluntary termination, details of delivery, the presence or absence of birth defects, malformations, or complications in the newborn and/ or mother.
Documentation will be on the pregnancy report form faxed to the sponsor. Follow-up is done on the same form, within 24h at termination of pregnancy and within 4 weeks after birth.

For the follow-up of the pregnancy, a separate consent form must be obtained from the patient, which allows information about the course and outcome of the pregnancy to be reported. This also applies in the event that the partner of a study participant becomes pregnant.

9.7 Contact person and person responsible for the messages

Charité Universitätsmedizin Berlin
Coordination Center for Clinical Studies KKS
Charité
Augustenburger Platz 1
D-13353 Berlin
Fax number: 030 4507553856

9.8 Data and Safety Monitoring Board

The study is monitored by an independent Data and Safety Monitoring Board (DSMB). To ensure the highest possible safety and vigilance, members of outstanding competence were specially selected: an expert in neuroinflammation (H. Kettenmann, President of the Max Delbrück Center for Molecular Medicine (MDC), Cellular Neurosciences, Berlin) as well as in depression (M. Weber, Managing Senior Physician of the Max Planck Institute of Psychaitry, Munich) and in neuroimmunology (F. Paul, NeuroCure Clinical Research Center, Charité - Universitätsmedizin Berlin). Members of the DSMB are excluded from participation in the review group. The DSMB will coordinate at least once a year prior to study initiation and during the study, the preferred method being by teleconference. The purpose of these meetings is to monitor study success, safety data (SAEs), and protocol compliance.

Translated version of the German trial protocol

Details will be addressed in a DSMB charter to be coordinated with the DSMB. The study will only continue if it is judged safe by the DSMB.

10 Documentation

10.1 Patient documentation forms (CRF)

The data collected will be documented in the eCRF (electronic).

10.2 Principal investigator folder

All essential documents according to ICH GCP Chapter 8 are filed in the investigator's folder on site at the trial center.

10.3 Documentation of the study medication (drug accountability)

The study medication (verum and placebo) will be shipped by the Charité pharmacy as final releasing manufacturer to the study sites according to request. The incoming and outgoing medication will be documented in the Charité pharmacy as well as in the study sites or the involved local pharmacies of the participating study sites. The documentation of the study medication takes place in corresponding logs. In the local trial sites, the investigators have to control and document the incoming and outgoing of the study medication. The dispensing to patients and the taking back of unused study medication is documented by the study physicians at the study sites.

11 Quality management

The planned and systematic quality assurance measures to ensure that the clinical trial is conducted in accordance with GCP and the applicable regulatory requirements must be described. These include procedures for standardization (Standard Operating Procedures) and validation of measurement methods, control of study conduct (monitoring), control of data quality, and measures for independent assessment of critical data (independent data monitoring committee).

11.1 Monitoring of the study process and data quality

The following criteria, for example, can be mentioned as quality indicators for the study process:

- Compliance with the recruitment rate
- Compliance with the selection criteria
- Compliance with the randomization principle / blinding
- Compliance with the treatment according to the protocol
- Compliance with the examination and evaluation deadlines

11.1.1 Monitoring

The monitoring is performed by the KKS of the Charité. The study site is supported by an experienced monitor from the KKS Charité to ensure patient safety and data quality during a clinical study. The monitor checks whether patient safety and the rights of the study participant are respected and adhered to at the study site. It also aims to ensure that valid as well as complete study data are collected and that the study is conducted in accordance with the study protocol, GCP guidelines and current legislation. The type and extent of monitoring will be determined by the study director.

Translated version of the German trial protocol

The investigator agrees that the monitor will visit the study site on a regular basis and that the monitor will receive appropriate support in his/her work at the study site and unrestricted access to study-related documents.
The monitor may match the collected data in the CRF with the source data in the study participant's medical record if the study participant consents, according to the privacy policy in the patient consent form.

The purpose of monitoring is:

- Control of proper study participant education process and signed patient consents,
- Review patient safety during the study (presence and reports of AE's/ SAE's),
- Verify accuracy/completeness of entries into eCRF,
- Matching data entries from the eCRF with source data in the patient record (Source Data Verification, SDV),
- Assessment of study progress,
- Is the study conducted according to protocol and GCP guidelines?
- Discussion with the examiner about the further conduct of the study as well as deficiencies found.

The monitor's access to study records is therefore assured by the investigators at the testing sites.

11.1.2 Audits/ Inspections

The test sites are audited by the sponsor, if applicable.

Internal and external audits can be carried out to guarantee that the test is carried out in accordance with the GCP guidelines. In addition, inspections by authorities can be carried out in accordance with the German Medicines Act. The auditor/inspector is independent of the employees involved in the audit.

During the audit, the following points, among others, are checked:

- Conducting the test according to the protocol
- Data validity
- Quality of the examination according to GCP guidelines

After each audit, the sponsor receives an audit report by the auditor.
This must be kept with the other documents relevant to the audit in order to have it available in the event of an inspection by the authorities. At the end of the audit, an audit certificate is attached to the final report.

12 Data entry and data management

The medical data of the patients collected in the study are recorded using electronic data entry forms (Case Report Forms - eCRF). For this purpose, the study software secuTrial® of the company interActive Systems GmbH (iAS) is used.

The study software fulfills the regulatory requirements (according to GCP, FDA 21 CFR Part 11) and includes, among other things, audit trail, electronic signature, programmable plausibility, consistency and

Translated version of the German trial protocol

Value range checks, query management system for online monitoring and a customizable role and authorization system.

The eCRF is created in collaboration with the study director, the sponsor's representative and the KKS Charité. The eCRF will only be released for data entry after extensive functional testing, validation and subsequent acceptance by the study director.

12.1 Data security

The study database is protected by an authentication procedure. Only authorized study personnel with personal identification have access to the study database. Individual authorization levels or roles (investigator, study nurse, study director, etc.) are always defined for each study. This makes it possible to implement different modes of processing the study data, for example, to release the forms (partial data) for editing, for reading or for data control (review).

Access to the study database is created and managed by the KKS Charité. The establishment of new study sites and accesses for study personnel is done in coordination with the study management or headquarters.

The personal access data must be stored securely using a suitable procedure and kept secret in order to prevent unauthorized use by third parties. Furthermore, the personal access data may not be passed on to enable others to use the study database.

The student database can be accessed via the Internet at any time. To use it, Internet access and a computer with a standard Internet browser are required.

The study data are recorded online and transferred directly to the study database. Local data storage does not take place. The data transfer between the local computer and the study database takes place via a secure connection (SSL encryption), so that the transferred study data cannot be read or manipulated.

To ensure data security, the study data is backed up by daily backups of the study database. In addition, the availability of the study server is guaranteed by using the professional server environment of the IT center of the Charité.

12.2 Privacy

A concept for pseudonymization of study data is implemented in the study software. This ensures the strict separation of identifying data (IDAT) and medical data (MDAT) of the patients.

The medical data are sent to the study database without any personally identifiable information and are stored there. The identifying data, on the other hand, are printed out at the study center and remain there. The link between the IDAT and MDAT can only be made via the key, also called pseudonym.

Translated version of the German trial protocol

The secuTrial® study software automatically generates a pseudonym for each patient, consisting of a combination of six alphanumeric characters (three letters and three numbers). All study data are then only linked to the pseudonym in the study database.

12.3 Data quality

To ensure the quality of the study data, the monitor performs data checks during the course of the study. This involves comparing the data stored in the study database with the source data at the test center.

After the end of data entry and completion of monitoring (closing all queries), the study director initiates the locking of the database.

The data can then be exported from the study database and processed in the further data management process. Further data checks are carried out using SAS programs. If the data checks reveal missing or implausible data, data queries are sent to the relevant study centers for clarification.

For subsequent data correction, (1.) the database accesses can be reactivated, or (2.) the data correction can also be performed during the data management process via documented script in SAS. After correction and completion of all data, the study data will be
"closed".

12.4 Data transfer

The actual study data are prepared during the data management process and compiled in vertical and/or horizontalized form so that they are immediately usable for statistical analysis.

Data formats and storage media are to be agreed for the data transfer. The data can be transferred in SAS, SPSS or CSV format and will be sent either by mail on CD/DVD or electronically with signed e-mail and data encryption.

Translated version of the German trial protocol

12.5 Generation of the pseudonym

The patient's pseudonym is assigned by the eCRF used when the patient is "created".

13 Statistical analysis

The statistical evaluation will be performed under the guidance of the study statistician Prof. Dr. Tim Friede (Institute of Medical Statistics, University Medical Center Göttingen). The planned statistical evaluations are described below. Details of the analyses will be regulated in a separate document, the Statistical Analysis Plan (SAP), which will be finalized at the latest before database lock/unblinding.

13.1 Case Count Estimate

Primary endpoint is the change in MADRS from week 1 to week 7. Assuming a standardized mean difference (Cohen's d) of 0.5, a t-test at the one-sided significance level of 2.5% with a case number of 64 patients per group has a power of 80%. After adjusting for approximately 20% dropout, we plan to recruit 80 patients per group (total case number 160 patients). These assumptions are supported by observations in a study by Bauer et al. (2009) with a similar design [50]. Case number planning was performed using nQuery Advisor 7.0.

13.2 Randomization

Patient allocation to the treatment arms is based on a central randomization code generated by the study statistician, Professor Dr. Tim Friede (University Medical Center Göttingen). Randomization will be block-wise, stratified by center to achieve balanced allocation. The randomization list will be sent directly to the pharmacy by the study statistician. The pharmacy (Clinical Pharmacist Cornelia Eberhardt, Charité- Universitätsmedizin Berlin, Campus-Virchow-Klinikum, Pharmacy, Department of Clinical Investigational Medicinal Products) will be instructed on how to perform the proper allocation concealment. Based on the randomization code, the pharmacy will centrally provide each center in the series with the sequentially numbered, tamper evident containers that do not differ in weight or appearance. In addition, an audit trail will be established by writing the subjects' pseudonyms on the empty containers.

13.3 Statistical evaluation

13.3.1 Hypotheses

Primarily, the null hypothesis H0: $\Delta \leq 0$ is tested against the alternative $_{H1}$: $\Delta > 0$ at the significance level $\alpha = 2.5\%$, where Δ is the difference in the expected values of the differences between week 7 and week 1 of the MADRS summary scores.

Secondarily, the null hypotheses that there is no treatment group difference in the secondary endpoints will be tested. These hypothesis tests are also performed at the one-sided significance level of $\alpha = 2.5\%$.

Translated version of the German trial protocol

13.3.2 Definition of the evaluation population

The primary evaluation population is the Intention-to-treat Population. The evaluation populations are defined as follows:

Intent-to-treat population

- all randomized patients (as randomized)
- With at least one use of investigational medication and at least one MADRS on therapy.

Per-protocol population

- Patients who received the entire therapy (6 weeks) according to the protocol
- who could be followed up at least until the end of therapy

Population for safety analysis

- all patients who have received at least one dose of the investigational drug

13.3.3 Evaluation of primary and secondary target parameters Primary target parameter

The primary endpoint of changes in MADRS sum scores from week 1 to week 7 is evaluated by a linear mixed model repeated measures (MMRM) model [51]. Treatment group, study center, measurement time point, and MADRS sum score at baseline enter as fixed effects; patient-specific effects enter the model as a random effect with normal distribution and expected value 0. Least squares estimates of differences from week 1 to week 7 for the two treatment groups as well as for differences between the two treatment groups are reported with 95% confidence intervals and p value for no group difference.

Although these MMRM evaluations are robust against dropouts to a certain extent, further supporting evaluations are performed in case of substantial dropout. In particular, models are used that are suitable for modeling informative dropouts. These include so-called shared random effects models [52]. This will help to assess the sensitivity of the primary evaluation to dropout.

Secondary target parameters

Evaluation of the other scales such as CGI-S, BDI, HAMD-17, and SCL-90, as well as the TMT A and B, will be performed analogously to the evaluation described above for MADRS. The secondary endpoints of remission and response to treatment will be evaluated using logistic regressions, with dropouts as no remission and no response to treatment. Treatment and center are included in the logistic regression model as factors and MADRS sum score at baseline as a covariate. Treatment effects are reported as adjusted odds ratios with 95% confidence intervals and p-values for the null hypothesis that the odds ratio equals 1. Secondary outcome parameters are tested exploratory and not confirmatory, therefore p-values are not adjusted for multiple testing.

Exploratory subgroup analyses

Data are described stratified by sex. All analyses will also be repeated with regard to possible sex differences with sex and interaction between sex and treatment as further factors. In addition, we will test whether patients with elevated peripheral inflammatory markers at baseline benefit to a greater extent from therapy. For this purpose, the peripheral inflammatory markers and their interactions with treatment will be included individually in the regression models described above.

Translated version of the German trial protocol

13.3.4 Safety evaluation

The frequencies (percentages) of adverse AEs and SAEs and of occurrence and severity of treatment-related adverse events are reported for the treatment groups. Kaplan-Meyer curves of times from initiation of therapy to occurrence of the event with 95% confidence intervals are provided for selected events. P values of log-rank tests are reported descriptively. In addition, descriptive statistics (e.g., mean, standard deviation, minimum, maximum) will be calculated for clinically significant changes in vital signs, weight, and blood parameters, and data will be graphically processed in an appropriate form (e.g., box plots) for both treatment groups, and group comparisons will be performed (e.g., Wilcoxon rank sum test).

13.3.5 Potential interim evaluations and endpoints for early dropout.

No interim evaluations are planned.

14 Reporting

14.1 Biometric report

The statistical analysis and preparation of the biometric report is performed by the study statistician Prof. Dr. Tim Friede with support from his institute and in collaboration with the sponsor and the principal investigator. All information contained in this report is confidential.

14.2 Final Report

The preparation of an integrated final report follows the requirements of ICH E3: Structure and Contents of Clinical Study Reports.

Upon completion of the biometric evaluation, an integrated report will be generated by Sponsor.

The report includes the clinical report, statistical report, single value tables and conclusions.

It is signed by the study director, sponsor, biometrician, monitor, data manager, and center directors, if applicable.

14.3 Publication

Publication of study results will occur regardless of how the results turn out.

15 Ethical, legal and administrative aspects

15.1 Vote of the Ethics Committee (according to AMG § 42 (1) and GCP-V § 7)

Protocol, patient information and informed consent will be submitted to the responsible ethics committee (here: the lead ethics committee: Ethics Committee of the State of Berlin, Fehrbelliner Platz 1, 10707 Berlin, as well as the participating ethics committees) for review. The study will only be started after receipt of the consenting evaluation and the availability of the approval of the competent higher federal authority.

The ethics committee will be informed immediately about all changes in the protocol (according to GCP-V § 10) and about all events that could affect the safety of the patients. Furthermore, the Ethics Committee will be informed about all suspected cases of unexpected serious adverse events that have become known to the sponsor, as well as about the regular or premature termination of the study.

Translated version of the German trial protocol

Investigators are required to register with the ethics committee responsible for them (submission of proof of qualification) before enrolling patients in the study. It is necessary to inform the ethics committee about protocol changes (according to GCP-V § 10).

15.2 Approval of the higher federal authority (according to AMG § 42 (2) and GCP-V § 7)

The study is submitted to the competent higher federal authority (here: BfArM, Federal Institute for Drugs and Medical Devices, Clinical Trial Registry, Kurt-Georg-Kiesinger-Allee 3, 53175 Bonn, Germany) for approval. The study will not be started until this approval and the consenting assessment of the responsible ethics committee has been received.

15.3 Notification to the state authorities (according to AMG § 67)

The performance of this study will be reported to the competent state authorities of the testing agencies (acc. § 67 AMG). The sponsor and all investigators must be named there (for notification of a clinical trial in accordance with Section 67 (1) and (3) AMG and Section 12 (1-3) GCP-V to the competent state authority, the supervisory authorities have developed a uniform form that is used for this purpose (see https://www.zlg.de/arzneimittel/service/dokumente.html, retrieval date: 29.04.2015).

15.4 Patient information and consent form

Patient education

Prior to enrollment in the study (e.g., randomization), each patient will be informed orally and in writing by the attending physician about the nature, objectives, anticipated benefits, and potential risks of the study.

Consent to study participation

Each patient must give written consent to participate in the study. The patient must be given sufficient time and opportunity to decide on his or her participation and clarify any open questions before study measures are initiated.

The declaration of consent is signed and personally dated by the patient and the attending physician. If the patient is capable of giving consent but is unable to sign it personally, a witness must confirm the verbal explanation by signing it.

15.5 Proband insurance

For this present clinical study, an insurance policy (according to AMG § 40 paragraph 1 sentence 3 No. 8) has been taken out.

Insurance coverage for the study is provided by HDI-Gerling Versicherung AG Germany (Am Schönenkamp 45, 40599 Düsseldorf, Germany; policy number: 57 010326 03017; application number: 16002015113; insurance file: 2330 33 1600 / 1502). (The scope of the sum insured per patient is up to a maximum of 500,000 euros.

15.6 Privacy

Patients are informed that their disease-related data are stored in pseudonymized form and used for scientific evaluations (publications, registration dossiers). Patients have the right to be informed about the stored data. They will also be informed that their pseudonymized data will be passed on to the competent higher federal authorities, including those in member states of the European Union in which the study is being conducted, as well as to the competent ethics committees, within the framework of the statutory reporting obligations for drug safety. Patients who do not consent to this disclosure may not participate in the study.

Translated version of the German trial protocol

15.7 Data retention and access

The originals of all central study documents including documentation sheets are stored at the study center (at study management/sponsor) for at least 10 years after completion of the clinical trial (GCP-V § 13(10)).

The investigator/principal investigator retains the accrued administrative documents (correspondence with ethics committee, monitoring authority, study management, study center), the signed informed consent forms, copies of the documentation sheets and the general study documentation (protocol, amendments) for the above mentioned time.

Original study patient data (medical records) must be retained in accordance with the archival period applicable to the study sites (the investigators), but not less than 10 years.

The patient identification list must be kept for 15 years according to Directive 2001/83/EC.

Translated version of the German trial protocol

16 Bibliography

[1] H. A. WHITEFORD, L. DEGENHARDT, J. REHM, A.J. BAXTER, A.J. FERRARI, H.E. ERSKINE, F.J. CHARLSON, R.E. NORMAN, A.D. FLAXMAN, N. JOHNS, R. BURSTEIN, C.J. MURRAY, T. VOS: Global burden of disease attributable to mental and substance use disorders: findings from the Global Burden of Disease Study 2010. In: *Lancet* 382 (2013), pp. 1575-1586.

[2] M.A. BUSCH, U.E. MASKE, L. RYL, R. SCHLACK, U. HAPKE: [Prevalence of depressive symptoms and diagnosed depression among adults in Germany: results of the German Health Interview and Examination Survey for Adults (DEGS1)]. In: *Bundesgesundheitsblatt Gesundheitsforschung Gesundheitsschutz* 56 (2013), pp. 733-739.

[3] A. J. RUSH, M.H. TRIVEDI, S.R. WISNIEWSKI, A.A. NIERENBERG, J.W. STEWART, D. WARDEN, G. NIEDEREHE, M.E. THASE, P.W. LAVORI, B.D. LEBOWITZ, P.J. MCGRATH, J.F. ROSENBAUM, H.A. SACKEIM, D.J. KUPFER, J. LUTHER, M. FAVA: Acute and longer-term outcomes in depressed outpatients requiring one or several treatment steps: a STAR*D report. In: *Am J Psychiatry* 163 (2006), pp. 1905-1917.

[4] M. FAVA: Diagnosis and definition of treatment-resistant depression. In: *Biol. Psychiatry* 53 (2003), pp. 649-659.

[5] N. VOGELZANGS, H.E. DUIVIS, A.T. BEEKMAN, C. KLUFT, J. NEUTEBOOM, W. HOOGENDIJK, J.H. SMIT, P. DE JONGE, B.W. PENNINX: Association of depressive disorders, depression characteristics and antidepressant medication with inflammation. In: *Transl Psychiatry* 2 (2012), p. e79.

[6] A. H. MILLER, V. MALETIC, C.L. RAISON: Inflammation and its discontents: the role of cytokines in the pathophysiology of major depression. In: *Biol Psychiatry* 65 (2009), pp. 732- 741.

[7] C.L. RAISON, L. CAPURON, A.H. MILLER: Cytokines sing the blues: inflammation and the pathogenesis of depression. In: *Trends in immunology* 27 (2006), pp. 24-31.

[8] M.B. HOWREN, D.M. LAMKIN, J. SULS: Associations of depression with C-reactive protein, IL-1, and IL-6: a meta-analysis. In: *Psychosom Med* 71 (2009), pp. 171-186.

[9] S. LANQUILLON, J.C. KRIEG, U. BENING-ABU-SHACH, H. VEDDER: Cytokine production. and treatment response in major depressive disorder. In: *Neuropsychopharmacology* 22 (2000), pp. 370-379.

[10] R. UHER, K.E. TANSEY, T. DEW, W. MAIER, O. MORS, J. HAUSER, M.Z. DERNOVSEK, N. HENIGSBERG, D. SOUERY, A. FARMER, P. MCGUFFIN: An inflammatory biomarker as a differential predictor of outcome of depression treatment with escitalopram and nortriptyline. In: *Am J Psychiatry* 171 (2014), pp. 1278-1286.

[11] G. Z. REUS, G.R. FRIES, L. STERTZ, M. BADAWY, I.C. PASSOS, T. BARICHELLO, F. KAPCZINSKI, J. QUEVEDO: The role of inflammation and microglial activation in the pathophysiology of psychiatric disorders. In: *Neuroscience* 300 (2015), pp. 141-154.

[12] E. SETIAWAN, A.A. WILSON, R. MIZRAHI, P.M. RUSJAN, L. MILER, G. RAJKOWSKA, I. SURIDJAN, J.L. KENNEDY, P.V. REKKAS, S. HOULE, J.H. MEYER: Role of translocator protein density, a marker of neuroinflammation, in the brain during major depressive episodes. In: *JAMA Psychiatry* 72 (2015), pp. 268-275.

[13] S.G. TORRES-PLATAS, C. CRUCEANU, G.G. CHEN, G. TURECKI, N. MECHAWAR: Evidence for increased microglial priming and macrophage recruitment in the dorsal anterior

Translated version of the German trial protocol

cingulate white matter of depressed suicides. In: *Brain Behav Immun* 42 (2014), pp. 50-59.
[14] J. YRJANHEIKKI, T. TIKKA, R. KEINANEN, G. GOLDSTEINS, P.H. CHAN, J. KOISTINAHO: A tetracycline derivative, minocycline, reduces inflammation and protects against focal

Translated version of the German trial protocol

cerebral ischemia with a wide therapeutic window. In: *Proc Natl Acad Sci U S A* 96 (1999), pp. 13496-13500.
[15] L.J. JIN, F. SCHLESINGER, Q. GUAN, Y.P. SONG, Z.Y. NIE: The two different effects of the potential neuroprotective compound minocycline on AMPA-type glutamate receptors. In: *Pharmacology* 89 (2012), pp. 156-162.
[16] H. SAKATA, K. NIIZUMA, H. YOSHIOKA, G.S. KIM, J.E. JUNG, M. KATSU, P. NARASIMHAN, C.M. MAIER, Y. NISHIYAMA, P.H. CHAN: Minocycline-preconditioned neural stem cells enhance neuroprotection after ischemic stroke in rats. In: *J Neurosci* 32 (2012), pp. 3462-3473.
[17] D. ZEMKE, A. MAJID: The potential of minocycline for neuroprotection in human neurologic disease. In: *Clin Neuropharmacol* 27 (2004), pp. 293-298.
[18] T.M. TIKKA, J.E. KOISTINAHO: Minocycline provides neuroprotection against N-methyl-D-aspartate neurotoxicity by inhibiting microglia. In: *Journal of Immunology* 166 (2001), pp. 7527-7533.
[19] Y. YANG, V.M. SALAYANDIA, J.F. THOMPSON, L.Y. YANG, E.Y.. ESTRADA, Y. YANG: Attenuation of acute stroke injury in rat brain by minocycline promotes blood-brain barrier remodeling and alternative microglia/macrophage activation during recovery. In: *Journal of neuroinflammation* 12 (2015), p. 26.
[20] Y. SEKI, T.A. KATO, A. MONJI, Y. MIZOGUCHI, H. HORIKAWA, M. SATO-KASAI, D. YOSHIGA, S. KANBA: Pretreatment of aripiprazole and minocycline, but not haloperidol, suppresses oligodendrocyte damage from interferon-gamma-stimulated microglia in co- culture model. In: *Schizophr Res* 151 (2013), pp. 20-28.
[21] T.Y. YUNE, J.Y. LEE, G.Y. JUNG, S.J. KIM, M.H. JIANG, Y.C. KIM, Y.J. OH, G.J. MARKELONIS, T.H. OH: Minocycline alleviates death of oligodendrocytes by inhibiting pro-nerve growth factor production in microglia after spinal cord injury. In: *J Neurosci* 27 (2007), S. 7751-7761.
[22] T.M. TIKKA, J.E. KOISTINAHO: Minocycline provides neuroprotection against N-methyl-D-aspartate neurotoxicity by inhibiting microglia. In: *J Immunol* 166 (2001), pp. 7527- 7533.
[23] J. HELLMANN-REGEN, I. HERZOG, N. FISCHER, I. HEUSER, F. REGEN: Do tetracyclines.
and erythromycin exert anti-acne effects by inhibition of P450-mediated degradation of retinoic acid? In: *Exp Dermatol* 23 (2014), pp. 290-293.
[24] H.M. ABELAIRA, G.Z. REUS, J. QUEVEDO: Animal models as tools to study the pathophysiology of depression. In: *Revista brasileira de psiquiatria (Sao Paulo, Brazil : 1999)* 35 Suppl 2 (2013), pp. S112-120.
[25] M. MOLINA-HERNANDEZ, N.P. TELLEZ-ALCANTARA, J. PEREZ-GARCIA, J.I. OLIVERA-. LOPEZ, M.T. JARAMILLO-JAIMES: Antidepressant-like actions of minocycline combined with several glutamate antagonists. In: *Prog Neuropsychopharmacol Biol Psychiatry* 32 (2008), pp. 380-386.
[26] M. MOLINA-HERNANDEZ, N.P. TELLEZ-ALCANTARA, J. PEREZ-GARCIA, J.I. OLIVERA-. LOPEZ, M.T. JARAMILLO-JAIMES: Desipramine or glutamate antagonists synergized the antidepressant-like actions of intra-nucleus accumbens infusions of minocycline in male Wistar rats. In: *Prog Neuropsychopharmacol Biol Psychiatry* 32 (2008), pp. 1660-1666.
[27] S. ARAKAWA, Y. SHIRAYAMA, Y. FUJITA, T. ISHIMA, M. HORIO, K. MUNEOKA, M. IYO, K. HASHIMOTO: Minocycline produced antidepressant-like effects on the learned helplessness rats with alterations in levels of monoamine in the amygdala and no changes in BDNF levels in the hippocampus at baseline. In: *Pharmacol Biochem Behav* 100 (2012), pp. 601-606.

Translated version of the German trial protocol

[28] L.S. ZHENG, N. KANEKO, K. SAWAMOTO: Minocycline treatment ameliorates interferon-alpha-induced neurogenic defects and depression-like behaviors in mice. In: *Frontiers in cellular neuroscience* 9 (2015), p. 5.
[29] J.C. O'CONNOR, M.A. LAWSON, C. ANDRE, M. MOREAU, J. LESTAGE, N. CASTANON, K.W. KELLEY, R. DANTZER: Lipopolysaccharide-induced depressive-like behavior is mediated by indoleamine 2,3-dioxygenase activation in mice. In: *Mol Psychiatry* 14 (2009), pp. 511-522.
[30] U. MEYER, M.J. SCHWARZ, N. MULLER: Inflammatory processes in schizophrenia: a promising neuroimmunological target for the treatment of negative/cognitive symptoms and beyond. In: *Pharmacol Ther* 132 (2011), pp. 96-110.
[31] I.B. CHAUDHRY, J. HALLAK, N. HUSAIN, F. MINHAS, J. STIRLING, P. RICHARDSON, S. DURSUN, G. DUNN, B. DEAKIN: Minocycline benefits negative symptoms in early schizophrenia: a randomised double-blind placebo-controlled clinical trial in patients on standard treatment. In: *J Psychopharmacol* 26 (2012), pp. 1185-1193.
[32] Y. LEVKOVITZ, S. MENDLOVICH, S. RIWKES, Y. BRAW, H. LEVKOVITCH-VERBIN, G. GAL, S. FENNIG, I. TREVES, S. KRON: A double-blind, randomized study of minocycline for the treatment of negative and cognitive symptoms in early-phase schizophrenia. In: *J Clin Psychiatry* 71 (2010), pp. 138-149.
[33] M.R. KHODAIE-ARDAKANI, O. MIRSHAFIEE, M. FAROKHNIA, M. TAJDINI, S.M. HOSSEINI, A. MODABBERNIA, F. REZAEI, B. SALEHI, H. YEKEHTAZ, M. ASHRAFI, M. TABRIZI,
S. AKHONDZADEH: Minocycline add-on to risperidone for treatment of negative symptoms in patients with stable schizophrenia: randomized double-blind placebo-controlled study. In: *Psychiatry Res* 215 (2014), pp. 540-546.
[34] D.L. KELLY, K.M. SULLIVAN, J.P. MCEVOY, R.P. MCMAHON, H.J. WEHRING, J.M. GOLD, F. LIU, D. WARFEL, G. VYAS, C.M. RICHARDSON, B.A. FISCHER, W.R. KELLER, M.M. KOOLA, S.M. FELDMAN, J.C. RUSS, R.S. KEEFE, J. OSING, L. HUBZIN, S. AUGUST, T.M.
WALKER, R.W. BUCHANAN: Adjunctive Minocycline in Clozapine-Treated Schizophrenia Patients With Persistent Symptoms. In: *J Clin Psychopharmacol* 35 (2015), pp. 374-381.
[35] K. OYA, T. KISHI, N. IWATA: Efficacy and tolerability of minocycline augmentation therapy in schizophrenia: a systematic review and meta-analysis of randomized controlled trials. In: *Hum Psychopharmacol* 29 (2014), pp. 483-491.
[36] T. MIYAOKA, R. WAKE, M. FURUYA, K. LIAURY, M. IEDA, K. KAWAKAMI, K. TSUCHIE,
M. TAKI, K. ISHIHARA, T. ARAKI, J. HORIGUCHI: Minocycline as adjunctive therapy for patients with unipolar psychotic depression: an open-label study. In: *Prog Neuropsychopharmacol Biol Psychiatry* 37 (2012), pp. 222-226.
[37] J. SAVITZ, S. PRESKORN, T.K. TEAGUE, D. DREVETS, W. YATES, W. DREVETS: Minocycline and aspirin in the treatment of bipolar depression: a protocol for a proof-of-concept, randomised, double-blind, placebo-controlled, 2x2 clinical trial. In: *BMJ Open* 2 (2012), pp. e000643.
[38] M.M. OLIVIA MAY DEAN, MELANIE ASHTON, LESLEY BERK, BURANEE KANCHANATAWAN, ATAPOL SUGHONDHABIROM, SOOKJAREON TANGWONGCHAI, CHEE NG,
NATHAN DOWLING, GIN S. MALHI, MICHAEL BERK: Protocol and Rationale-The Efficacy of Minocycline as an Adjunctive Treatment for Major Depressive Disorder: A Double Blind, Randomised, Placebo Controlled Trial. In: *Clin Psychopharmacol Neuroscience* 2014 Dec; 12(3) (2014), pp. 180-188. .

Translated version of the German trial protocol

[39] M. BAUER, M. ADLI, C. BAETHGE, A. BERGHOFER, J. SASSE, A. HEINZ, T. BSCHOR: Lithium augmentation therapy in refractory depression: clinical evidence and neurobiological mechanisms. In: *Canadian journal of psychiatry. Revue canadienne de psychiatrie* 48 (2003), S. 440-448.

Translated version of the German trial protocol

[40] M. BAUER, S. DOPFMER: Lithium augmentation in treatment-resistant depression: meta-analysis of placebo-controlled studies. In: *J Clin Psychopharmacol* 19 (1999), pp. 427- 434.
[41] P.H. GORDON, D.H. MOORE, R.G. MILLER, J.M. FLORENCE, J.L. VERHEIJDE, C. DOORISH, J.F. HILTON, G.M. SPITALNY, R.B. MACARTHUR, H. MITSUMOTO, H.E. NEVILLE, K. BOYLAN, T. MOZAFFAR, J.M. BELSH, J. RAVITS, R.S. BEDLACK, M.C. GRAVES, L.F. MCCLUSKEY, R.J. BAROHN, R. TANDAN: Efficacy of minocycline in patients with amyotrophic lateral sclerosis: a phase III randomised trial. In: *Lancet Neurol* 6 (2007), pp. 1045-1053.
[42] S.A. MONTGOMERY, M. ASBERG: A new depression scale designed to be sensitive to change. In: *Br J Psychiatry* 134 (1979), pp. 382-389.
[43] ASSOCIATION FOR METHODOLOGY AND DOCUMENTATION IN PSYCHIATRY (AMDP) AND COLLEGIUM INTERNATIONALE PSYCHIATRIEÁE SCALARUM (CIPS) (eds.) *Ratingscales. For Psychiatry.* 1990.
[44] G. BONDOLFI, F. JERMANN, B.W. ROUGET, M. GEX-FABRY, A. MCQUILLAN, A. DUPONT-WILLEMIN, J.M. AUBRY, C. NGUYEN: Self- and clinician-rated Montgomery-Asberg Depression Rating Scale: evaluation in clinical practice. In: *J Affect Disord* 121 (2010), pp.
268-272.
[45] World Medical Association Declaration of Helsinki: ethical principles for medical research involving human subjects. In: *JAMA* 310 (2013), pp. 2191-2194.
[46] S. LEUCHT, H. FENNEMA, R. ENGEL, M. KASPERS-JANSSEN, P. LEPPING, A. SZEGEDI: What does the HAMD mean? In: *Journal of Affective Disorders* 148 (2013), pp. 243-248.
[47] M. W. ENNS, B.J. COX, J.D.A. PARKER, J.E. GUERTIN: Confirmatory factor analysis of the Beck Anxiety and Depression Inventories in patients with major depression. In: *Journal of Affective Disorders* 47 (1998), pp. 195-200.
[48] L.R. DEROGATIS, K. RICKELS, A.F. ROCK: The SCL-90 and the MMPI: a step in the validation of a new self-report scale. In: *Br J Psychiatry* 128 (1976), pp. 280-289.
[49] S. WAGNER, I. HELMREICH, N. DAHMEN, K. LIEB, A. TADIC: Reliability of three alternate forms of the trail making tests a and B. In: *Arch Clin Neuropsychol* 26 (2011), pp. 314-321.
[50] M. BAUER, H.W. PRETORIUS, E.L. CONSTANT, W.R. EARLEY, J. SZAMOSI, M. BRECHER: Extended-release quetiapine as adjunct to an antidepressant in patients with major depressive disorder: results of a randomized, placebo-controlled, double-blind study. In: *J Clin Psychiatry* 70 (2009), pp. 540-549.
[51] C.H. MALLINCKRODT, S.W. CLARK, R.J. CARROLL, G. MOLENBERGHS: Assessing response profiles from incomplete longitudinal clinical trial data under regulatory considerations. In: *J Biopharm Stat* 13 (2003), pp. 179-190.
[52] R. HENDERSON, P. DIGGLE, A. DOBSON: Joint modeling of longitudinal measurements and event time data. In: *Biostatistics* 1 (2000), pp. 465-480.

Translated version of the German trial protocol
