# Supplementary material for: Effect of Minocycline on Depressive Symptoms in Patients With Treatment-Resistant Depression: A Randomized Clinical Trial
Source: JAMA Netw Open. 2022 Sep 14;5(9):e2230367. doi: 10.1001/jamanetworkopen.2022.30367 (PMC9475381; doi:10.1001/jamanetworkopen.2022.30367)
Supplement: Supplement 2. — eTable 1. Adverse Events eTable 2. Exploratory Endpoints eTable 3. Exploratory Subgroup Analysis eFigure 1. CGI eFigure 2. Boxplot HAMD-17 eFigure 3. BDI eFigure 4. SCL-90 eFigure 5. TMT-A/B eFigure 6. Distribution of Remission by Assignment eFigure 7. Distribution of Response by Assignment eFigure 8. CRP eFigure 9. Leukocytes eFigure 10. Gender eFigure 11. MADRS at Baseline eFigure 12. CRP eFigure 13. BMI eFigure 14. CTQ [file jamanetwopen-e2230367-s002.pdf]

# Supplemental Online Content

Hellmann-Regen J, Clemens V, Grözinger M, et al. Effect of minocycline on depressive symptoms in patients with treatment-resistant depression: a randomized clinical trial. *JAMA Netw Open.* 2022;5(9):e2230367. doi:10.1001/jamanetworkopen.2022.30367

**eTable 1.** Adverse Events

**eTable 2.** Exploratory Endpoints

**eTable 3.** Exploratory Subgroup Analysis

**eFigure 1.** CGI

**eFigure 2.** Boxplot HAMD-17

**eFigure 3.** BDI

**eFigure 4.** SCL-90

**eFigure 5.** TMT-A/B

**eFigure 6.** Distribution of Remission by Assignment

**eFigure 7.** Distribution of Response by Assignment

**eFigure 8.** CRP

**eFigure 9.** Leukocytes

**eFigure 10.** Gender

**eFigure 11.** MADRS at Baseline

**eFigure 12.** CRP

**eFigure 13.** BMI

**eFigure 14.** CTQ

This supplemental material has been provided by the authors to give readers additional information about their work.

## eTable 1. Adverse Events

| Adverse events | Minocycline (n=81) | | Placebo (n=87) | | p-value |
|---|---|---|---|---|---|
| | N | (%) | N | (%) | |
| Number of subjects with at least one AE | 68 | (84.0) | 63 | (72.4) | 0.106 |
| Number of subjects with at least two AE | 56 | (69.1) | 45 | (41.7) | 0.032 |
| Number of subjects with at least one sAE | 6 | (7.4) | 7 | (8.0) | 0.987 |
| Total number of AEs | 265 | (52.8) | 237 | (47.2) | 0.211 |
| Total number of sAEs | 6* | (2.3) | 7* | (3.0) | 0.838 |
| Severity of AE | | | | | 0.021 |
| Mild | 194 | (73.2) | 147 | (62.0) | |
| Moderate | 65 | (24.5) | 79 | (33.3) | |
| Severe | 6 | (2.3) | 11 | (4.6) | |
| Relation to study medication? | | | | | 0.145 |
| Yes | 27 | (10.2) | 21 | (8.9) | |
| No | 53 | (20.0) | 65 | (27.4) | |
| Unknown | 185 | (69.8) | 151 | (63.7) | |
| Severity of sAE | | | | | 0.065 |
| Mild | 0 | (0.0) | 2 | (28.6) | |
| Moderate | 3 | (50.0) | 0 | (0.0) | |
| Severe | 3 | (50.0) | 5 | (71.4) | |
| Leads to permanent or serious disability or invalidity | 1 | (16.7) | 0 | (0.0) | |
| Leads to inpatient treatment or extends it | 5 | (83.3) | 6 | (85.7) | |
| Fatal or life threatening | 0 | (0.0) | 1** | (14.3) | |

| Type of (S)AE | | |
|---|---|---|
| | Minocycline | Placebo |
| Number of (S)AEs | 265 | 237 |
| | n (%) | n (%) |
| Headache | 33 (11.9) | 43 (16.5) |
| Flu-like symptoms | 30 (10.8) | 17 ( 6.5) |
| Dyspepsia/indigestion | 25 ( 9.0) | 19 ( 7.3) |
| Flatulence and diarrhea | 15 ( 5.4) | 26 (10.0) |
| Skin problems | 22 ( 7.9) | 15 ( 5.7) |
| Dizziness | 20 ( 7.2) | 7 ( 2.7) |
| Nausea | 12 ( 4.3) | 12 ( 4.6) |
| Psychiatric symptoms, exacerbation | 10 ( 3.6) | 10 ( 3.8) |
| Tiredness | 11 ( 4.0) | 9 ( 3.4) |
| Blood parameters changes | 12 ( 4.3) | 6 ( 2.3) |
| Insomnia | 4 ( 1.4) | 9 ( 3.4) |
| Other neurological symptoms | 10 ( 3.6) | 4 ( 1.5) |
| General pain/joint pain | 8 ( 2.9) | 5 ( 1.9) |
| Hyperhidrosis | 4 ( 1.4) | 6 ( 2.3) |
| Back pain | 4 ( 1.4) | 6 ( 2.3) |

© 2022 Hellmann-Regen J et al. *JAMA Network Open.*

| **Type of (S)AE** | | |
|---|---|---|
| | **Minocycline** | **Placebo** |
| **Fungal infection** | **5 ( 1.8)** | **1 ( 0.4)** |
| **Teeth problems** | **3 ( 1.1)** | **3 ( 1.1)** |
| **Sore throat/cold** | **2 ( 0.7)** | **3 ( 1.1)** |
| **Visual impairments** | **3 ( 1.1)** | **1 ( 0.4)** |
| **Tinnitus** | **3 ( 1.1)** | **1 ( 0.4)** |
| **Urological problems** | **3 ( 1.1)** | **1 ( 0.4)** |
| **Chest palpitation/pain** | **0 ( 0.0)** | **3 ( 1.1)** |
| **Hypertension** | **2 ( 0.7)** | **1 ( 0.4)** |
| **Constipation** | **0 ( 0.0)** | **3 ( 1.1)** |
| **Irritable cough** | **0 ( 0.0)** | **2 ( 0.8)** |
| **Insect bites** | **1 ( 0.4)** | **1 ( 0.4)** |
| **Increased appetite** | **0 ( 0.0)** | **2 ( 0.8)** |
| **Drowsiness** | **2 ( 0.7)** | **0 ( 0.0)** |
| **Allergic conjunctivitis, intermittent** | **1 ( 0.4)** | **0 ( 0.0)** |
| **Allergic reaction** | **1 ( 0.4)** | **0 ( 0.0)** |
| **Head impact with sloping roof frontal left side** | **0 ( 0.0)** | **1 ( 0.4)** |
| **Rear-end collision** | **0 ( 0.0)** | **1 ( 0.4)** |
| **Biliary pancreatitis with choledocholithiasis** | **0 ( 0.0)** | **1 ( 0.4)** |
| **Blood spotting in stool, fresh reddish blood, a.e. due to injuries in anal canal** | **0 ( 0.0)** | **1 ( 0.4)** |
| **Cholecystits, cholecystectomy** | **1 ( 0.4)** | **0 ( 0.0)** |
| **Colon carcinoma (incidental finding)** | **0 ( 0.0)** | **1 ( 0.4)** |
| **Intake of ibuprofen, novalgin, tilidine** | **1 ( 0.4)** | **0 ( 0.0)** |
| **Panic attack** | **1 ( 0.4)** | **0 ( 0.0)** |
| **Somewhat increased symptoms of pre-existing asthma** | **1 ( 0.4)** | **0 ( 0.0)** |
| **Folliculitus** | **1 ( 0.4)** | **0 ( 0.0)** |
| **Stye on left eye** | **0 ( 0.0)** | **1 ( 0.4)** |
| **Facial swelling of unknown origin** | **1 ( 0.4)** | **0 ( 0.0)** |
| **Granulopenia** | **1 ( 0.4)** | **0 ( 0.0)** |
| **Hoarseness** | **1 ( 0.4)** | **0 ( 0.0)** |
| **Light blood in stool** | **1 ( 0.4)** | **0 ( 0.0)** |
| **Herpes labialis** | **0 ( 0.0)** | **1 ( 0.4)** |
| **Itching and burning in the vagina** | **1 ( 0.4)** | **0 ( 0.0)** |
| **Itching of both eyes** | **0 ( 0.0)** | **1 ( 0.4)** |
| **Small laceration on the head during gardening** | **0 ( 0.0)** | **1 ( 0.4)** |
| **Lump on the wrist** | **1 ( 0.4)** | **0 ( 0.0)** |
| **Concentration problems** | **1 ( 0.4)** | **0 ( 0.0)** |
| **Temporary visual perception problems (duration 15 min)** | **1 ( 0.4)** | **0 ( 0.0)** |
| **Eyelid swelling on the right** | **0 ( 0.0)** | **1 ( 0.4)** |
| **Lymphoma** | **1 ( 0.4)** | **0 ( 0.0)** |
| **Metallic taste in the mouth** | **1 ( 0.4)** | **0 ( 0.0)** |
| **Nosebleed** | **0 ( 0.0)** | **1 ( 0.4)** |
| **Norovirus infection** | **1 ( 0.4)** | **0 ( 0.0)** |
| **Sleep apnea syndrome** | **0 ( 0.0)** | **1 ( 0.4)** |
| **Swelling of the right eyelid** | **0 ( 0.0)** | **1 ( 0.4)** |
| **Vision problems with dry eyes** | **0 ( 0.0)** | **1 ( 0.4)** |
| **Stenting of femoral artery in case of known PAOD** | **0 ( 0.0)** | **1 ( 0.4)** |
| **Symptomatic cholecystolithiasis** | **0 ( 0.0)** | **1 ( 0.4)** |
| **Tendinits of the right long biceps tendon** | **0 ( 0.0)** | **1 ( 0.4)** |
| **Tendovaginits of the right wrist** | **1 ( 0.4)** | **0 ( 0.0)** |
| **Type of (S)AE** | | |

© 2022 Hellmann-Regen J et al. *JAMA Network Open.*

| | Minocycline | Placebo |
|---|---|---|
| **Bicycle accident with subsequent hospitalization for 1 night with fracture of zygomatic bone** | **1 ( 0.4)** | **0 ( 0.0)** |
| **Varicose vein surgery** | **1 ( 0.4)** | **0 ( 0.0)** |
| **Whitish tongue coating** | **0 ( 0.0)** | **1 ( 0.4)** |
| **Recurrence of menstruation, under constant use of an oral contraceptive** | **0 ( 0.0)** | **1 ( 0.4)** |
| **Tick bite** | **0 ( 0.0)** | **1 ( 0.4)** |
| **Cerebral aneurysm hemorrhage** | **0 ( 0.0)** | **1 ( 0.4)** |

**eTable 1** Adverse Events

* no relation to study medication

** incidental finding

© 2022 Hellmann-Regen J et al. *JAMA Network Open.*

## eTable 2. Exploratory Endpoints

| End points | Pre-treatment mean (SD) | | Post-treatment mean (SD) | | Difference of week 6 and baseline between Placebo and Minocycline (95% CI) | | | p-values |
|---|---|---|---|---|---|---|---|---|
| | Minocycline | Placebo | Minocycline | Placebo | | | | |
| **Anhedonia** | 20.2 (4.3) N=81 | 20.4 (4.4) N=87 | 12.8 (6.5) N=69 | 13.2 (7.6) N=75 | 0.76 (-1.34 to 2.86) | | | 0.48 |
| **Sickness behavior** | 8.8 (1.8) N=81 | 9.0 (2.2) N=87 | 6.2 (2.7) N=69 | 6.2 (3.1) N=75 | -0.005 (-0.85 to 0.84) | | | 0.99 |
| **Fear, suicidality** | 6.1 (1.5) N=81 | 6.1 (1.7) N=87 | 4.2 (2.0) N=69 | 4.2 (2.2) N=75 | -0.01 (-0.62 to 0.60) | | | 0.98 |
| **HAMD6** | 11.4 (2.2) N=81 | 11.4 (2.1) N=87 | 7.4 (3.5) N=69 | 7.6 (6.7) N=71 | 0.33 (-0.77 to 1.42) | | | 0.56 |

**eTable 2** Exploratory outcome measures. The difference between the two treatment groups are based on the least square differences for the change of the outcome measures from baseline to week 6 based on linear mixed effects models for repeated measures.

© 2022 Hellmann-Regen J et al. *JAMA Network Open.*

## eTable 3. Exploratory Subgroup Analysis

| | Subgroup | Pre-treatment mean (SD) for MADRS | | Post-treatment mean (SD) For MADRS | | Difference of week 6 and baseline between Placebo and Minocycline | 95% CI | | p-values |
|---|---|---|---|---|---|---|---|---|---|
| | | Minocycline | Placebo | Minocycline | Placebo | | | | |
| **Gender** | **Male** | 27.0 (4.9) N=50 | 26.4 (4.7) N=39 | 19.7 (7.6) N=42 | 18.8 (8.3) N=31 | 0.76 (-2.70 to 4.22) | | | 0.6642 |
| | **Female** | 25.4 (4.7) N=31 | 26.8 (5.4) N=48 | 14.4 (7.3) N=27 | 15.3 (9.5) N=44 | 3.21 (-0.48 to 6.90) | | | 0.0877 |
| **CTQ sum score** | **0** | 26.8 (6.0) N=32 | 26.5 (5.0) N=39 | 16.3 (8.7) N=28 | 18. (9.9) N=32 | 2.55 (-.140 to 6.51) | | | 0.2041 |
| | **1** | 26.2 (4.1) N=18 | 25.8 (5.1) N=25 | 20.1 (8.3) N=15 | 19.7 (8.7) N=23 | 0.34 (-4.63 to 5.31) | | | 0.8937 |
| | **2** | 25.5 (4.7) N=13 | 27.5 (5.3) N=8 | 17.2 (7.0) N=11 | 17.6 (7.5) N=8 | -0.73 ( -7.95 to 6.50) | | | 0.8428 |
| | **3,4 or 5** | 26.9 (3.8) N=15 | 27.9 (5.4) N=15 | 17.7 (6.7) N=12 | 19.3 (8.7) N=12 | 2.36 (-3.73 to 8.45) | | | 0.7605 |
| **CTQ-Physical abuse** | **Yes** | 24.8 (4.3) N=12 | 25.6 (7.3) N=13 | 19.2 (4.6) N=10 | 18.5 (8.7) N=11 | -0.49 (-7.01 to 6.04) | | | 0.8833 |
| | **No** | 24.1 (5.4) N=68 | 25.1 (7.3) N=72 | 17.3 (8.3) N=58 | 18.8 (9.1) N=64 | 1.95 (-0.80 to 4.69) | | | 0.1635 |
| **CTQ-Emotional abuse** | **Yes** | 23.6 (4.2) N=31 | 25.3 (6.4) N=23 | 17.5 (6.5) N=25 | 18.9 (7.6) N=23 | 0.86 (-3.52 to 5.24) | | | 0.6976 |
| | **No** | 24.5 (5.8) N=49 | 25.1 (7.6) N=62 | 17.6 (8.7) N=43 | 18.7 (9.6) N=52 | 1.91 (-1.20 to 5.02) | | | 0.2273 |
| **CTQ-Sexual abuse** | **Yes** | 25.9 (3.6) N=12 | 27.4 (3.7) N=7 | 15.7 (8.3) N=10 | 16.6 (6.4) N=7 | 0.62 (-7.03 to 8.26) | | | 0.8737 |
| | **No** | 26.5 (5.1) N=67 | 26.6 (5.2) N=80 | 17.9 (7.9) N=57 | 19.0 (9.2) N=68 | 1.52 (-1.19 to 4.23) | | | 0.5752 |
| **CTQ-Emotional neglect** | **Yes** | 26.8 (4.0) N=36 | 26.9 (5.5) N=40 | 18.2 (7.3) N=30 | 19.4 (8.8) N=35 | 1.63 (-2.12 to 5.37) | | | 0.3924 |
| | **No** | 26.1 (5.6) N=43 | 26.4 (4.8) N=47 | 17.0 (8.5) N=37 | 18.2 (9.2) N=35 | 1.40 (-2.07 to 4.87) | | | 0.4272 |
| **CTQ-** | **Yes** | 25.9 (4.3) N=12 | 27.9 (5.6) N=14 | 19.2 (4.6) N=10 | 18.5 (8.7) N=11 | -0.49 (-7.01 to 6.04) | | | 0.8833 |

© 2022 Hellmann-Regen J et al. *JAMA Network Open.*

| | | | | | | | | | |
|---|---|---|---|---|---|---|---|---|---|
| **Physical neglect** | **No** | 26.5 (5.0) N=68 | 26.4 (5.0) N=73 | 17.3 (8.3) N=58 | 18.8 (9.1) N=64 | -0.30 (-0.80 to 4.69) | | | 0.1635 |
| **MADRS at baseline** | **Larger than the median value** | 30.7 (3.3) N=37 | 30.7 (3.5) N=44 | 20.5 (8.7) N=31 | 21.1 (8.8) N=39 | 1.77 (-1.91 to 5.44) | | | 0.3436 |
| | **Smaller than median value** | 22.8 (2.3) N=44 | 22.4 (2.3) N=43 | 15.3 (6.2) N=38 | 16.2 (8.7) N=36 | 1.22 (-2.28 to 4.73 ) | | | 0.4913 |
| **CRP at baseline** | **Larger than the median value** | 26.1 (5.0) N=47 | 26.4 (5.0) N=36 | 16.5 (8.6) N=40 | 16.3 (9.4) N=30 | 1.21 (-2.35 to 4.77) | | | 0.5033 |
| | **Smaller than median value** | 26.8 (4.6) N=34 | 26.9 (5.1) N=50 | 19.2 (6.6) N=29 | 20.4 (8.4) N=45 | 0.96 (-2.61 to 4.52) | | | 0.5967 |
| **BMI at baseline** | **Larger than the median value** | 26.3 (5.1) N=45 | 27.0 (5.2) N=40 | 18.2 (8.7) N=39 | 19.1 (8.6) N=40 | 0.68 (-2.87 To | | 4.23 | 0.7039 |
| | **Smaller than median value** | 26.5 (4.6) N=36 | 26.3 (5.0) N=47 | 16.9 (6.7) N=30 | 18.4 (9.4) N=35 | 2.28 | -.133 | 5.88 | 0.2149 |

**eTable 3** Exploratory subgroup analysis for the primary end point MADRS. MADRS = Montgomery Asberg Depression Rating Scale, BMI= body mass index, CTQ= Childhood Trauma Questionnaire. The presence of emotional, physical and sexual abuse as well as emotional and physical neglect are defined as at least moderate severity of abuse or neglect based on Häuser et al. (2011).The difference between the two treatment groups in each subgroup are based on the least square differences for the change of MADRS from baseline to week 6 based on linear mixed effects models for repeated measures.

**eFigure 1**. **CGI**

**A. Barplot CGI**

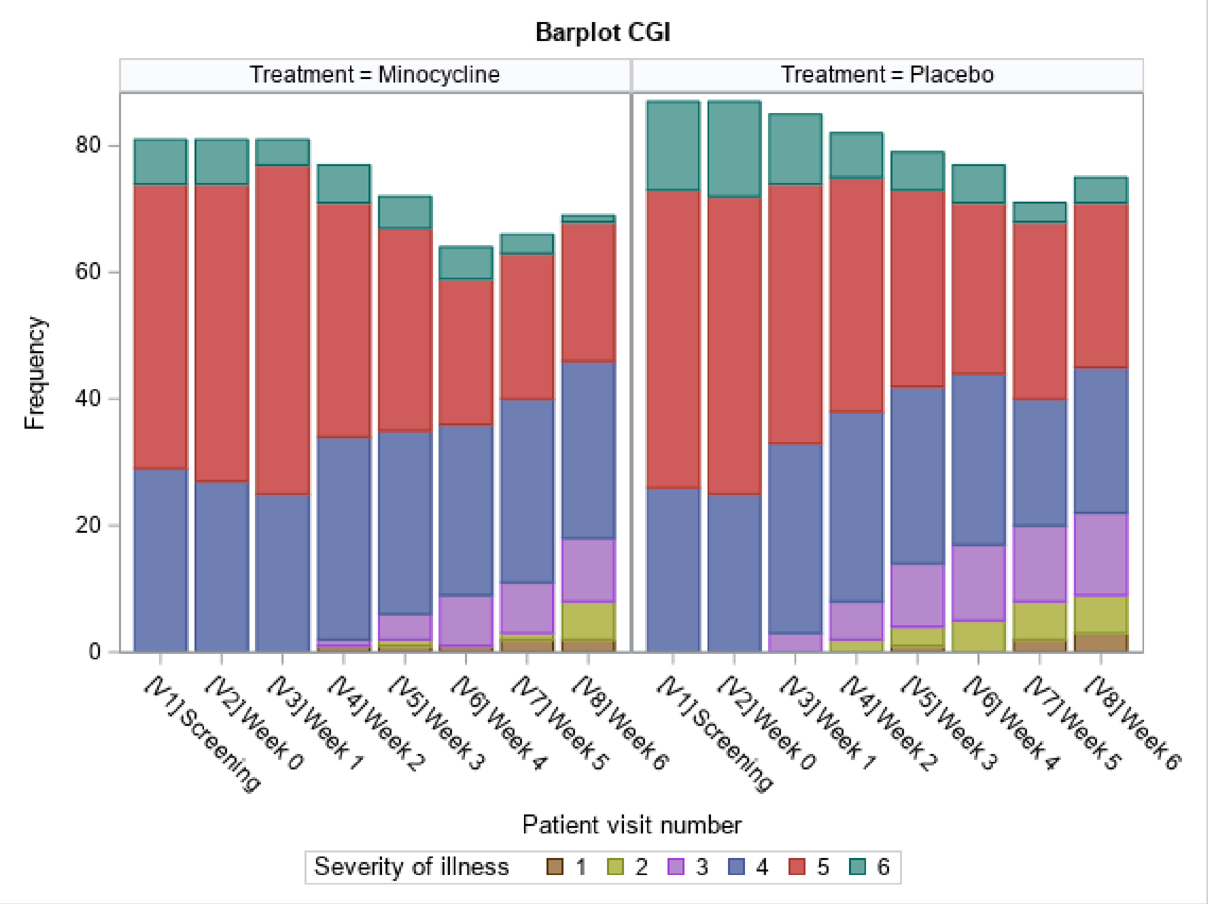


© 2022 Hellmann-Regen J et al. *JAMA Network Open.*

**B. Boxplot CGI**

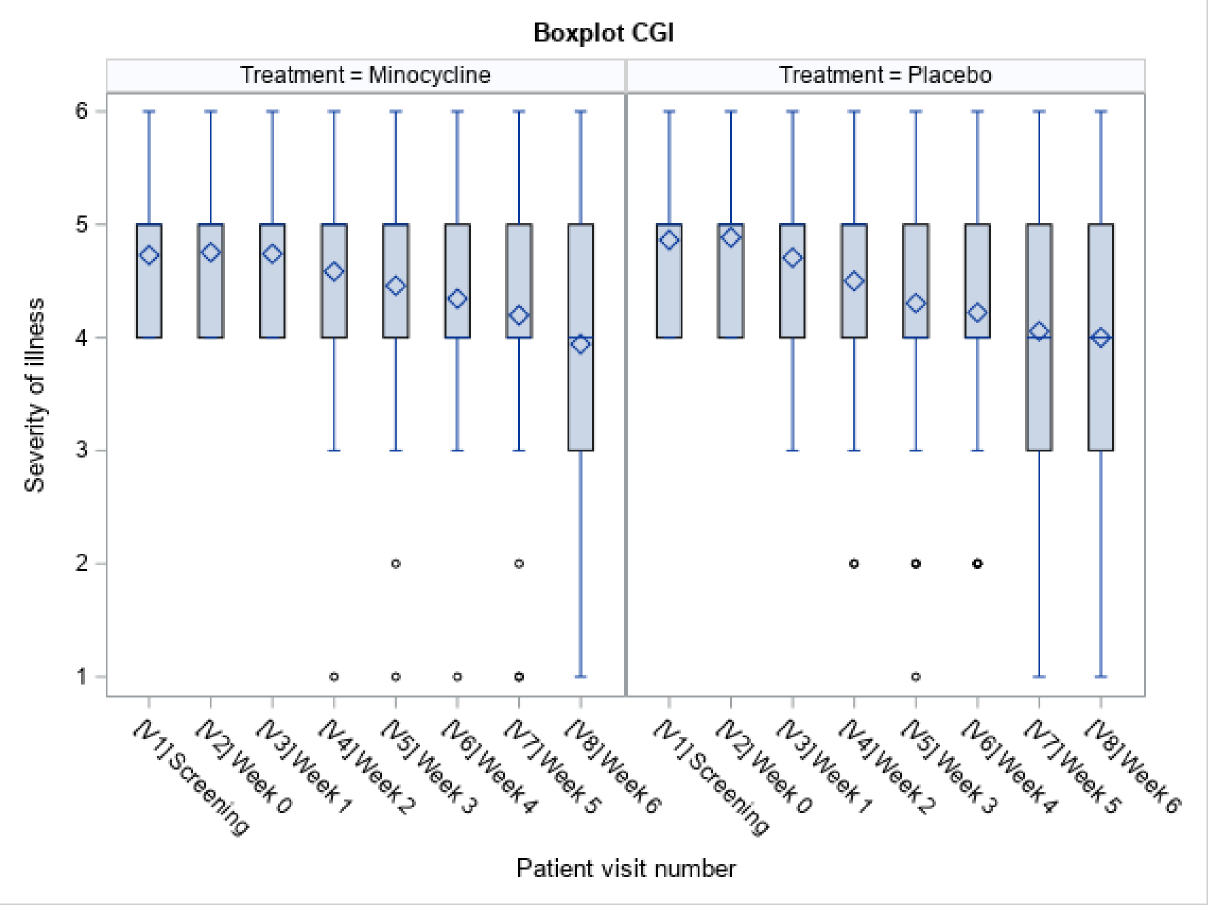


© 2022 Hellmann-Regen J et al. *JAMA Network Open.*

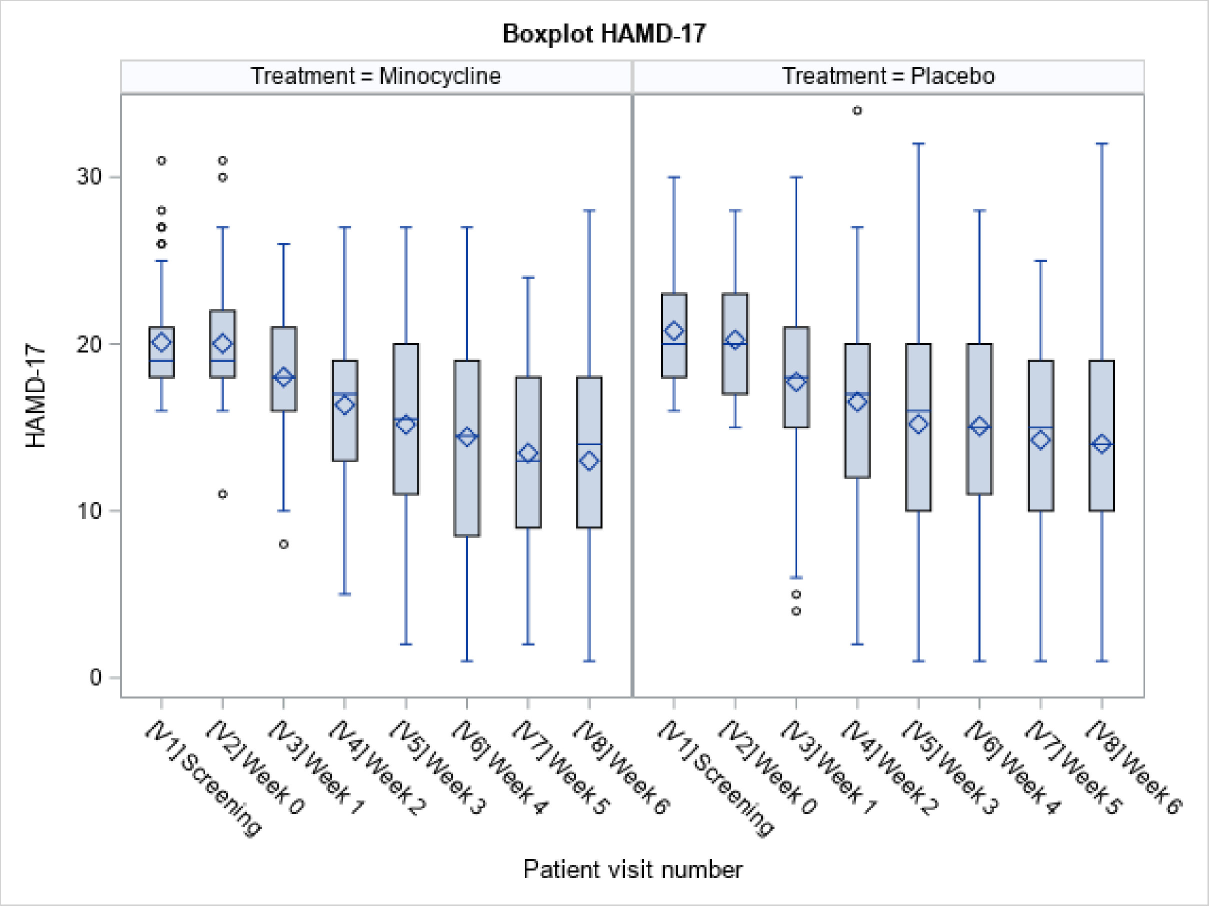


**eFigure 2. Boxplot HAMD-17**

© 2022 Hellmann-Regen J et al. *JAMA Network Open.*

**eFigure 3. BDI**

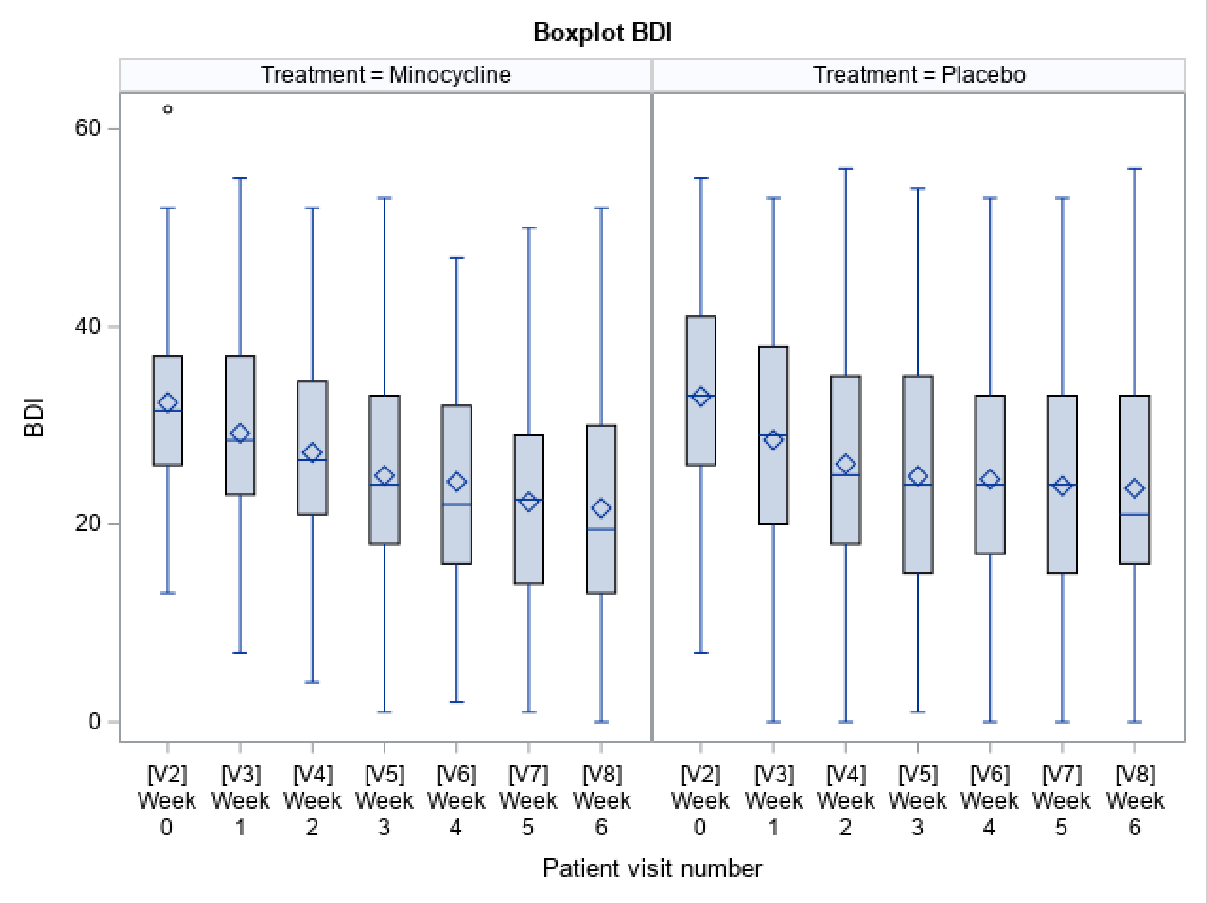


© 2022 Hellmann-Regen J et al. *JAMA Network Open.*

## eFigure 4. SCL-90

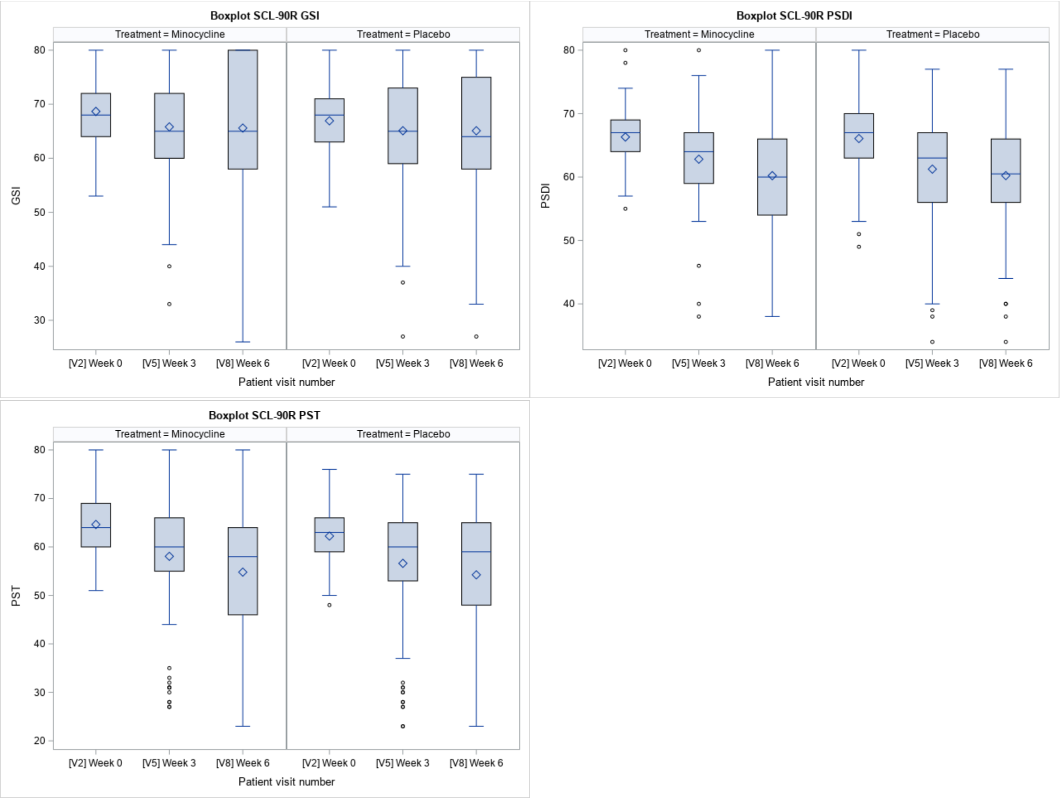


## eFigure 5. TMT-A/B

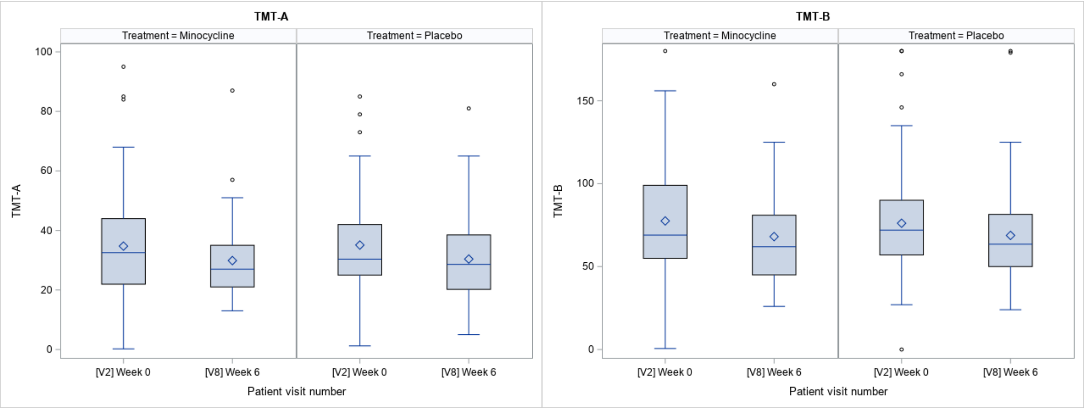


© 2022 Hellmann-Regen J et al. *JAMA Network Open.*

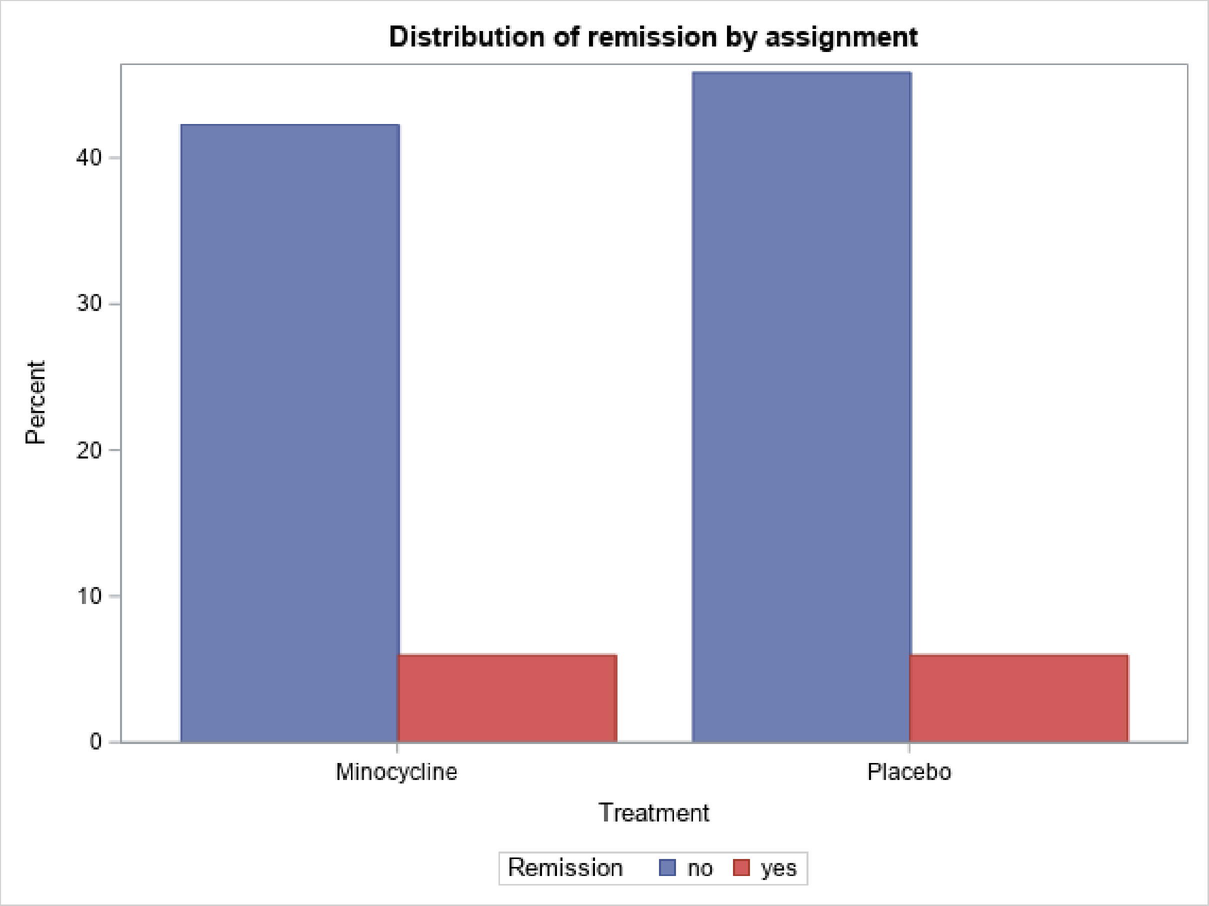


**eFigure 6. Distribution of Remission by Assignment**

© 2022 Hellmann-Regen J et al. *JAMA Network Open.*

**eFigure 7. Distribution of Response by Assignment**

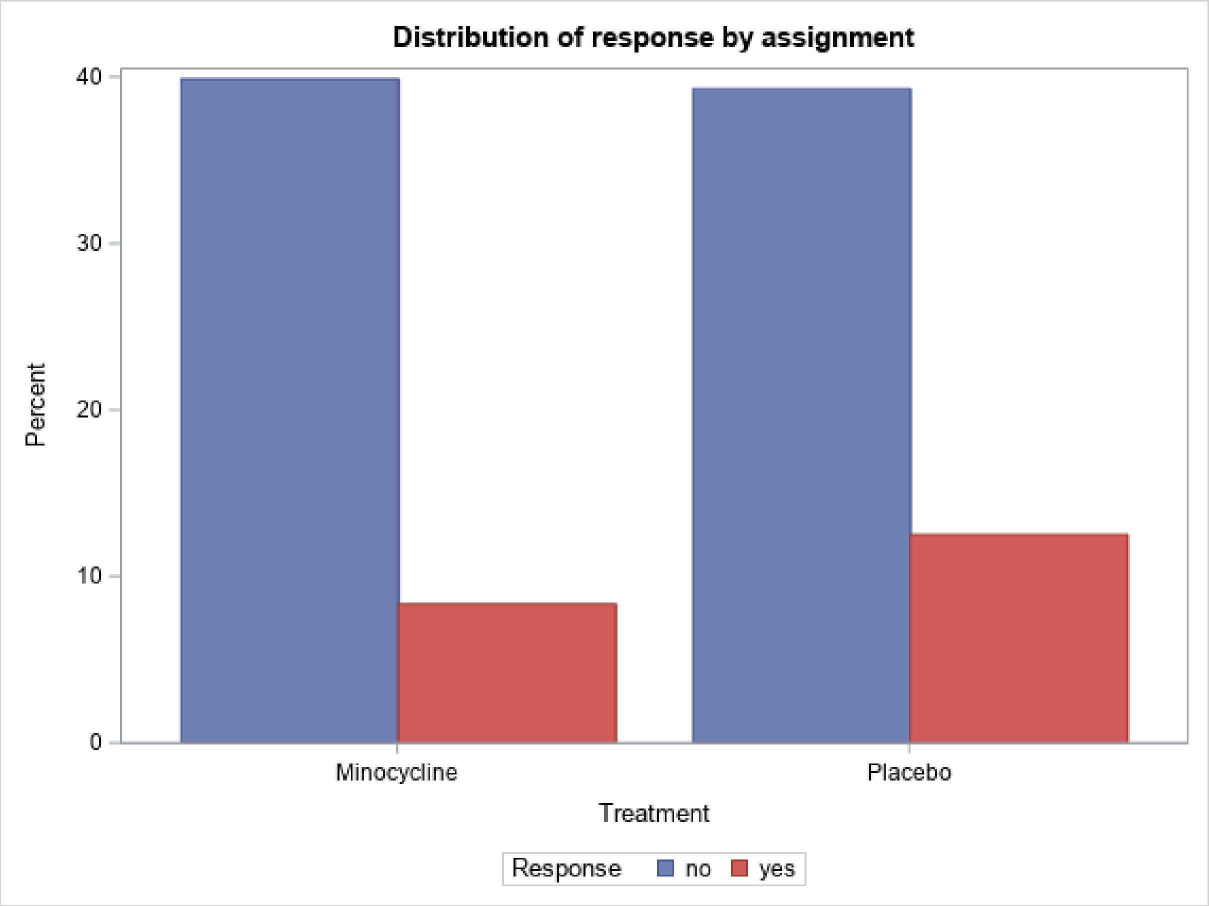

## Exploratory Outcomes

### eFigure 8. CRP

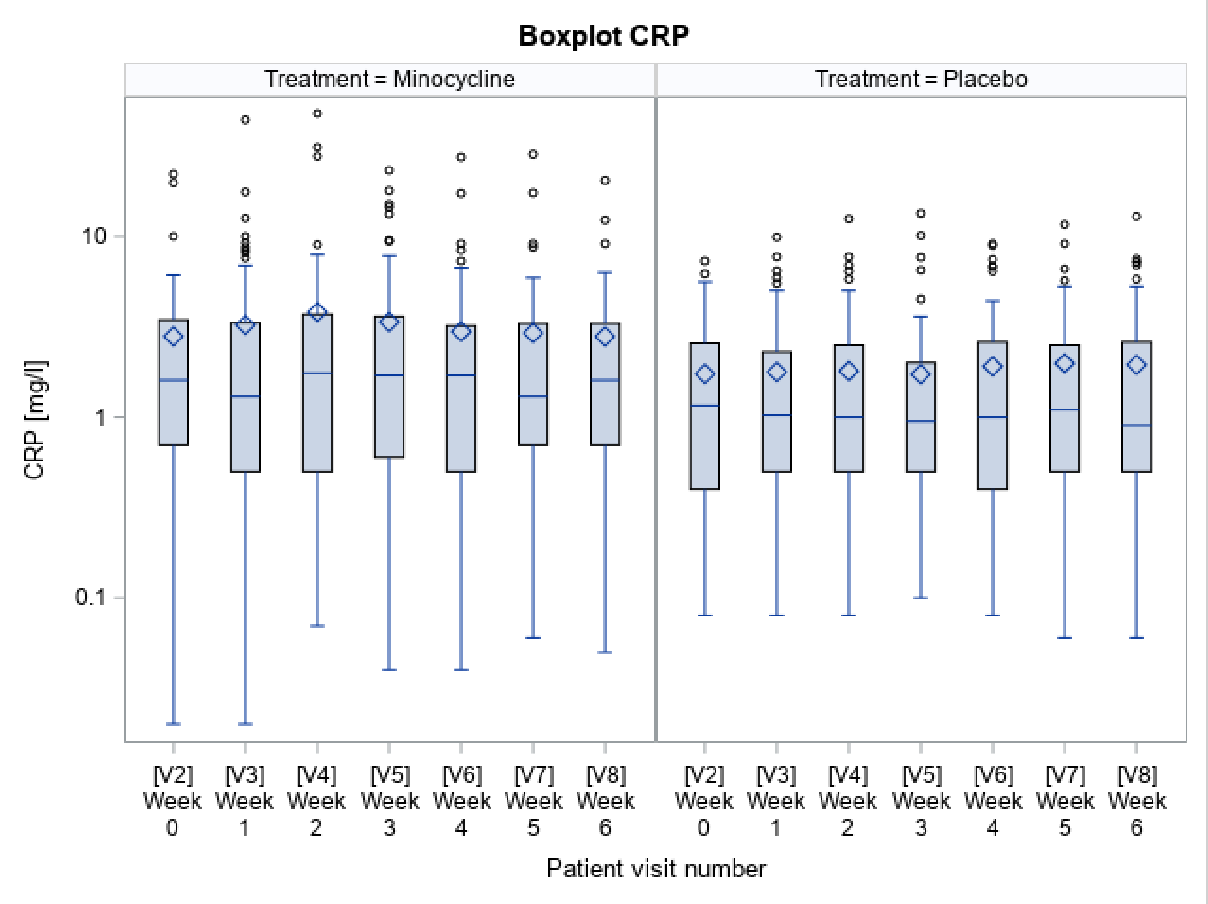


© 2022 Hellmann-Regen J et al. *JAMA Network Open.*

**eFigure 9. Leukocytes**

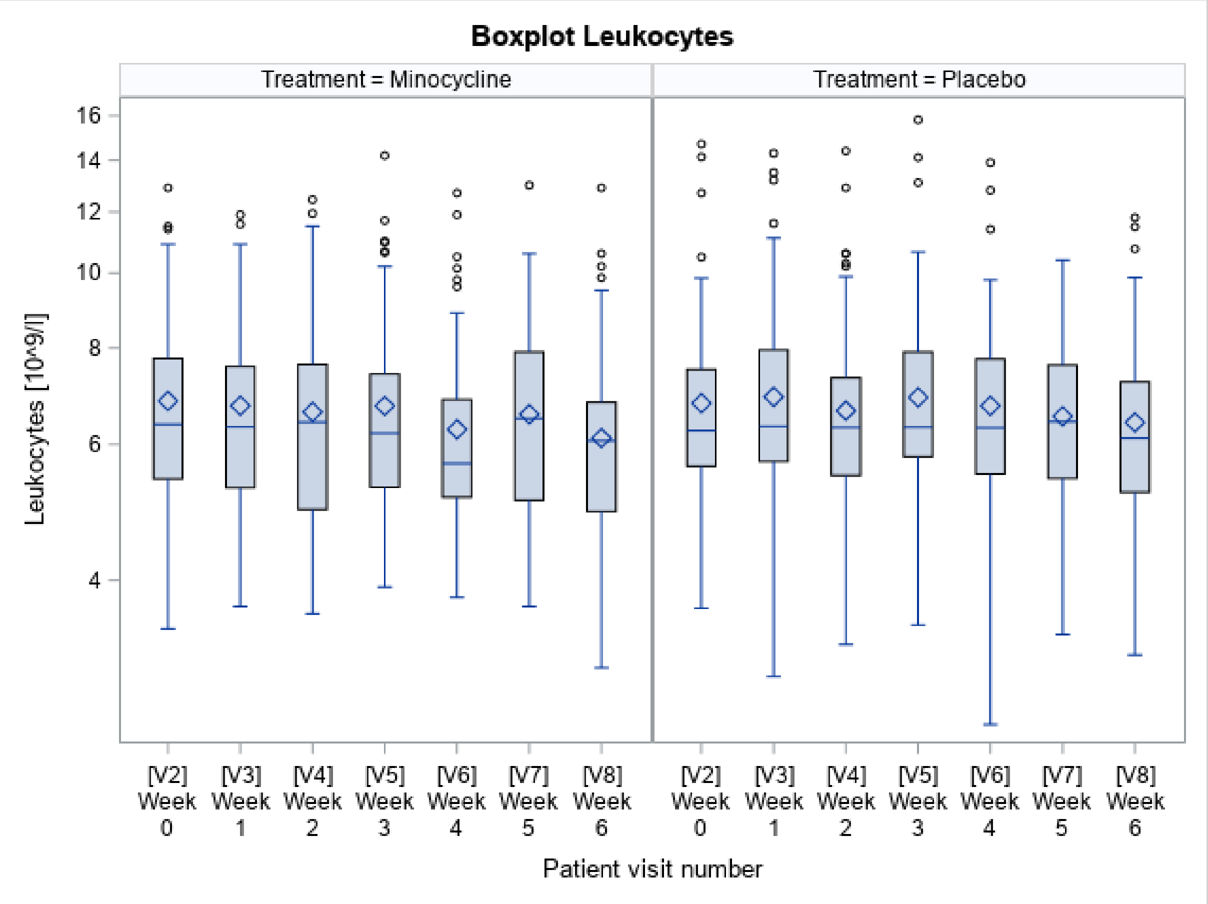


© 2022 Hellmann-Regen J et al. *JAMA Network Open.*

## Exploratory subgroup Analyses

### eFigure 10. Gender

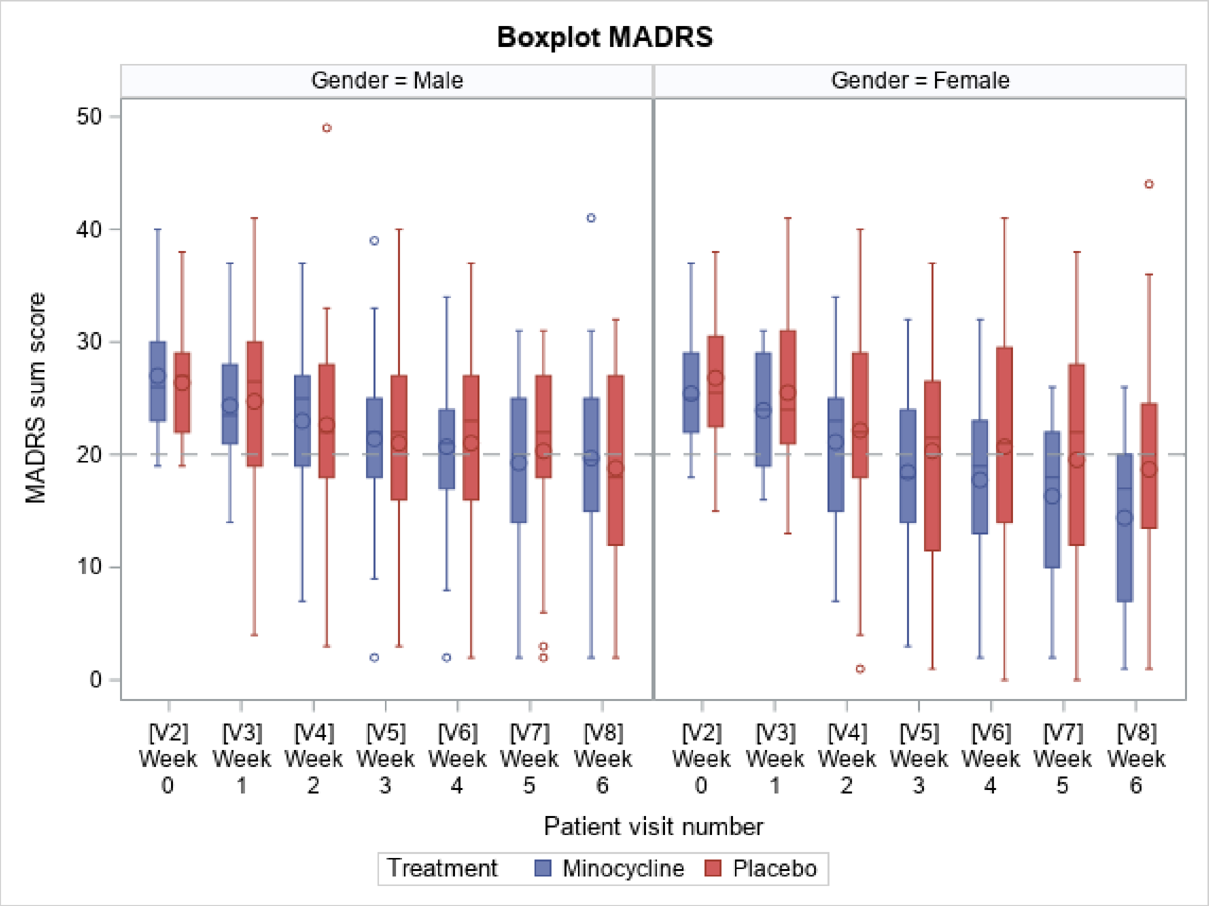


© 2022 Hellmann-Regen J et al. *JAMA Network Open.*

**eFigure 11. MADRS at Baseline**

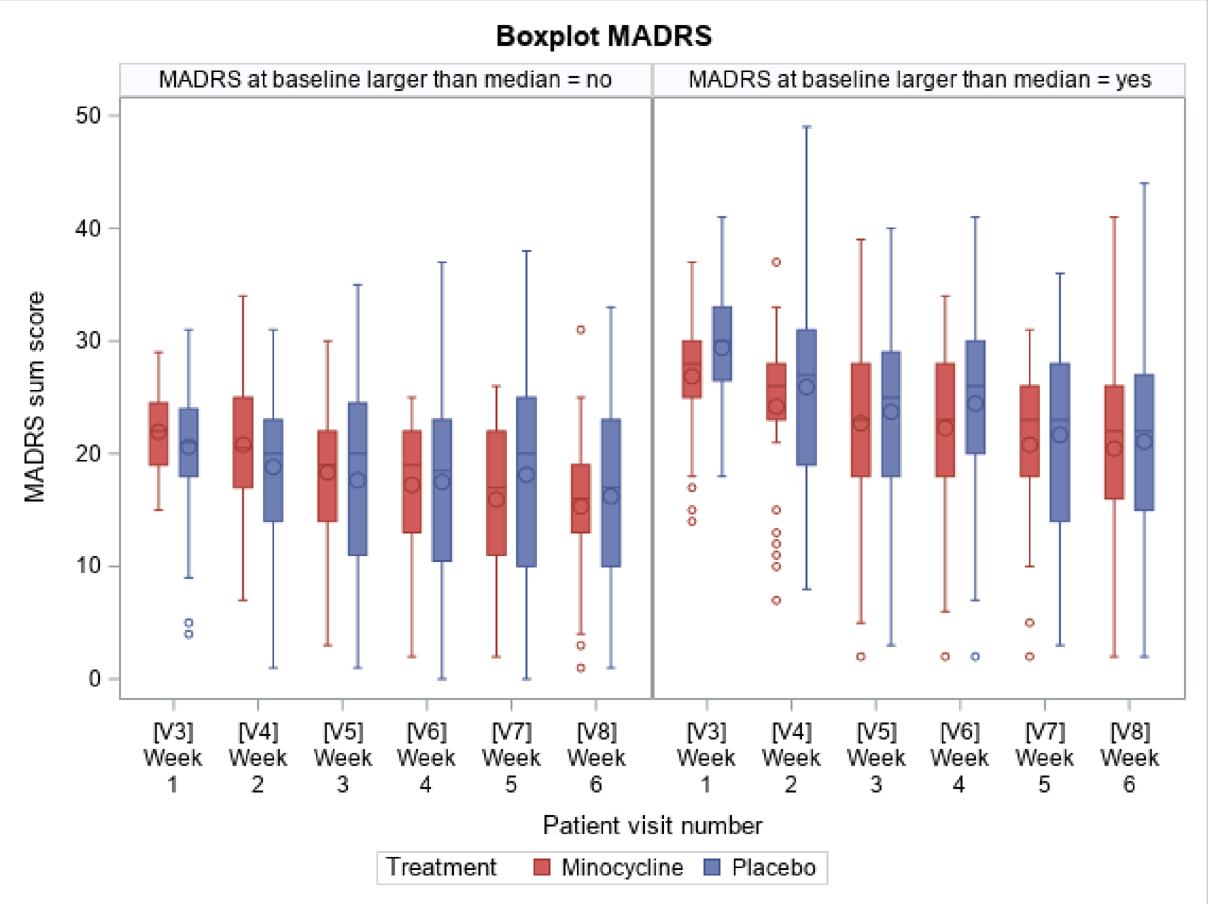


© 2022 Hellmann-Regen J et al. *JAMA Network Open.*

**eFigure 12. CRP**

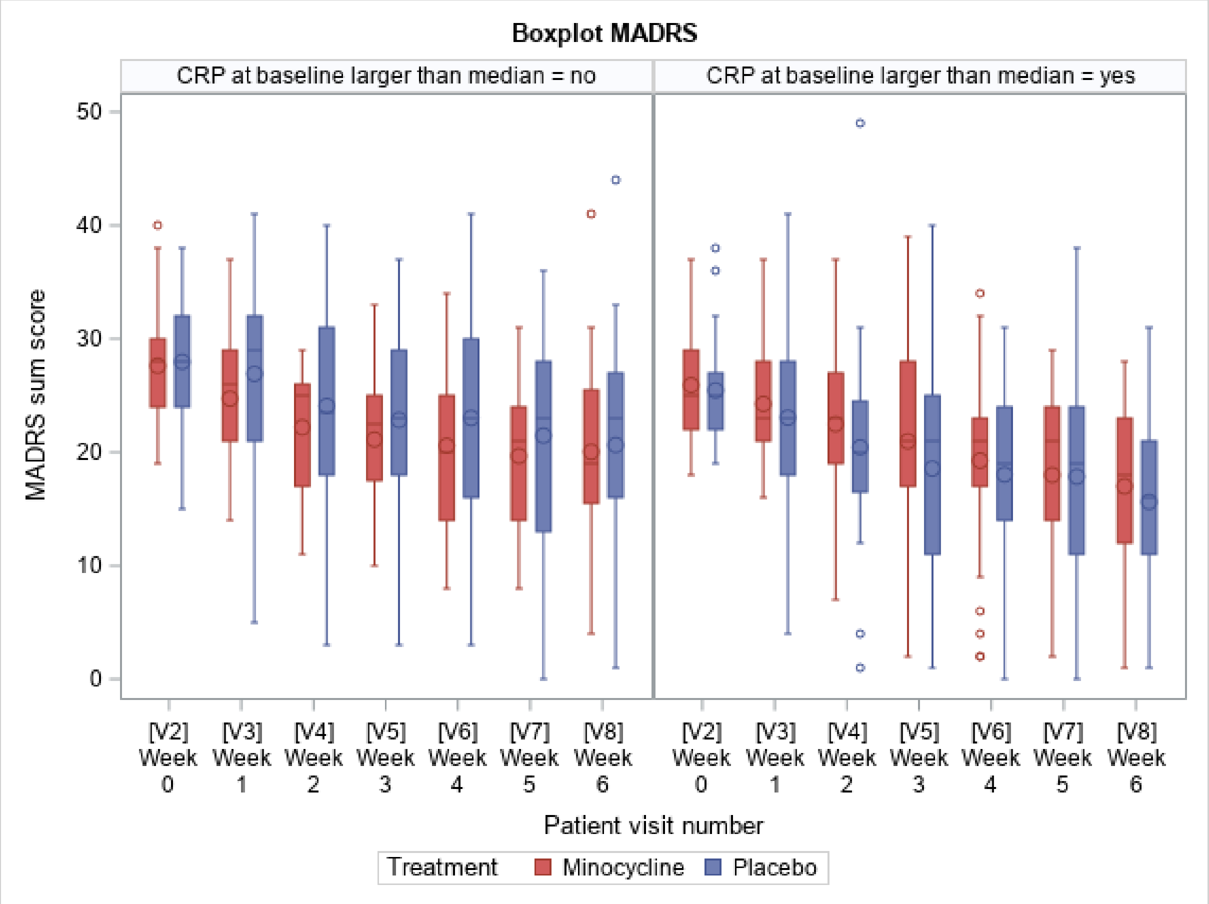


© 2022 Hellmann-Regen J et al. *JAMA Network Open.*

**eFigure 13. BMI**

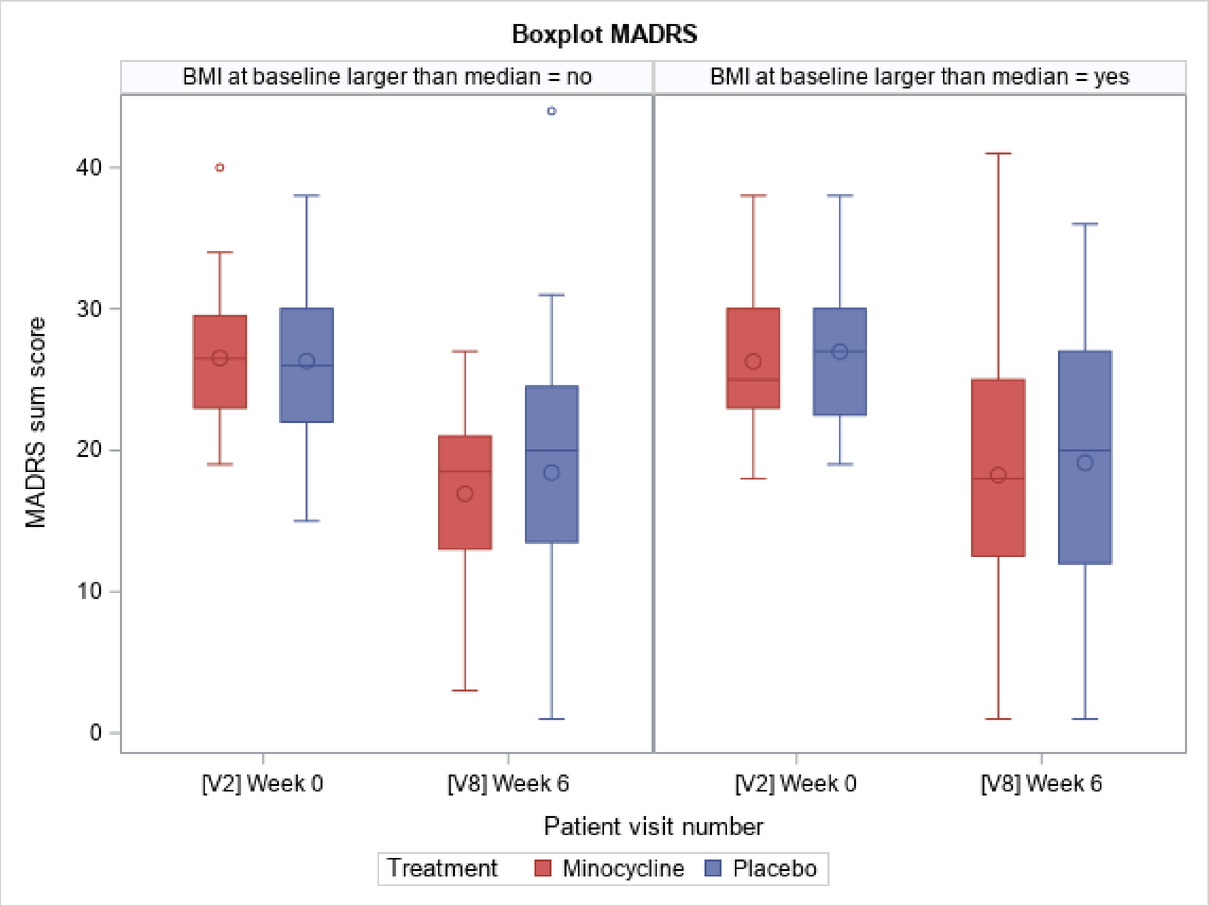


© 2022 Hellmann-Regen J et al. *JAMA Network Open.*

**eFigure 14. CTQ**

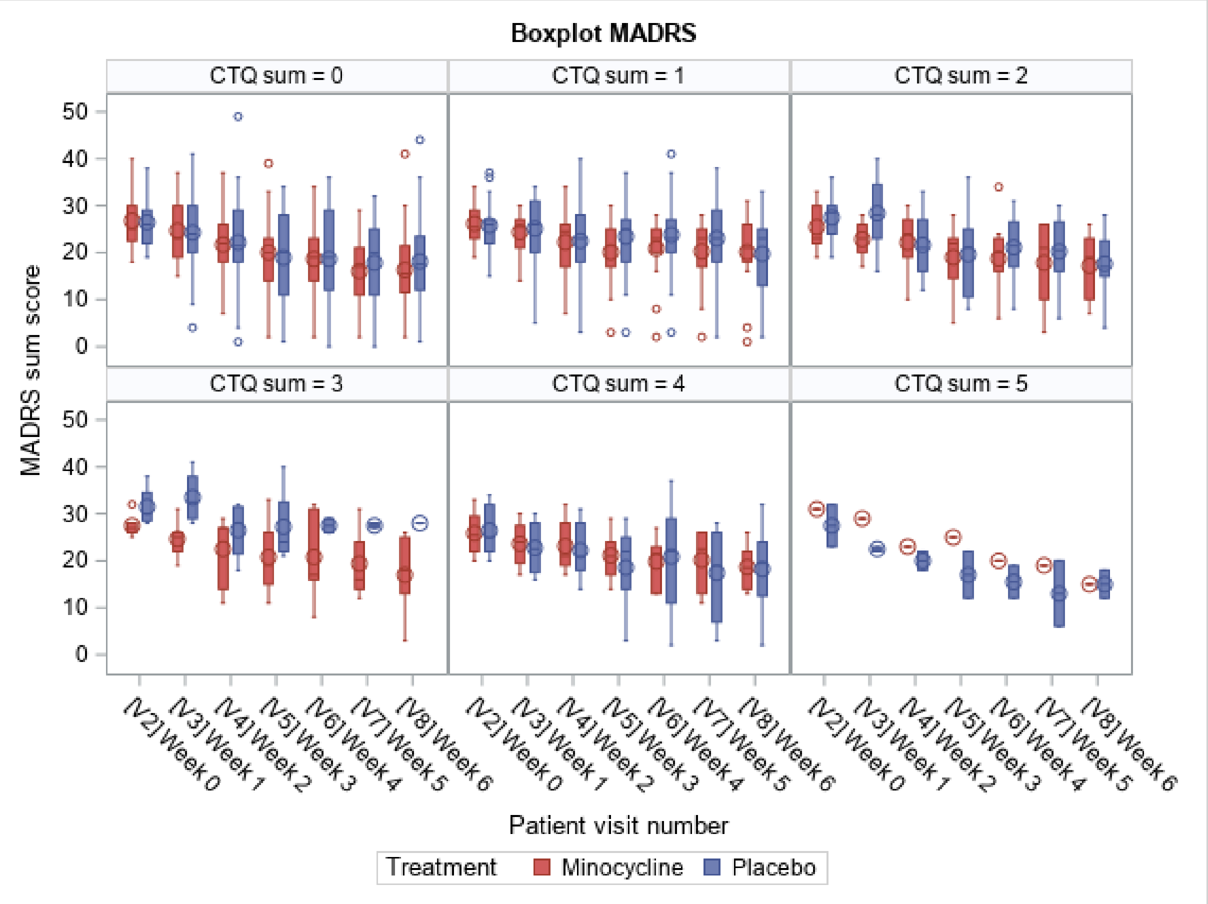


© 2022 Hellmann-Regen J et al. *JAMA Network Open.*
