# Supplementary material for: Effect of Minocycline on Depressive Symptoms in Patients With Treatment-Resistant Depression: A Randomized Clinical Trial
Source: JAMA Netw Open. 2022 Sep 14;5(9):e2230367. doi: 10.1001/jamanetworkopen.2022.30367 (PMC9475381; doi:10.1001/jamanetworkopen.2022.30367)
Supplement: Supplement 3. — Data Sharing Statement [file jamanetwopen-e2230367-s003.pdf]

Data Sharing Statement

Hellmann-Regen J, Clemens V, Grözinger M, et al. Effect of minocycline on depressive symptoms in patients with treatment-resistant depression: a randomized clinical trial. *JAMA Netw Open*. 2022;5(9):e2230367. doi:10.1001/jamanetworkopen.2022.30367

Data
Data available: Yes
Data types: Deidentified participant data
How to access data: isabella.heuser@charite.de
When available: With publication

Supporting Documents
Document types: None

Additional Information
Who can access the data: researchers whose proposed use of the data has been approved
Types of analyses: for secondary analyses, metaanalyses
Mechanisms of data availability: with investigator support
